# CO_2_ Adsorption and Photocatalytic Reduction Mechanisms on TiO_2_‑Terminated CaTiO_3_(100): A Density Functional Theory Study

**DOI:** 10.1021/acs.jpcc.5c04650

**Published:** 2025-10-28

**Authors:** Onofrio Tau, Giacomo Giorgi, Riccardo Rurali

**Affiliations:** † 54449 Institut de Ciència de Materials de Barcelona, ICMAB-CSIC, Campus UAB, 08193 Bellaterra, Spain; ‡ Department of Civil and Environmental Engineering (DICA), 9309 Università degli Studi di Perugia, Via G. Duranti 93, 06125 Perugia, Italy; ¶ CNR-SCITEC, I-06123 Perugia, Italy; § CIRIAF - Interuniversity Research Centre, University of Perugia, 06125 Perugia, Italy; ∥ Centro S3, CNR-Istituto Nanoscienze, Via G. Campi 213/a, Modena 41125, Italy

## Abstract

Photoreduction is an important approach aimed at reducing the CO_2_ atmospheric content, which is responsible for global warming. The development of an efficient photocatalyst can strongly improve the efficiency and selectivity of the byproducts of such a process. Recently, CaTiO_3_ has been used as an alternative semiconductor catalyst due to its attractive properties. In this study, we present first-principles electronic structure calculations to investigate the general reaction mechanism that leads to the main value-added HCOOH, CO, CH_3_OH, and CH_4_ byproducts, focusing on the reactions of the adsorption, activation, and reduction reactions of molecules on the TiO_2_-terminated CaTiO_3_ (100). Our results show that CO_2_ can be activated by charge transfer of excess electrons, leading to a CO_2_^·–^ anion that can give the formate (HCOO^–^) intermediate by first reduction. However, the second hydrogenation leading to HCOOH is impeded by the prohibitive energy barrier; in particular, activated CO_2_ can also easily undergo decomposition, which facilitates CO production. Afterward, we discuss the possible reaction mechanisms of CO photoreduction toward CH_3_OH and CH_4_ value-added products, taking into account the experimental evidence that only CO and CH_4_ have been detected. The reaction pathway generally follows the most energetically convenient routes characterized by activated intermediates. Although CH_3_OH could finally be produced, its strong adsorption and promoted decomposition to CH_3_O + H on the surface could explain why it has not been detected, compared to the more volatile CH_4_ molecule, ascribed by its nonpolar nature.

## Graphical abstract

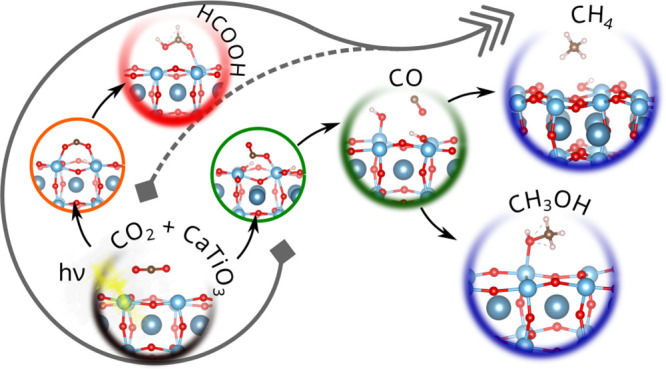

## 1 Introduction

The greenhouse effect has been responsible for global warming, mainly due to the massive release of carbon dioxide (CO_2_) by human activities, which is recognized as a major greenhouse gas.^1,2^ To alleviate the atmospheric content of CO_2_ and avoid severe environmental problems, CO_2_ conversion in value-added chemicals by photocatalysis^3–7^ has been proposed and gained increasing attention during the last decades. Inspired by natural photosynthesis, CO_2_ is consumed by catalytic hydrogenation using solar light that activates the semiconductor photocatalysts generating electron–hole pairs; then, these charge carriers will be used to reduce or oxide CO_2_ and its reaction intermediates by charge transfer to enable the production of energy-rich chemicals like methane (CH_4_), methanol (CH_3_OH), formic acid (HCOOH) and other hydrocarbons that can be exploited as chemical feedstock and fuels.^8^ Moreover, the process can be considered environmentally friendly as it is also typically carried out at ambient reaction conditions since other transformation methods involve high process operational cost because of high pressure and temperature regimes.^9–11^ Although important progress has been made since the first report,^12^ the photocatalytic reduction of CO_2_ is still far away from industrial applications due to the low catalytic conversion and poor product selectivity.^3,13^ This can be explained by two main reasons: (i) CO_2_ is highly themodynamically and kinetically stable due to its electronic structure; (ii) the CO_2_ photoreduction to other products can be encouraged by its activation, namely by bending the molecule in its initial linear shape on the catalytic surface, creating dipole moments and thus enhancing the chemical reactivity, but this typically requires a very high energy cost.^14^

Several researchers have reported the use of different photocatalysts, since the development of an efficient catalyst can strongly improve the CO_2_ photoreduction efficiency and selectivity.^15–20^ Among many semiconductors, titanium dioxide (TiO_2_) has been commonly employed due to its exceptional CO_2_ photoreduction activity.^2,21–26^ Recently, CaTiO_3_ has attracted interest as an alternative semiconductor photocatalyst^27–30^ owing to its low cost, great chemical stability, no toxicity, and resistance to photocorrosion. Moreover, it can be easily prepared by many methods, with the hydrothermal and solvothermal processes being the most popular ones.^31,32^ The CaTiO_3_ perovskite is an *n*-type chemically stable semiconductor with a wide bandgap of 3–3.5 eV,^33,34^ which allows the absorption of UV light. However, the latter represents only a small fraction of the solar spectrum (4% of solar energy) and, along with the high recombination rate of electron–hole pairs, it can severely limit the application of this material.^35^ In contrast, it is well-known that CaTiO_3_ has good adsorption features, and some works have been dedicated to pure CaTiO_3_ as a substrate for the photoreduction of CO_2_. Kwak et al.^36^ were the first to experimentally investigate the CO_2_ photocatalytic activity of Ca_*x*_Ti_*y*_O_3_ nanoparticles (*x* = 1.50, 1.28, 1.00, and 0.85; *y* = 0.75, 0.85, 1.00, and 1.28) with H_2_O. The observed Ca_*x*_Ti_*y*_O_3_ particles were orthorhombic, and the final byproduct detected by gas chromatography was only CH_4_. The best photocatalytic performance (methane formation rate of ∼17 μmol/g_catal_ for 7 h) was achieved with Ca_1.00_Ti_1.00_O_3_. Im et al.^37^ used synthesized CaTiO_3_ with various Ca:Ti ratios coated onto basalt fibers as a substrate. Gas chromatography analysis detected a formation rate of ∼3.7 and ∼11.4 μmol/g in 8 h relative to CH_4_ and CO for CaTiO_3_BF, respectively, which increased to a maximum of ∼17.8 and ∼73.1 μmol/g for CaTiO_3_(1.5:1)­BF. As suggested by the authors of that study, the latter catalytic sample has the highest amount of TiO_2_ and oxygen vacancies along with CaTiO_3_ on basalt fibers among all synthesized samples: all the excited electrons from irradiation move to oxygen vacancy sites in the rutile TiO_2_, where CO and CH_4_ are preferably produced from CO_2_, which could explain the best performance achieved. Many computational studies have been reported to complement experimental works on TiO_2_-based catalysts by providing atomistic insights underlying the mechanism of CO_2_ photoreduction, which mainly cover adsorption, activation, and reaction pathways occurring on the surface.^38–43^ In contrast, no similar investigation has been addressed yet with a pure CaTiO_3_ surface, neither CO_2_ adsorption studies nor early reduction reactions. The (100) and (110) surfaces are typically the most studied and often the most relevant for applications such as photocatalysis and biomedical applications because they are the lowest-energy and the most stable crystal planes.^44^ The (100) surface of CaTiO_3_ can have two distinct surface terminations, namely CaO and TiO_2_. These different terminations significantly influence the chemical and photochemical reactivities of the surface. For example, different facets with varying terminations can promote different reaction pathways, such as reduction or oxidation, which is vital for controlling photocatalytic reactions.^27^ In particular, the TiO_2_-terminated (100) surface has been identified as favorable and stable in theoretical studies,^45^ providing a basic surface environment that facilitates the adsorption and activation of CO_2_.^46^

In this work, we report on the density functional theory (DFT) investigation of CO_2_ photoreduction reactions on pure TiO_2_-terminated CaTiO_3_(100) for the first time with the aim of elucidating the overall catalytic pathway that leads to the formation of HCOOH, CH_3_OH, and the experimentally detected CO and CH_4_ byproducts. First, we investigate the adsorption of CO_2_ and its related intermediates and then analyze the energetics (i.e., activation energy barriers) of likely and rate-limiting reaction steps. The present study may have potential impact in several technological applications where CaTiO_3_ plays a primary role such as, among the many, (i) photocatalytic reduction of greenhouse gases;^36^ (ii) dye photodegradation of CaTiO_3_-based heterostructured photocatalysts;^47,48^ (iii) CO_2_ capture and separation^49^ and (iv) in nonplasma technology (CO_2_ conversion in reactors,^50^ where CaTiO_3_ is an efficient material for dielectric barrier discharge).

## 2 Computational Model and Methods

First-principles calculations based on DFT were carried out with the Quantum ESPRESSO (QE) code (version 7.3).^51–53^ The generalized gradient approximation (GGA) in the Perdew–Burke–Ernzerhof (PBE) flavor was used for the exchange-correlation functional,^54^ along with ultrasoft pseudopotentials (the following electrons were explicitly treated as valence: Ca 3*s* ^2^3*p* ^6^4*s* ^2^, Ti 3*s* ^2^3*p* ^6^3*d* ^2^4*s* ^2^, O 2*s* ^2^2*p* ^4^, C 2*s* ^2^2*p* ^2^, and H 1*s* ^1^). To account for van der Waals (vdW) interactions between molecules and the crystal surface, the vdW correction terms (DFT-D3) by Grimme et al.^55^ were used. In the DFT-D3 scheme, an analytical pairwise atomic potential is numerically added at the end of each self-consistent energy minimization cycle to the energy values obtained from the Kohn–Sham potential energy functional; the additional computational cost is almost negligible as opposed to vdW-enabled exchange-correlation functionals (vdW-DF).

CaTiO_3_(100) can have two different surface terminations, namely CaO and TiO_2_. In this work, a TiO_2_-terminated CaTiO_3_(100) slab in the periodic supercell approach was used to study the adsorption and reduction reactions of CO_2_. To mimic the crystal surface, we employed a 2 × 2 surface supercell, containing a symmetric five-layer slab (16 Ca atoms, 24 Ti atoms, and 64 O atoms) with a 15 Å thick vacuum region to avoid spurious interactions between periodic images. The slab was built from the PBE-optimized orthorhombic CaTiO_3_ primitive cell (Pbnm space group) with lattice constants *a* = 5.394 Å, *b* = 5.497 Å, *c* = 7.679 Å, which are less than 1% larger than the experimental values of *a* = 5.372 Å, *b* = 5.463 Å, *c* = 7.636 Å. The cutoff energies of 45 and 450 Ry were used for the orbitals and the charge density, respectively, and the Γ point was sufficient for Brillouin zone sampling. Notice that the DOS reported in Figure S1 in the Supporting Information, featuring a Fermi energy well within the band edges, allows us to conclude that no partial charge transfer occurs (a possible artifact of nonstoichiometric slabs). The geometries and ground-state energies of adsorbed molecules on the crystal surface have thus been investigated in the low-coverage regime (one molecule for eight surface Ti atoms), with the molecule positioned on only one side of the slab. During geometrical optimization, the two bottom layers were kept fixed, while the remaining layers with the adsorbed molecule were allowed to relax in all cases until the total energy and forces were less than 10^–4^ Ry (0.0014 eV) and 10^–3^ Ry/Bohr (0.026 eV/Å), respectively. We checked that CO_2_ and CO molecules in various adsorption configurations do not create a significant nonflat potential in the vacuum layer of the simulation cell and thus opted not to include dipole corrections. The slab thickness and the supercell lateral dimension were chosen large enough to sufficiently mimic the bulk phase and to avoid spurious interactions among periodic images since periodic boundary conditions were applied. This was verified by looking at the convergence of the adsorption energy, *E*_ads_, as a function of the number of slab layers (see Supporting Information, Table S1). The adsorption energy *E*_ads_ is defined as

$$E_{\mathrm{ads}}=E_{\mathrm{tot}}(\mathrm{molecule}+\mathrm{slab})-[E_{\mathrm{tot}}(\mathrm{molecule})+E_{\mathrm{tot}}(\mathrm{slab})]$$

where *E*_tot_(molecule + slab), *E*_tot_(molecule), and *E*_tot_(slab) are the ground-state total energies of the adsorbate–surface system, the isolated molecule in the gas phase, and the clean surface, respectively. The adsorption energy provides a measure of whether a molecule is physisorbed or chemisorbed, with the most stable structures corresponding to the most negative values.

Since DFT suffers from the self-interaction error, which typically leads to erroneous overdelocalization of strongly localized electrons in CaTiO_3_, and to provide a more accurate description of the CO_2_ activation and photoreduction reactions, the Hubbard correction^56,57^ was first accounted in this work by looking at the lattice constants of the bulk crystal and *E*_ads_ of various (inactivated or activated) adsorbed CO_2_ configurations as the target properties. For bulk CaTiO_3_, the effective Hubbard parameter *U* of 4.61 eV for the 3d Ti hybrid orbitals was initially computed by density functional perturbation theory implemented in the hp.x code^58^ of QE, which corresponds to a calculated band gap of 3.00 eV (2.39 eV without Hubbard) that is consistent with experimental values in the range 3.0–3.5 eV.^30^ However, both the lattice constants of the CaTiO_3_ primitive cell and *E*_ads_ values were found to be almost unchanged with respect to the case of *U* = 0 eV (see Supporting Information, Tables S1 and S2) and thus all the results presented thereafter were calculated without including the Hubbard parameter. Moreover, *U*-values are usually chosen based on the accuracy in reproducing bulk properties like lattice parameters and band structure, while using bulk-calculated *U* values to investigate chemical reactions on transition metal oxide surfaces may not describe reaction energetics correctly.^59^ Hence, a comprehensive investigation of the effect of *U* on surface chemical reactions is needed, and it is beyond our scope. Although more sophisticated exchange-correlation functionals than PBE can give more accurate results, they would inevitably require unaffordable computational costs.

The transition state (TS) configuration, the energy barrier *E*_b_ (i.e., activation energy), and the minimum energy path (MEP) of the chemical reactions were computed using the climbing image nudged elastic band (CI-NEB) method^60^ using a convergence force threshold of 0.05 eV/Å for optimization. The initial state and final state (FS) were deduced from single-geometry optimization calculations, and a sufficient number of intermediate images was chosen to guarantee an interimage distance below 1.5 Å.

## 3 Results and Discussion

Experimental investigations have reported on the detection of carbon monoxide (CO) and methane (CH_4_) as value-added byproducts of the CO_2_ photoreduction using the CaTiO_3_ catalyst,^36,37^ and in this work, we consider such processes in detail. Even though other plausible chemicals, such as methanol (CH_3_OH) and formic acid (HCOOH), have not been detected, we also study the overall reaction mechanisms leading to the formation of such compounds to assess the feasibility of these photoreduction reactions or elucidate what prevents them from occurring.

### 3.1 Adsorption of CO_2_

The adsorption and activation of CO_2_ on TiO_2_-terminated CaTiO_3_(100) were first investigated as early steps of the photoreduction process. To find different energetically stable adsorbed configurations on the crystal surface, the CO_2_ degrees of freedom were progressively varied, followed by geometry optimization to eventually achieve new minimum energy structures. This investigation was extended to identify further configurations depending on the adsorption site, i.e., on top of surface Ti and O atoms. Moreover, the surface O atom, indicated as O_s_ thereafter, can be differentiated by the way it points inward or outward to the crystal bulk, as labeled in Figure 1a by O_s_^in^ and O_s_^out^, respectively. The activation of CO_2_ (or any CO_2_-derived intermediate) by charge transfer of the photoexcited electron to the molecule, as

$${\mathrm{CO}}_{2}+e^{-}\to {\mathrm{CO}}_{2}^{-}$$

was simulated by introducing adsorbed H atoms on top of O_s_. In fact, the artificial photoexcited electron available for the charge transfer from the surface to the molecule is drawn from the adsorbed H atom: the latter becomes a proton with an apparent charge state +1 when its electron is redistributed over Ti atoms to populate the bottom of the CaTiO_3_ conduction bands. Löwdin charge analysis confirmed that the excess electron drawn from the H homogeneously redistributes over the 3d orbitals of Ti atoms (see also Figure S2 in the Supporting Information). Some methods that go beyond ground-state DFT, such as time-dependent DFT, allow the direct modeling of excited electrons; others just introduce the excess electron by modifying the total charge of the system or defects that can act as electron donors. However, the modeling of the photocatalyst excited state by introducing the excess electron drawn from the adsorbed H atom is widely accepted in the literature^41,43,61–63^ and involves a more affordable computational cost. Moreover, protons can be directly produced by water oxidation in artificial photosynthesis, and as a result, it is equivalent to using a surface proton to simulate hydrogenation reactions in theoretical calculations. On the other hand, the hydrogen evolution reaction (2H^+^ + 2*e* ^–^ → H_2_) is known to be the competing process of CO_2_ photoreduction, which decreases its conversion efficiency to value-added byproducts. Various strategies have been developed to suppress the hydrogen evolution reaction.^64^ However, a comprehensive investigation of such a competing mechanism on CaTiO_3_(100) is beyond the scope of this work and should be addressed by further studies. Calculations of the atomic spin moments of activated CO_2_^·–^ were performed to deduce how the photoexcited electrons apparently redistribute in the molecule. Indeed, a nonzero atomic spin moment is descriptive of the photoexcited electron localization on that atom. In addition, charge transfer was also qualitatively demonstrated by removing the adsorbed H atoms from CO_2_^·–^-containing supercells, followed by geometry optimization: if a new configuration of inactivated CO_2_ is reached, it means that such H atoms were essential to stabilize the radical anion CO_2_^·–^. The chemical states in which charge transfer to the molecule occurs are labeled with an asterisk thereafter.

**Figure 1:**
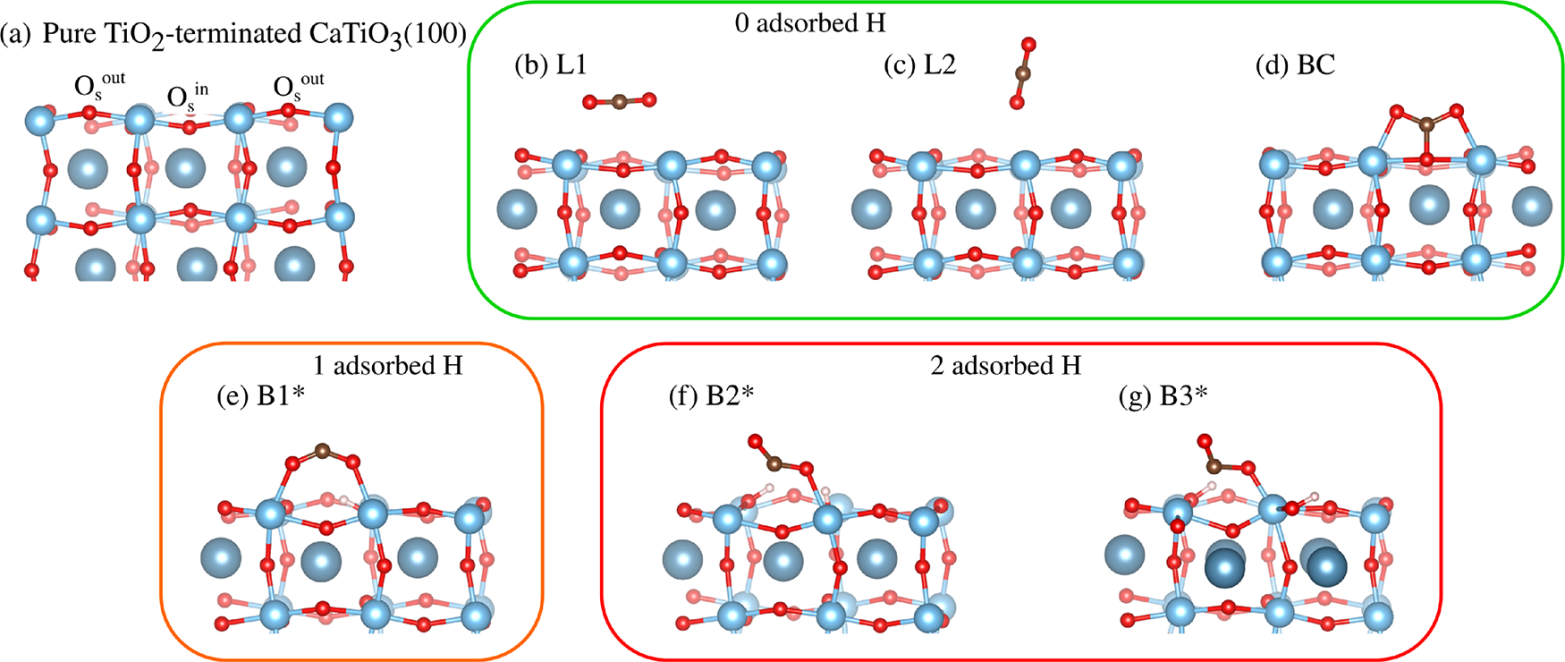
Adsorption configuration of CO_2_ on TiO_2_-terminated CaTiO_3_(100): (a) pure CaTiO_3_ surface; (b) horizontal linear adsorption “L1”; (c) vertical linear adsorption “L2”; (d) bridged carbonate “BC” adsorption; (e–g) Bent adsorption “B1*,” “B2*,” and “B3*.” Activated configurations are labeled with asterisks.

The obtained adsorbed configurations are shown in Figure 1b–g, while the CO_2_ adsorption energies and geometrical parameters are summarized in Table 1. When it physisorbs, the CO_2_ molecule can lie horizontally or vertically on the crystal surface, as represented by the L1 and L2 structures in Figure 1b,c, respectively. The physisorption nature of the interaction is reflected by the low value of the adsorption energy, which does not exceed −0.43 eV, and by the unperturbed geometry of CO_2_: the C=O double bond still preserves its length of 1.18 Å along with the molecule linearity (bond angle of 176.8°), similar to the gas phase. Moreover, the surface-molecule distance typically exceeds ∼2.5 Å. In the Bridged Carbonate (BC) structure, CO_2_ strongly bonds with the Ti–O_s_–Ti triplet in the lowest-energy bridged-like configuration (see Figure 1d) as confirmed by its higher *E*_ads_ of −1.27 eV. The resulting C–O_s_ and Ti–O bonds have lengths of 1.35 and 2.13 Å, which are consistent with the estimated single bond values of ∼1.4 and ∼2.1 Å, respectively.^65,66^ The CO_2_ bond angle is 131.5°, which also confirms the strong interaction with the surface.

**Table 1:** Adsorption Energy, CO_2_ Bond Lengths, and Molecular Angle of the Various Adsorption Configurations^a^

conf.	*E*_ads_ (eV)	C–O_1_ (Å)	C–O_2_ (Å)	O_1_–C–O_2_	C–O_s_ (Å)	Ti–O_1_ (Å)	Ti–O_2_ (Å)	Ti–C (Å)
BC	–1.27	1.27	1.28	131.5°	1.35	2.13	2.16	
L1	–0.43	1.18	1.18	176.8°	2.67	2.72	2.59	
L2	–0.27	1.18	1.17	178.7°		2.49	4.66	3.55
B1*	+0.09	1.25	1.24	136.9°	2.95	2.13	2.12	
B2*	+0.42	1.27	1.21	139.4°	2.66	2.16	3.32	2.86
B3*	+0.27	1.42	1.21	120.0°	2.61	1.87	3.08	2.29

a Activated configurations are labeled with an asterisk.

The adsorption surface selectivity plays an important role, since CO_2_ structures can preferentially originate only on certain surface sites that mainly depend on the O_s_ type, i.e., O_s_^in^ and O_s_^out^. Indeed, BC will more likely be produced when CO_2_ gets closer to O_s_^out^; otherwise, linear configurations, such as L1 and L2, can be observed above O_s_^in^. When adsorbed H atoms are also accounted for, three activated structures were obtained and labeled with the letter “B” (which stands for ‘Bent’) in Figure 1e–g. The B1* configuration in Figure 1e is a well-known structure along with BC that is largely reported on theoretical studies of CO_2_ photoreduction using TiO_2_ or other perovskites.^40,41,63,67,68^ In our simulation, the molecule arranges in a bridge-like structure above the surface, interacting with two neighboring Ti by its two O atoms, while the unpaired photoexcited electron mainly localizes on the C atom because of its calculated spin-polarized moment of 0.6, as also occurs on TiO_2_. The *E*_ads_ of the B1* state (+0.09 eV) is significantly higher than that of BC (−1.27 eV) as a result of the molecular electronic reorganization that converts the linear CO_2_ molecule to the bent radical anion CO_2_^·–^. The CO_2_^·–^ bond angle decreases significantly to 136.9° and C–O elongates to 1.25 Å, the latter indicating that the double bond character is partially lost, as occurs in BC. Due to adsorption selectivity, the B1* state only originates on top of the O_s_^in^, whereas the C–O_s_^out^ interaction is promoted in the other case, which then leads to the BC state. The B2* and B3* configurations in Figure 1f,g are the last two activated states identified in this work, which resemble the “Bidentate Carbonate” structure^20,40^ because of their chair-like geometrical arrangement. In the Bidentate Carbonate species, the CO_2_ molecule interacts with the Ti–O_s_ pair via both the O and C atoms, respectively. In contrast, only one O of CO_2_^·–^ takes part to bonding with the underneath Ti atom (Ti–O distance of 2.16 Å) in B2* as the C–O_s_ distance is 2.66 Å, while in B3* both C and O interacts with two neighboring Ti atoms (Ti–C and Ti–O distance of 2.29 and 1.87 Å, respectively) resulting in the C–O bond stretching up to 1.42 Å, thus completely losing its double bond character, and different CO_2_^·–^ molecular angle of 120.0°. As opposed to the B2* states, the distance of the interacting Ti–Ti pair in B3* is shortened by around 0.2 Å due to steric effects with respect to the case of the clean CaTiO_3_ surface. B2* and B3* are the least stable activated structures with *E*_ads_ of +0.42 and +0.27 eV, respectively. Both states can be formed when the molecule locates on top of O_s_^in^, as in B1*, while the BC state is still favored on O_s_^out^. By atomic spin moment analysis, the excess electron cloud in CO_2_^·–^ almost equally redistributes between the C and the unperturbed O. In contrast to B1*, where only one adsorbed H atom was sufficient to stabilize the activated configuration, two H atoms were essential to produce both B2* and B3* structures: by just removing one of them, followed by geometry optimization, linear CO_2_ configurations were obtained.

### 3.2 Photoreduction of CO_2_ to HCOOH and CO

To correctly predict and account for the most likely conversion pathways that involve a series of reduction or dissociation reactions from CO_2_ to HCOOH or CO, we have considered different CO_2_ adsorption configurations as initial reaction guesses. The BC was found as the lowest-energy state (*E*_ads_ of −1.27 eV), but the hydrogenation on its O would require a rather expensive cost to overcome the energy barrier since the O atom must first detach from the surface; as a result, it would be unfeasible under typical experimental reaction conditions. The poor reactivity of CO_2_ in BC is due to its strong adsorption on the surface. Although conversion into the bent radical anion CO_2_^·–^ is affected by high energy costs, there is a widespread consensus that activated molecules are more prone to be reduced, and therefore, they are the starting points for the CO_2_ photoconversion. For this reason, only the activated “B” structures were considered in the overall mechanism thereafter. In this work, each reduction reaction was studied by introducing more than one adsorbed H atom into the system: the first is directly involved in catalytic hydrogenation, while the remaining contributes to charge transfer processes.

When the proton approaches the bent anion CO_2_^·–^, it can bind to the C or O atom: the formate (HCOO^–^) species adsorbed on the surface is produced in the first case, while the COOH intermediate is produced in the second case. Afterward, HCOOH could finally be generated if a second reduction reaction occurs. The MEPs that lead to HCOO^–^ from the CO_2_^·–^ in B1* and B3* are shown in Figure 2a. For the B2* state, it was observed that CO_2_^·–^ preferably moves first to the more stable B3* state before being hydrogenated on its C, since only 0.08 eV is required to overcome the reaction barrier of the B2*-to-B3* conversion. The *E*_b_ is 0.36 eV for the B1* case, which slightly lowers to 0.29 eV for B3*. Moreover, the higher chemical stability of HCOO^–^ with respect to the Bent ‘B’ states by around 1.8–2.0 eV hinders the reduction from go backward. In the B3* mechanism, the molecular movement is almost entirely dictated by the proton, as it directly locates on top of the CO_2_^·–^, while the molecule in B3* rotates 180° to allow its free O atom to bind to the surface and then give formate. It should be noted that both pathways lead to the same final HCOO^–^ configuration, which preserves the B1* geometry with a C–O distance of 1.27 Å and the Ti–O bond length that slightly changes from 2.13 to 2.07 Å. As a result of the C–H bond formation, the molecular angle reduces to 129.0°. By computing and comparing Löwdin charges of equivalent H-reduced and H-unreduced systems, we concluded that charge transfer to HCOO^–^ did not take place. Proceeding to the second reduction step leading to HCOOH (Figure 2a), we calculated a prohibitive *E*_b_ of 1.59 eV. Even though HCOOH might be formed, the inexpensive reverse reaction (*E*_b_ of 0.29 eV) promotes its decomposition to HCOO^–^ + H^+^.

**Figure 2:**
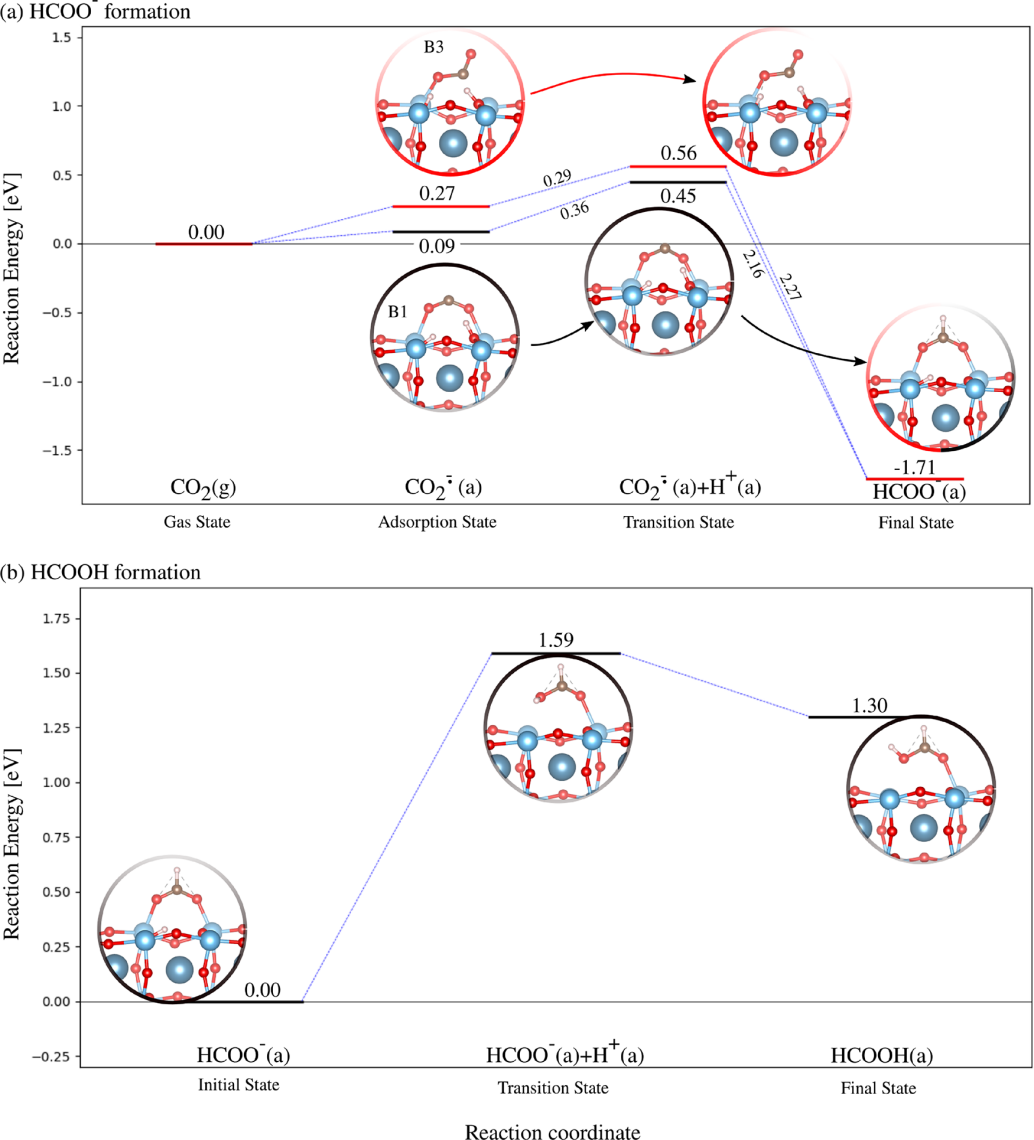
Photoreduction of CO_2_^·–^ to HCOOH on TiO_2_-terminated CaTiO_3_(100): reaction pathways leading to (a) HCOO^–^ from CO_2_^·–^ in B1* and B3*; and (b) HCOOH from HCOO^–^. Activated configurations are labeled with asterisks.

In order to investigate the formation of the COOH intermediate by reduction, we attempted to achieve the FS by progressively approaching the proton to the O of the CO_2_^·–^, followed by geometry optimization. However, the spontaneous decomposition of CO_2_^·–^···H to CO + OH was observed instead of the reduction that would have led to COOH. Thus, we studied the decomposition mechanism of CO_2_^·–^ in B1* and B3*. The MEPs are shown in Figure 3 as combinations of two consecutive elementary steps: (i) first, the CO_2_^·–^ dissociates to give CO + O and (ii) the hydroxyl (OH^–^) species is then produced by proton attachment to the dissociated O. Only 0.16 eV is required for the B3* decomposition of the first step, which increases to 0.45 eV for B1*, although the latter reactant state is energetically more stable than the former by around 0.18 eV. The discrepancy in the activation energy values could be attributed to the nature of the surface-molecule interaction. First, it mainly depends on the dissociated C–O bond strength. Since it elongates to 1.42 Å upon adsorption in B3*, which has a single bond character, the energy demand to break the C–O bond is lower with respect to the B1* case, whose C–O bond (1.24 eV long) partly preserves its double bond character. Second, the Ti–C interaction in B3* enhances the CO production, as opposed to the B1* dissociation, in which the as-formed CO adsorbs via its O atom. As it will be explained in the following, CO preferentially physisorbs on top of Ti with its C, as it occurs in the intermediate state of the B3* pathway. Following the second elementary step, the OH species is finally produced with an energy cost of 0.06 eV. Note that the *E*_b_ values of the first elementary step and its reverse reaction (i.e., the recombination of CO and O) are almost equal, which indicates that a chemical equilibrium could be reached; namely, the concentrations of CO_2_^·–^ and decomposed species (CO + O) are comparable. However, the recombination can be hindered by its cheaper competitive process (i.e., the formation of OH species), thus proceeding to the final CO + OH state that is more stable than the intermediate state by around 0.7 eV. The CO + OH recombination was also investigated as it could constitute an alternative pathway to go back to the initial CO_2_^·–^ state. However, the mechanism was not observed due to the unreactive adsorbed OH. From the above analysis, the HCOO^–^ and CO species can likely be the most accessible intermediates of the early reduction and decomposition reactions. Afterward, CO could be further reduced to give value-added byproducts such as CH_4_ and CH_3_OH. In contrast, the high energy cost of the second hydrogenation step of HCOO^–^ and the proclivity of HCOOH to decompose could explain why formic acid has not been detected in CO_2_ photoreduction experiments using CaTiO_3_.^36,37^

**Figure 3:**
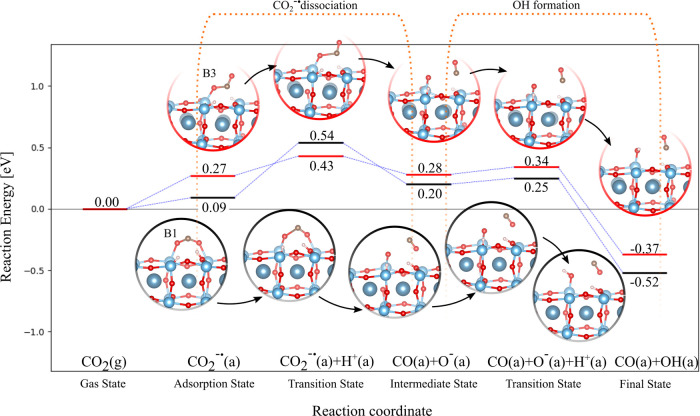
Photoreduction of CO_2_^·–^ in B1* and B3* to CO on TiO_2_-terminated CaTiO_3_(100). Activated configurations are labeled with asterisks.

### 3.3 Adsorption of CO

Before proceeding with the investigation of the CO photoreduction mechanisms, CO adsorption in the spirit of the previous paragraph on CO_2_. The minimum energy adsorption configurations of CO are all shown in Figure 4, and their respective geometrical parameters are summarized in Table 2. Physisorption occurs when CO is vertically located on top of Ti and weakly interacts with the surface through its C or O, as in the v1 and v2 states, respectively (see Figure 4a,b). The Ti–C interaction in v1 is promoted over Ti–O due to the lower *E*_ads_ of −0.49 eV, which decreases to −0.19 eV for the v2 state. The molecule–surface distance of 2.37 Å in v1 can not be representative of chemisorption since the Ti–C bond in organotitanium compounds is ∼2.1 Å long.^66^ Moreover, the unperturbed C–O bond is 1.14 Å long, as in the gas phase. When it chemisorbs, CO moves to the v3, h1, h2, or v4* states, as shown in Figure 4c–f, respectively. Despite a low *E*_ads_ of −0.23 eV, CO in v3 forms a bridging bond with the Ti–O_s_ pair via its C being the Ti–C and C–O_s_ distances of 2.11 and 1.38 Å long, respectively. In addition, the CO bond length is elongated to 1.21 Å. The h1 state is the lowest-energy CO configuration with *E*_ads_ of −0.61 eV. Although the same C bridging bond in v3 is also found in h1, the higher chemical stability of the latter state originates from the Ti–O covalent bond (distance of 2.16 Å), which further perturbs the C–O bond that now lengthens to 1.26 Å. Due to the steric effects of chemisorbed CO, the spatial separation of the interacting Ti–Ti pair increases by ∼0.4 Å. CO in h2 shares the same bond’s spatial arrangement and similar geometrical parameters of h1, but a lower *E*_ads_ of −0.09 eV was computed. This energy discrepancy between h1 and h2 can be explained by the adsorption selectivity of O_s_: the CO adsorption is enhanced when the C atom locates on top of O_s_^in^, thus preserving the alternate O_s_^out^–O_s_^in^–O_s_^out^ tuple as in h1 (see dashed green line in Figure 4d); contrarily, when CO approaches O_s_^out^ upon adsorption, the less chemically stable O_s_^in^–O_s_^in^–O_s_^in^ tuple could then be formed as in h2 (see dashed green line in Figure 4e). As four adsorbed H atoms are introduced, charge transfer occurs to CO, which moves to the v4* state. By atomic spin moment analysis, the excess electron is equally distributed on both atoms. Notice that the simultaneous adsorption of four H atoms on the surface is perhaps not an entirely realistic model, but this is the minimum number needed to observe charge transfer to the CO molecule. v4* and v1 share the same geometric structure, but the Ti–C interaction in the activated configuration is stronger, as evidenced by its reduced bond length of 2.17 Å (with respect to 2.37 Å in v1) and the lower *E*_ads_ of −0.52 eV.

**Figure 4:**
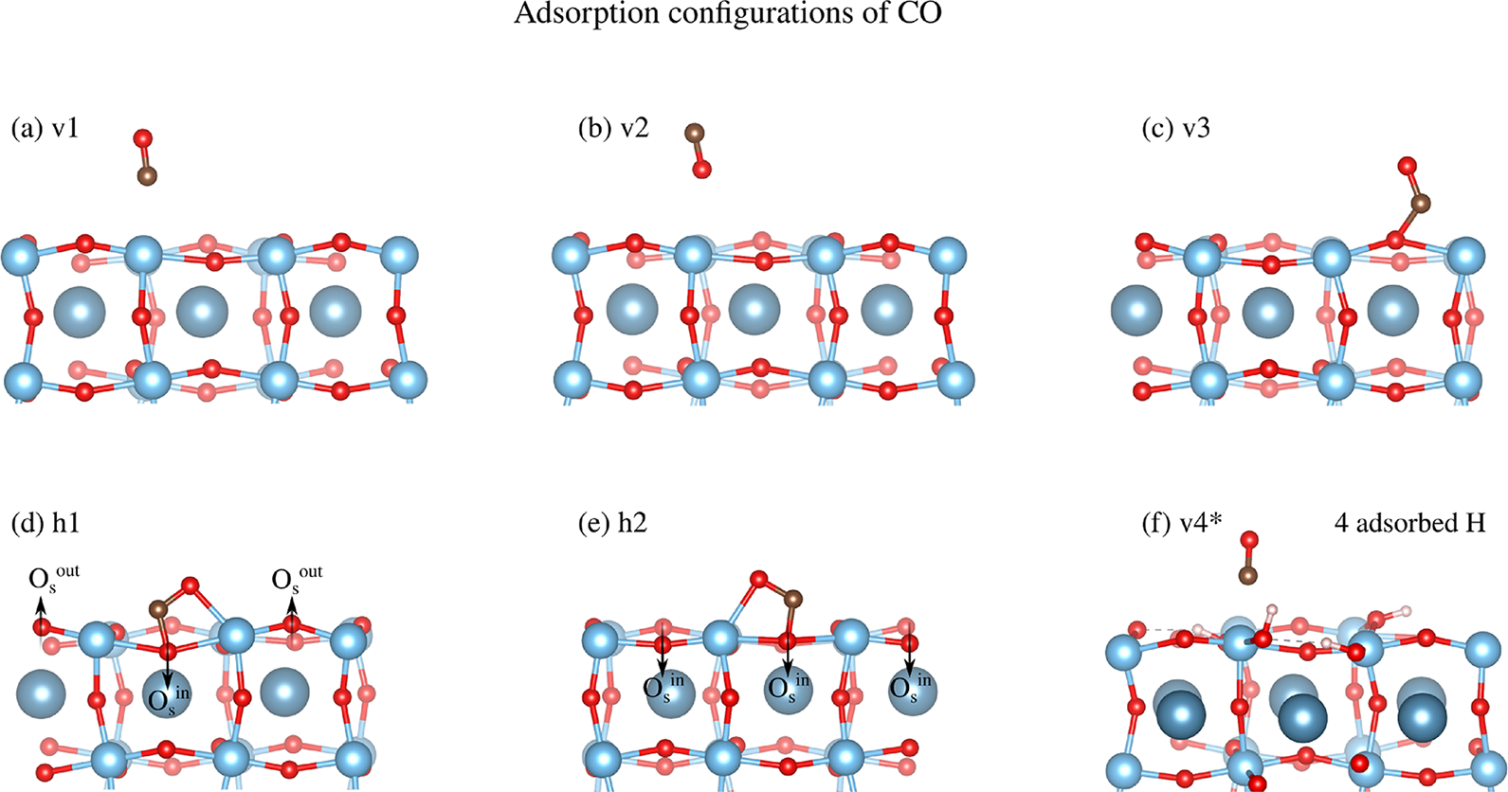
Adsorption configuration of CO on TiO_2_-terminated CaTiO_3_(100): (a) v1; (b) v2; (c) v3; (d) h1; (e) h2; v4*. Activated configurations are labeled with asterisks.

**Table 2:** Adsorption Energy, Bond Lengths, and CO Angle of the Various Adsorption Configurations^a^

conf.	*E*_ads_(eV)	C–O (Å)	Ti–C (Å)	Ti–O (Å)	C–O_s_ (Å)
v1	–0.49	1.14	2.37	3.49	
v2	–0.19	1.14	3.69	2.61	
v3	–0.23	1.21	2.11	3.27	1.38
h1	–0.61	1.25	2.15	2.16	1.36
h2	–0.09	1.26	2.23	2.23	1.35
v4*	–0.52	1.16	2.17	3.32	

a Activated configurations are labeled with asterisks.

### 3.4 Photoreduction of CO to CH_3_OH

On the basis of the CO adsorption mechanisms previously discussed, the v1, v3, v4*, and h1 configurations were selected as initial states for the investigation of the CH_3_OH formation. Figures 5 and 6 report on the overall reaction mechanism described by four consecutive reduction reactions as a function of the reaction coordinate, along with the energetics of the TS, the adsorption state (AS), and the FS. All energies are reported with respect to the 0 eV reference state relative to that of CO in the gas phase, far away from the surface. Adsorption processes are assumed to be barrierless; thus, the desorption activation energy equals the binding energy of AS. Moreover, deviations in *E*_ads_ up to ∼0.3 eV were noticed depending on the number and relative position of adsorbed H atoms in the atomic system. For instance, if one H atom is added on the surface near the O of CO, as in CO-h1.1 of Figure 5a, the *E*_ads_ of CO becomes less negative (−0.54 eV) with respect to −0.61 eV of the CO-h1 state shown in Figure 4d (see also Table 2). Instead, if the H atom is moved close to the C of CO, as in CO-h1.2, *E*_ads_ further decreases by ∼0.08 eV with respect to the CO-h1.1 case.

**Figure 5:**
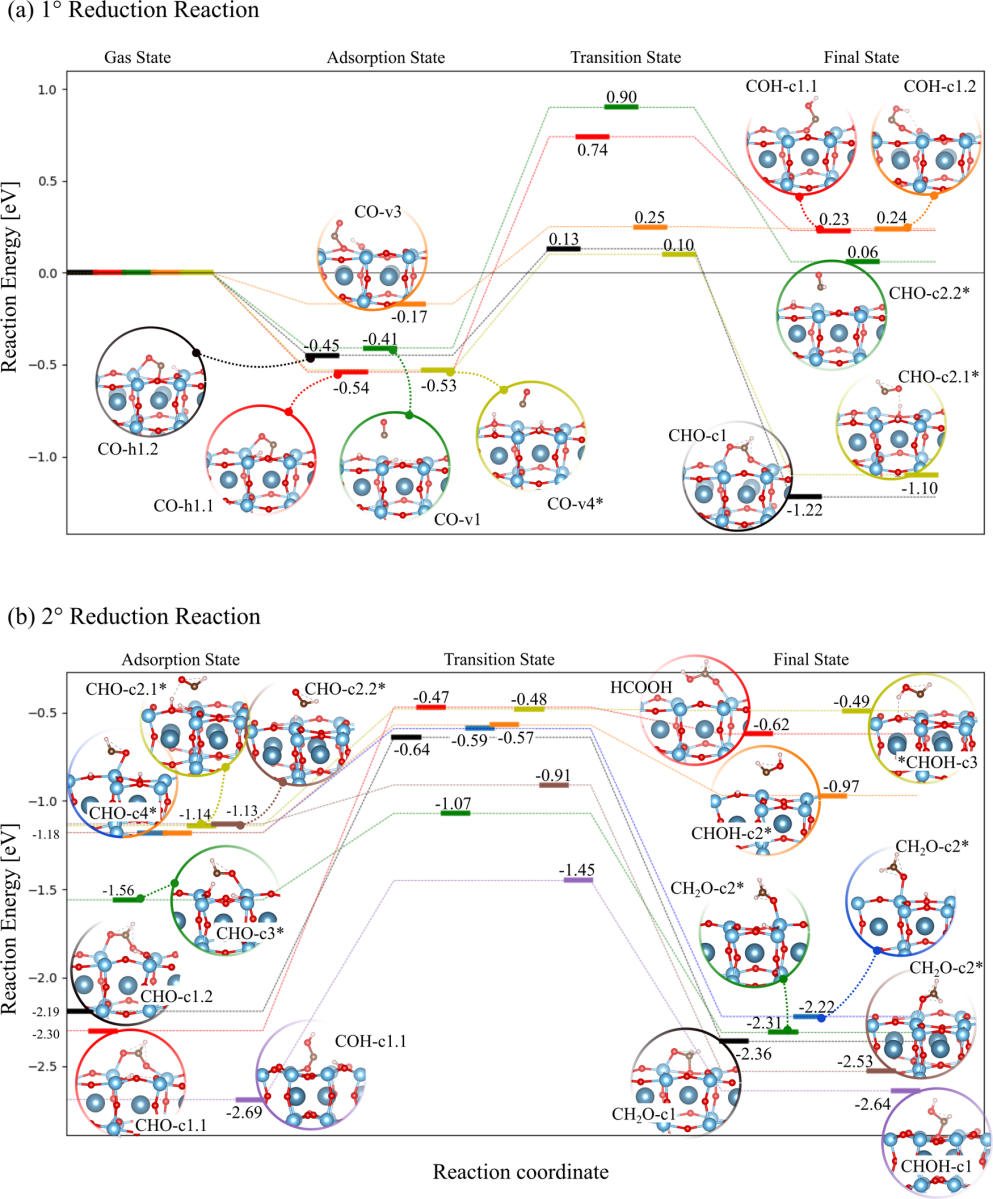
Reaction energy diagram of CO photoreduction to CH_3_OH: (a) first and (b) second reduction reactions.

**Figure 6:**
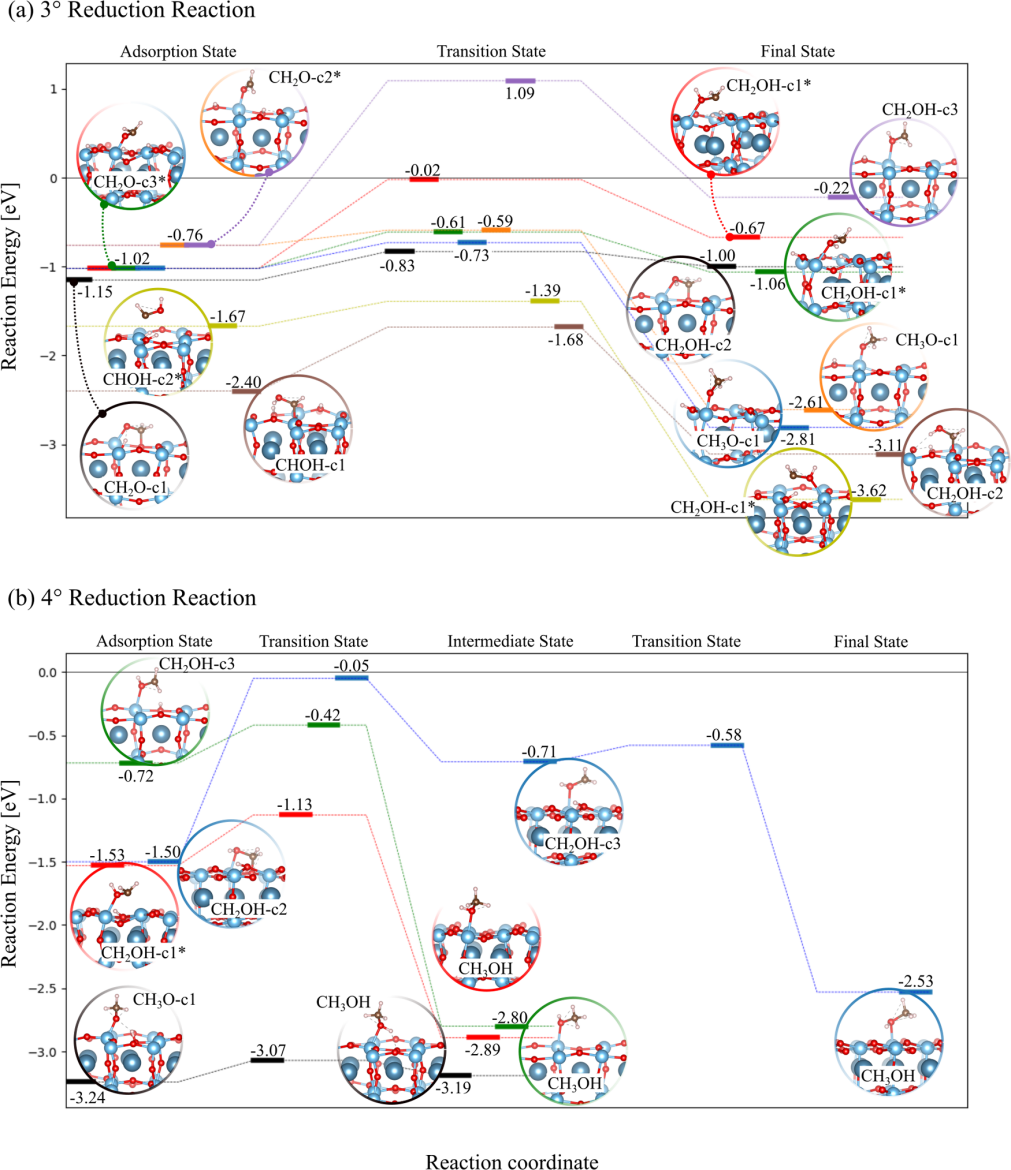
Reaction energy diagram of CO photoreduction to CH_3_OH: (a) third and (b) fourth reduction reactions.

The first CO reduction step (Figure 5a) can lead to adsorbed formyl (CHO) or COH intermediates on the crystal surface. The adsorbed CO molecule (i.e., in AS) can either desorb and return to the gas phase or undergo reduction to the FS. The outcome of this scenario is driven by the energetic competition between these two events, as will be discussed in the following. For the COH formation, the reduction of O of CO-h1.1 (red path) requires 1.28 eV since the Ti–O bond must be broken before hydrogenation could occur. As the energy barrier is 1 order of magnitude higher than that of the competing desorption (0.54 eV), we can assume that most CO molecules will likely desorb before being reduced to CO-c1.1. The energy barrier lowers to 0.42 eV to give COH-c1.2 from CO-v3 (orange path) as the unsaturated O atom is more prone to be hydrogenated. The order of magnitude of the two competitive events, reduction and desorption of CO-v3, is almost comparable. However, most as-formed COH-c1.2 molecules will preferably decompose and go back to the initial AS since the energetics of the reverse reaction (inexpensive *E*_b_ of 0.01 eV) tends to drive the reduction backward. Very interestingly, the reduction of the O of CO-v2 was not observed: if we progressively move the proton to the O of CO-v2 to simulate the FS of the COH, followed by geometry optimization, the CO–H molecule will spontaneously decompose, moving to the AS with the proton going back to the adsorbent O_s_. For the CHO formation, three reaction pathways were investigated. First, the reduction on C of CO-h1.2 (black path) is promoted by two factors: (i) its low *E*_b_ value of 0.58 eV is commensurate to the desorption activation energy of 0.45 eV and (ii) the reaction will likely proceed forward since the as-formed CHO-c1 molecules are energetically more stable than the AS (1.35 eV are required to go backward). Second, the lower chemical reactivity of the physically adsorbed CO-v1 hinders the occurrence of the reduction on C (green path), having a prohibitive *E*_b_ of 1.31 eV. Third, charge transfer in CO-v4* supports the first reduction step (yellow path) as *E*_b_ halves to 0.63 eV with respect to the equivalent inactivated configuration (CO-v1), being now comparable to the activation energy of the competitive desorption event (−0.53 eV). The formation of CHO-c2.1* is also promoted by the higher stability of the FS over the AS by ∼0.57 eV. A more complex situation was observed for the reduction of the CO-v3 on C (not shown in Figure 5a). The mechanism can be described by two consecutive elementary steps, namely, (i) the v3-to-v1 conversion, in which *E*_b_ exceeds 1 eV, followed by (ii) the hydrogenation that advances through the black path leading to CHO-c1. From the above discussion, CHO-c1 and CHO-c2.1* could be the most likely intermediates of the early CO reduction as a result of the combination of favored activation energies and highly chemically stable FS. The former intermediate preserves the initial CO-h1 geometric structure, although the C atom no longer interacts with the adjacent Ti (3.12 Å long distance), and the O_s_ dislocates upward. Instead, the latter experiences charge transfer and locates on top of Ti with an intermolecular distance of 2.24 Å.

Figure 5b shows the second reduction step of CHO leading to formaldehyde (CH_2_O) and CHOH, both anchored on the crystal surface. For the sake of completeness, the reduction on C of COH-c1.1 was also accounted for, even though the production of such an intermediate in the early reduction stage is likely prevented by the prohibitive energetic cost. It is worth noting that the CHO-c1.1 reduction on O has led to HCOOH (red path) instead of CHOH, which induces the formation of an O_s_ defect. Indeed, the CHO fragment withdraws the underneath O_s_ involved in the C–O_s_ bond that now becomes part of the molecule. However, the process requires 1.83 eV to give the formic acid. The molecule in CHO-c1.1 and CHO-c1.2 has the same geometric structure, but different relative H positions were considered, which consequently give distinct *E*_ads_ of −2.30 and −2.19 eV, respectively. The CH_2_O formation is first addressed as follows. Among all the CHO configurations, CHO-c1.1 is the lowest-energy state with *E*_ads_ of ∼−2.3 eV. Nevertheless, it can hardly be reduced on C (black path), as 1.55 eV is required to overcome the reaction barrier. To facilitate the hydrogenation, the O_s_ involved in the C–O_s_ rearrange moving downward, which could explain the high energy cost.

In all remaining CHO configurations, i.e., CHO-c2*, CHO-c3*, and CHO-c4*, charge transfer occurs. *CHO-c3 is the most energetically stable activated structure with *E*_ads_ of −1.56 eV in which the molecule interacts with a Ti–Ti pair via both its C and its O forming Ti–C and Ti–O bonds of 2.33 and 2.23 Å long, respectively. In the *E*_ads_ range of −1.1 ÷ 1.2 eV, the CHO-c4* and CHO-c2* states are found. In CHO-c4*, the C–O bond locates on top of a single Ti atom, forming a bridging bond with Ti–C and Ti–O distances of 2.09 and 2.17 Å, respectively, while the surface-molecule interaction in CHO-c2* is established only by the Ti–C bond (2.24 Å long). The CHO-c2.1* and CHO-c2.2* states share the same geometrical structure, but they have different molecular orientations: in the latter configuration, the O points upward far away from the surface, while in the former it approaches the near adsorbed H with which it could react and give CHOH. Note that two adsorbed H atoms are required to stabilize CHO-c3* and CHO-c4*, while only one H is sufficient to account for charge transfer in CHO-c2*. The lowest energy cost to produce CH_2_O amounts to 0.22 eV associated with the reduction on C of CHO-c2.2* (brown path), which increases to 0.49 and 0.59 eV for CHO-c3* (green path) and CHO-c4* (blue path), respectively. All three initial states lead to the same CH_2_O-c2 structure, whose FS locates ∼1 eV on average below the AS. As a result, the energetics will likely tend to drive the reduction step forward when activated CHO configurations are involved, thus strongly supporting the CH_2_O production. For CHOH formation, three reduction steps were identified and investigated. A similar energy cost was computed to reduce CHO-c4* (orange path) and CHO-c2.1* (yellow path) on the O atom, which amounts to 0.61 and 0.66 eV, respectively. However, the *E*_b_ of the reverse reaction relative to the yellow path is inexpensive (0.01 eV), which supports most of the as-formed CHOH-c3* to easily dehydrogenate on O, going back to the AS, while the orange path is more prone to proceed forward and to give the more energetically stable CHOH-c2*. The third pathway accounts for the reduction of C of COH-c1.1, the lowest-energy structure (*E*_ads_ of −2.69 eV) among all the investigated configurations of Figure 5b in which a strong bridging bond is formed between C and the Ti–O_s_ pair. The Ti–C and C–O_s_ bonds are 2.18 and 1.27 Å long, respectively. The *E*_b_ of this process leading to CHOH-c1 is 1.24 eV. With the information gained in Figure 5b, we can finally assert that CH_2_O-c2 can easily be formed from activated CHO configurations, while only CHO-c4* could likely give CHOH-c2*, though the reduction can also proceed backward.

Figure 6a shows the third reduction stage that leads to adsorbed methoxy (CH_3_O) and hydroxymethyl (CH_2_OH) intermediates. As a general trend, CHOH configurations are more energetically stable than CH_2_O ones. CHOH-c1 has the highest *E*_ads_ of −2.40 eV, which preserves the bond arrangement of COH-c1.1 (see Figure 5b), although C–O_s_ now elongates to 1.48 Å with respect to 1.27 Å in COH-c1.1, meaning that the double bond character is completely lost. The *E*_ads_ increases to −1.67 eV for CHOH-c2*. The surface-molecule interaction is only dictated by the Ti–C bond that is 2.10 Å long, slightly shorter than the equivalent bond (2.24 Å long) in CHO-c2*. Both CHOH structures undergo reduction on C, leading to CH_2_OH, whose energetics are promoted for CHOH-c2* (yellow path), demanding only 0.28 eV with respect to 0.72 eV for CHOH-c1 (brown path). The conversion to the FS is further enhanced by its higher chemical stability with respect to AS by around 0.5 ÷ 1.0 eV. Three CH_2_O configurations were identified, among which CH_2_O-c2* and CH_2_O-c3* are two activated states. CH_2_O-c1 has a higher *E*_ads_ of −1.15 eV in which the molecule forms C–O_s_ and Ti–O bonds having lengths of 1.45 and 1.95 Å, respectively. The CH_2_O-c3* structure with an *E*_ads_ of −1.02 eV preserves the same bond arrangement in CHO-c3*: the C–O bond still interacts with the Ti–Ti pair, but the Ti–O distance now reduces to 1.83 Å in comparison to 2.23 Å of CHO-c3*. The lowest *E*_ads_ of −0.76 eV were computed for CH_2_O-c2*, whose surface interaction is regulated by the Ti–O bond. It is worth noting that the same equivalent inactivated structure was also achieved even on the H-unreduced CaTiO_3_ surface, whose surface-molecule distance amounts to 2.22 Å. However, when two adsorbed H atoms are also considered, charge transfer occurs, leading to CH_2_O-c2* with the Ti–O bond that shortens to 2.17 Å. In this work, only the activated configuration was considered. Flattened energy profiles characterized by *E*_b_ of 0.32 and 0.41 eV relative to the reduction on the O of CH_2_O-c1 (black path) and CH_2_O-c3* (green path), respectively, can promote CH_2_OH formation. Besides, the FS and AS are almost located on the same energy level, which may induce a chemical equilibrium. The red path also accounts for the reduction of O of CH_2_O-c3* as the green one, but the proton involved in the hydrogenation process comes from a different initial position. However, *E*_b_ increases to 1.00 eV, which provides a simple and clear example of the expected *E*_b_ dependence on the proton position. Aside from the lowest *E*_ads_, the most expensive energetics is attributed to the purple path relative to CH_2_O-c2 as the computed *E*_b_ is 1.85 eV. The CH_3_O formation is encouraged by affordable activation energies that do not overcome ∼0.3 eV, as evidenced by the blue and orange paths relative to CH_2_O-c3* and CH_2_O-c2*. Moreover, its production is further enhanced over the CH_2_OH formation because the FS are more energetically stable than the AS by around 2 eV, thus supporting the reduction step to proceed forward. The reduction in C of CH_2_O-c1 was also investigated (not shown in Figure 6a). The overall mechanism can be described by two consecutive elementary steps, namely, (i) the c1-to-c2 conversion followed by (ii) the catalytic hydrogenation in c2. Step (i) is fundamental to break the C–O_s_ bond and let the C atom be hydrogenated. Only 0.53 eV is required to reach the CH_2_O-c2* intermediate state, while step ii follows the orange path. According to the previous results, the third reduction stage can easily give CH_3_O as the main intermediate since the corresponding energy profiles are characterized by affordable activation energies not higher than 0.3 eV. Besides, only the reduction of CHOH-c2* could likely lead to a substantial production of CH_2_OH molecules due to the combination of a low *E*_b_ of 0.28 eV and a highly energetically stable FS, which discourages the reduction reaction from going backward.

Pathways of the last reduction step leading to the final CH_3_OH byproducts are summarized in Figure 6b. CH_3_O-c1 is the most stable intermediate with an *E*_ads_ of −3.24 eV: the C atom is finally saturated by the O and three H atoms, while the surface interaction is established by the Ti–O bond that is 1.81 Å long. Thus, only the O atom in CH_3_O-c1 can be hydrogenated. The reaction process proceeds through the black path characterized by a flattened energy profile as the *E*_b_ values of the forward and backward reaction are smaller and equal to 0.17 and 0.12 eV, respectively. As a result, a chemical equilibrium could be reached.

Three possible configurations of CH_2_OH were identified. CH_2_OH-c1* and CH_2_OH-c2 have higher chemical stability with *E*_ads_ values of −1.53 and 1.50 eV, respectively. Here, the molecule interacts with the surface via the entire C–O bond: in CH_2_OH-c1*, the Ti–Ti pair is implicated in the Ti–C and Ti–O bond formation (2.25 and 2.22 Å long, respectively); while, Ti–C is substituted by the C–O_s_ with a bond length of 1.40 Å in CH_2_OH-c2. Adsorption in CH_2_OH-c3 with an *E*_ads_ of −0.72 eV is established by the Ti–O bond that is 2.27 Å long. The C reduction of CH_2_OH-c1* (red path) and CH_2_OH-c3* (green path) requires a favorable *E*_b_ of 0.40 and 0.30 eV, respectively, which gives CH_3_OH whose FS locates 2 eV lower than the AS. While the mechanism for CH_2_OH-c2 (blue path) follows two consecutive elementary steps, analogously to the C reduction of CH_2_O-c1. First, the C–O_s_ bond must be broken to accommodate the H atom on C, leading to the CH_2_OH-c3* intermediate state by providing 1.45 eV. Second, catalytic hydrogenation can proceed through the green path. Once produced, the CH_3_OH molecule is still located on the surface, adsorbed on top of a Ti atom via the Ti–O bond with a length of 2.16 Å. The *E*_ads_ is −1.01 eV, meaning that robust adsorption occurs.

In summary, the first reduction stage is the rate-limiting step of the overall mechanism, since the corresponding energy profiles are typically distinguished by higher energetics. The COH intermediate yield is crucially inhibited by the expensive energy barriers of the CO reduction process or by the poor chemical stability of COH, which easily undergoes dehydrogenation. Instead, CHO could be the main intermediate as the energy cost can decrease to 0.6 eV. The second stage of the CO photoreduction mechanism likely follows the C reduction route leading to CH_2_O, but low-stability activated CHO configurations must be implied to drastically decrease *E*_b_ up to 0.22 eV. One possible investigated pathway could give CHOH from CHO, as it requires 0.61 eV, although the reverse reaction is also promoted with the same probability. The catalytic hydrogenation on C becomes even more favorable in the last two stages, i.e., third and fourth reduction steps, and CH_2_O and CH_3_O will constitute the substantial part of intermediates. On the contrary, the reduction of O could reach the chemical equilibrium since the activation energies of the forward and backward reactions are comparable. Even though CH_3_OH can be finally formed, its production yield could be affected by its dehydrogenation on O (*E*_b_ 0.12 eV), which competes with the CH_3_OH desorption process that requires 1.01 eV (if barrierless). This could explain why it has not been detected yet in CO_2_ photoreduction experiments.^36,37^

### 3.5 Photoreduction of CO to CH_4_

Contrary to the CH_3_OH formation mechanism, the CO photoreduction to CH_4_ includes one additional elementary step dedicated to the removal of the O from adsorbed CO-derived intermediates, which leads to the production of early hydrocarbon fragments (CH_*x*_, with *x* = 0, 1, 2, 3) on the crystal surface. Then, such hydrocarbons undergo subsequent catalytic hydrogenation steps, which eventually produce CH_4_. In this section, we (i) determine at which stage of the entire mechanism the C–O bond breaking could likely occur and, consequently, (ii) identify the CO-derived fragments that are more prone to lose their O along with the as-formed early hydrocarbons. As an example, the decomposition of CHOH, namely, the CO-derived fragment, might produce CH as an early hydrocarbon.

Most of the intermediates identified in the previous section have been selected as possible candidates for the decomposition study, as shown in Figure 7. Those fragments that do not bear an H on their O atom (CH_*x*_O species with *x* = 1, 2) are grouped in panel (b) of Figure 7 and distinguished by their higher decomposition energy barriers (not lower than 1.73 eV), while the CH_*x*_OH species are collected in panel (a) of Figure 7, with *x* = 0, 1, 2. Although the reduction of CO to COH is inhibited, as already discussed, we have evenly accounted for the simplest scenario represented by the COH decomposition (red path) that gives the elemental C atom and OH. However, the high *E*_b_ value of 1.33 eV for this process is an indication of the low proclivity of the molecule to dissociate. In addition, even though the FS could be reached, the high reactivity of the dissociated elemental C atom would favor its recombination with OH, especially for fast diffusion of the various species. The formation of the simplest hydrocarbon fragment, CH, from the CHOH and CHO intermediates was studied. The CHOH-c1 decomposition (orange path) has the lowest *E*_b_ of 0.62 eV among all of the investigated pathways. Its occurrence is further enhanced by the higher chemical stability of the initial state (*E*_ads_ of −2.07 eV) with respect to the other equivalent configuration, namely CHOH-c2*, which is located at −1.67 eV and whose dissociation (purple path) has an expensive *E*_b_ of 1.37 eV. Moreover, going backward along the orange path is discouraged, since the energy cost to overcome the reverse reaction barrier is higher by around 0.7 eV. The as-formed HC species in the FS forms a strong bridging bond with the O_s_–Ti–O_s_ tuple. The *E*_b_ drastically increases to 1.77 eV when CHO-c1 decomposition is considered. Dissociation of CH_2_O or CH_2_OH can lead to the hydrocarbon fragment CH_2_. It is worth noting that CH_2_OH-c2 and CH_2_O-c1 share a similar geometric structure in which the O–H bond is present only in the latter configuration, as also observed for the equivalent CH_2_OH-c1* and CH_2_O-c3* states. The decomposition CH_2_OH-c2 (yellow path) has the second lowest *E*_b_ of 0.97 eV, while the remaining pathways are characterized by *E*_b_ that easily overcome 1.73 eV. The formed CH_2_ could then rebind to OH, following the yellow path to give the CH_2_OH-c2 molecule in AS, but 1.21 eV is required to overcome the barrier. CH_3_ production was also investigated from CH_3_OH dissociation (not shown in Figure 7). The mechanism can be described as a two-step reaction. The first step, that is, the breakage of the OC bond, is the rate-limiting step with an *E*_ads_ of 1.46 eV that generates the metile group formed in the gas phase. In the second step, CH_3_ barrierless adsorption occurs.

**Figure 7:**
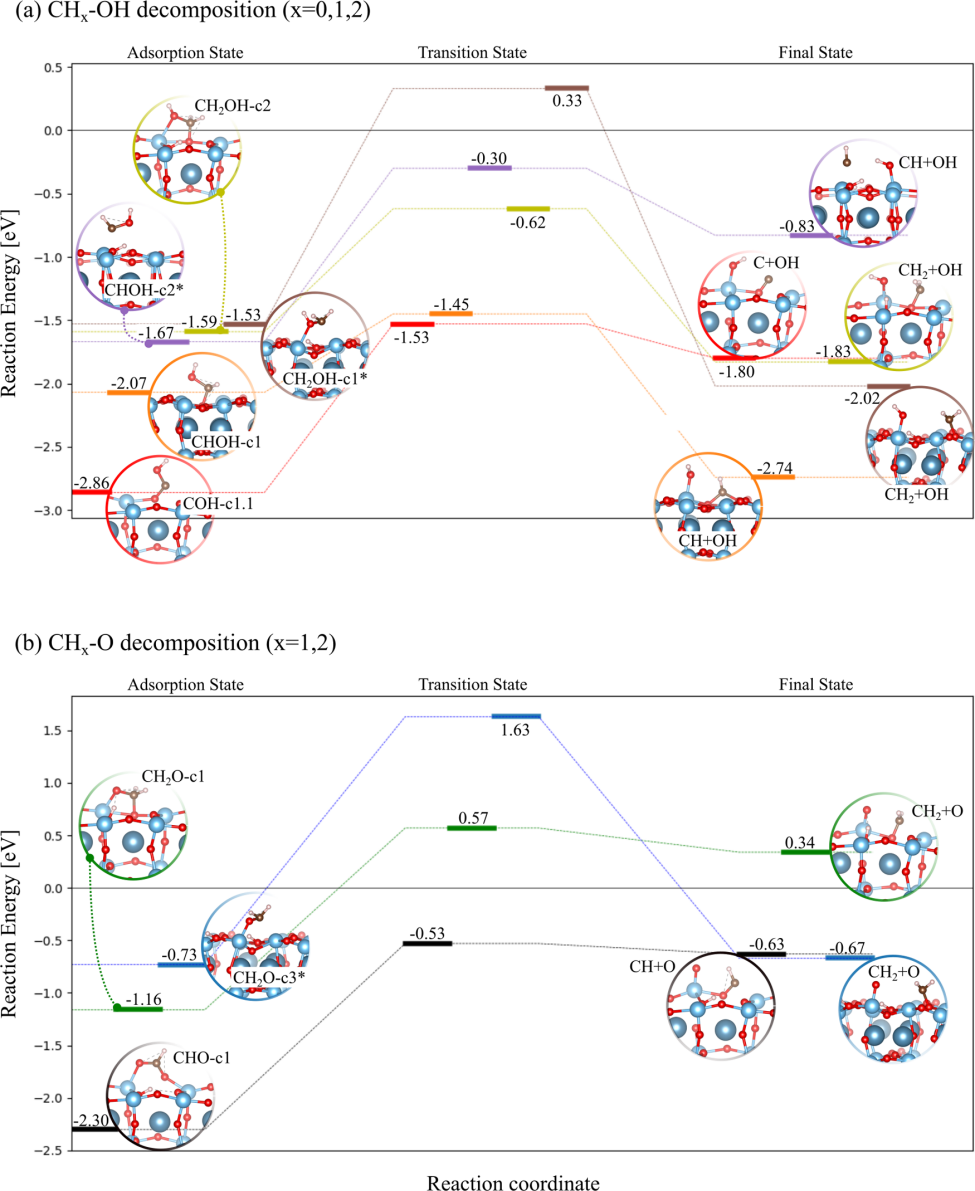
Reaction energy diagram of CO photoreduction to CH_4_: (a) CH_*x*_–OH decomposition (*x* = 0, 1, 2), (b) CH_*x*_-O decomposition (*x* = 0, 1, 2).

On the basis of the above discussion, CH can likely be the only accessible product of the decomposition stage. Afterward, three consecutive hydrogenation steps on CH could lead to the final CH_4_ byproducts, thus following part of the well-known “carbene pathway.”^2^ The complete mechanism of CH photoreduction to CH_4_ is reported in Figure 8. Four adsorption configurations ‘c’ can be typically identified for every reduction step since CH_*x*_ species (*x* = 1, 2, 3) can locate (i) between two O_s_ in a bridge-like disposition (c1, see black paths), (ii) on top of O_s_^up^ (c2, see green paths), (iii) on top of O_s_^down^ (c3, see red paths) or (iv) on top of Ti (c4, see blue paths). Higher reactivity of hydrocarbon fragments to hydrogenation is observed for activated configurations and for highly unsaturated compounds such as CH. Moreover, the overall energy profiles will tend to drive the reactions forward as the reverse reactions *E*_b_ are prohibitive. The most energetically favorable mechanism follows the overall blue path, which involves only activated c4* configurations and whose energy profile has increasing *E*_b_ by around 0.1 eV for every hydrogenation step, thus from 0.07 eV for the CH reduction to 0.22 eV for the second hydrogenation, which finally extends to 0.36 eV to give CH_4_. However, activated c4* configurations are characterized by higher *E*_ads_ values with respect to inactivated ones, apart from CH_3_-c4*, which has the lowest *E*_ads_ of −1.55 eV among all of the CH_3_ structures of the third reduction stage. The CH_*x*_ species in c4* (*x* = 1, 2, 3) lie on top of Ti at a distance within the 2.04 ÷ 2.11 Å range. The CH_4_ formation route can also easily proceed via the green path as the first two hydrogenation steps occur on activated configurations, namely CH-c2* and CH_2_-c2*, requiring only 0.04 and 0.14 eV, respectively. In CH-c2*, C forms a bridging bond with the Ti–O_s_^up^ pair with Ti–C and C–O_s_^up^ bond lengths of 2.12 and 1.34 Å, while the molecule in CH_2_-c2* on top of the O_s_^up^ (C–O_s_^up^ bond length of 1.35 Å) tilts by 56.4°, being the tilting angle defined as the angle between the C–O_s_^up^ bond and the surface plane. The last (third) hydrogenation step on inactivated CH_3_-c2 was observed as a two-step reaction. The CH_3_-c2 preserves the CH_2_–c2* geometric structure with a C–O_s_^up^ bond that is 1.43 Å long and a tilting angle of 65.4°. In order to accommodate the fourth H on its molecular structure, chemisorbed CH_3_ must first detach from the surface and move to the gas phase, thus assuming the characteristic trigonal planar molecular geometry (see the Intermediate State in Figure 8c). However, this process needs to overcome an energy barrier of 2.02 eV. Then, the radical metile in the gas phase requires only 0.03 eV to withdraw the proton from the surface and give CH_4_. It is evident that CH photoreduction along the green path can easily proceed until the second hydrogenation step. The energy profile of the CH photoreduction becomes more expensive when the overall red path is accounted for. Although the first reduction stage on CH-c3 to CH_2_-c3 has a low *E*_b_ of 0.20 eV, the second hydrogenation results in less convenient (0.68 eV). In both CH-c3 and CH_2_-c3 configurations, the C interacts with the Ti–O_s_^down^ pair with Ti–C and C–O_s_^down^ bond lengths of 2.04 and 1.37 Å for the former, or 2.15 and 1.44 Å for the latter. The reduction of CH_3_-c3 follows the same mechanism observed for CH_3_-c2, in which a two-step reaction was essential to describe the CH_4_ formation. The first event again needs a prohibitive energy cost, but now reduces to 1.66 eV. The surface-molecule distance in CH_3_-c3 is 1.44 Å. Due to the strong binding with the two O_s_ in CH-c1, as also reflected by its lowest *E*_ads_ of −4.24 eV, the CH reduction exhibits a higher *E*_b_ of 0.68 eV. The as-formed CH_2_-c1 preserves the CH-c1 geometrical structure in which the molecule is located between two O_s_. However, hydrogenation on CH_2_-c1 was not observed since C is fully saturated by the two O_s_ and two H atoms. Thus, the C–O_s_ bond must be broken first to let the proton attach to the CH_2_ fragment. As a result, the c1-type configurations involved in the mechanism are not optimal for the CH photoreduction process. Finally, the as-formed CH_4_ physisorbs with *E*_ads_ of −0.22 eV at a surface-molecule distance not lower than ∼3.2 Å due to its nonpolar nature that prevents a strong adsorption on the surface. This could also explain why it has been experimentally detected,^36,37^ as opposed to CH_3_OH, which has lower *E*_ads_ of −0.93 eV and a good proclivity to dehydrogenate on its O atom when adsorbed.

**Figure 8:**
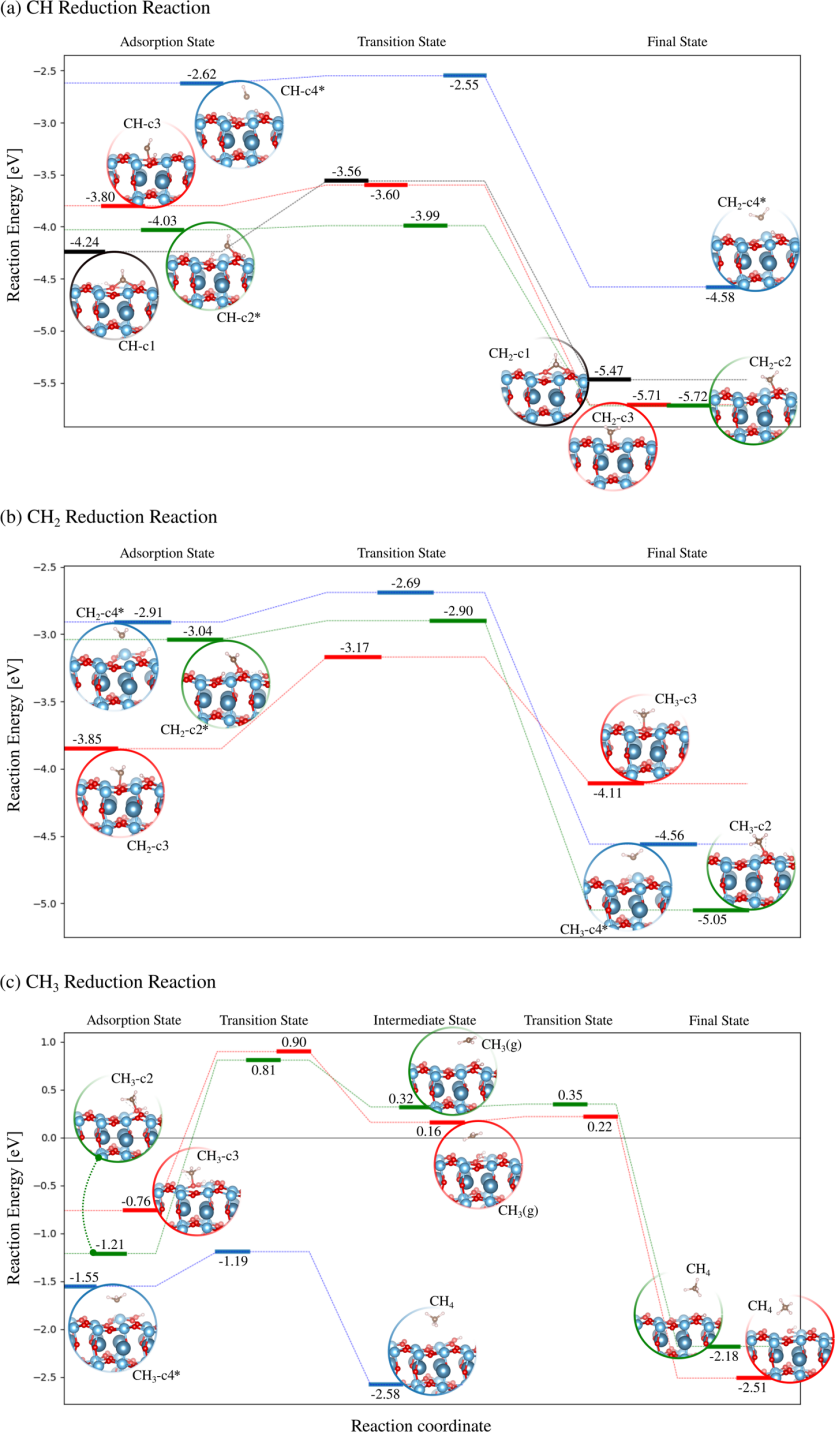
Reaction energy diagram of CH photoreduction to CH_4_: (a) first, (b) second, and (c) third reduction reactions.

## 4 Conclusions

In summary, we investigated the complete reaction mechanism of the CO_2_ photoreduction on TiO_2_-terminated CaTiO_3_(100), including the CO_2_ adsorption and activation processes, and the generation of value-added byproducts such as HCOOH, CO, CH_3_OH, and CH_4_. When the CO_2_ molecule approaches the crystal surface, it first physisorbs, preserving its linear structure, then moves to a more stable AS in which CO_2_ arranges in the BC configuration, having the lowest *E*_ads_ of −1.27 eV. The activation of CO_2_ was simulated by introducing adsorbed H atoms. We found three activated configurations with higher *E*_ads_ values as a result of the molecular electronic reorganization that converts the linear molecule to the bent radical anion CO_2_^·–^. Due to its higher chemical reactivity, CO_2_^·–^ can be easily hydrogenated on its C atom, leading to the formation of adsorbed HCOO^–^, while the reduction pathway to COOH was not identified. The second reduction step that generates HCOOH from HCOO^–^ is inhibited by demanding *E*_b_ of 1.59 eV, which could support the experimental evidence that the HCOOH byproduct is typically not detected during the CO_2_ photoreduction using CaTiO_3_ as photocatalyst. Instead, the weaker internal bonding of CO_2_^·–^ promotes its decomposition to give CO as reflected by *E*_b_, which can lower to 0.16 eV. As CO can probably be one of the most abundant species of the photoreduction process, in agreement with experimental observations, it can actively participate in the photoreduction mechanism to give CH_3_OH and CH_4_. The CO adsorption and activation processes on CaTiO_3_(100) are also explored. For chemisorbed configurations, the lowest *E*_ads_ of −0.61 eV is found in which the entire molecule bonds to the underneath surface. Charge transfer to CO only occurs when the molecule vertically locates on top of the Ti atom with its C atom. The conversion of CO to CH_3_OH is described by four consecutive reduction reactions. The first elementary step is the rate-limiting step whose *E*_b_ cannot be lower than 0.6 eV due to the poor CO chemical reactivity. Despite the fact that CO activation has significant catalytic action, the lowest *E*_b_ value is found for the inactivated configuration. The overall mechanism preferably proceeds via hydrogenation on the C atom rather than on the O, so that the predominant intermediates are the CH_*x*_O (*x* = 1, 2, 3) species at this photoreduction stage. Moreover, the reaction occurrence is significantly enhanced in the third and fourth reduction steps, as they are characterized by lower *E*_b_. Even though CH_3_OH can be finally formed on the crystal surface, it will likely undergo dehydrogenation on its O, giving CH_3_O + H before desorption could occur, which could explain why it has not been detected in CO_2_ photoreduction experiments. The CO-to-CH_4_ conversion mechanism includes one elementary step dedicated to the removal of the O from adsorbed CO-derived fragments, which produces early hydrocarbons (CH_*x*_, with *x* = 1, 2, 3) on the crystal surface. The results reveal that the most probable reaction pathway gives CH as an early hydrocarbon through CHOH decomposition (*E*_b_ = 0.62 eV). For CH species, three consecutive reduction steps are required to form CH_4_ and are favored by the combination of charge transfer effect and higher reactivity to hydrogenation of hydrocarbon fragments on the surface. CH_4_ has been experimentally detected as it can easily desorb due to its nonpolar nature (*E*_ads_ = −0.22 eV), compared to CH_3_OH that has a lower *E*_ads_ of −0.93 eV and good proclivity to decompose to CH_3_O + H on CaTiO_3_(100).

## Supplementary Material



## Notes

Convergence tests, role of the Hubbard *U* parameter, DOS and PDOS plots of the supercell slabs, input file template and geometry of all studied stable and transition states (PDF)
