# Supplementary material for: CO2 Adsorption and Photocatalytic Reduction Mechanisms on TiO2‑Terminated CaTiO3(100): A Density Functional Theory Study
Source: J Phys Chem C Nanomater Interfaces. 2025 Oct 28;129(44):19756–71. doi: 10.1021/acs.jpcc.5c04650 (PMC12599115; doi:10.1021/acs.jpcc.5c04650)
Supplement: Supplementary file 1 [file jp5c04650_si_001.pdf]

Supplementary Information

CO_2 Adsorption and Photocatalytic Reduction Mechanisms on TiO_2-Terminated $CaTiO_3$(100): a DFT Study

Onofrio Tau,[1,*] Giacomo Giorgi,[2,3,4,5,*] and Riccardo Rurali[1,*]

[1] Institut de Ciència de Materials de Barcelona, ICMAB-CSIC, Campus UAB, 08193 Bellaterra, Spain.

[2] Department of Civil and Environmental Engineering (DICA), Università degli Studi di Perugia, Via G. Duranti 93, 06125, Perugia, Italy.

[3] CNR-SCITEC, I-06123, Perugia, Italy.

[4] CIRIAF - Interuniversity Research Centre, University of Perugia, 06125, Perugia, Italy

[5] Centro S3, CNR-Istituto Nanoscienze, Via G. Campi 213/a, Modena, 41125, Italy

*Corresponding author e-mail: otau@icmab.es, giacomo.giorgi@unipg.it, rrurali@icmab.es

Convergence test of the adsorption energy and lattice parameters

Table S1: Convergence test of the CO_2 adsorption energy relative to the L1, BC and B1 configurations with respect to the number of slab layers, namely, 5 and 7 layers, and the Hubbard parameter *U*. A number of layers greater than 9 would require a huge computational simulation time. The Γ point was sufficient to sample the Brillouin zone. All calculations in this work were thus performed by using 2x2 surface 5-layer slabs with a 15 Å thick vacuum region and *U* = 0 eV, as it turns out sufficient to guarantee unimportant changes (within 0.14 eV) of the calculated energy values and reasonable computational costs with respect to thicker slabs and U = 4.61 eV (computed by the hp.x[1] code of Quantum ESPRESSO).

	E_{ads} (eV)		
	L1	BC	B1
5layers + *U*=0.00eV	-0.43	-1.27	0.09
5layers + *U*=4.61eV	-0.42	-1.41	0.12
7layers + *U*=0.00eV	-0.46	-1.26	0.18
7layers + *U*=4.61eV	-0.44	-1.40	0.10

Table S2: Lattice constants of the $CaTiO_3$ orthorhombic primitive cell as a function of the Hubbard parameter *U*. Calculated *U* = 4.61 eV (computed by the hp.x[1] code of Quantum ESPRESSO) exhibits negligible changes (within less than 1%) in lattice constants with respect to the *U* = 0 eV case.

	a	b	c
U=0.00eV	5.394	5.497	7.679
U=4.61eV	5.412	5.515	7.706

DOS and PDOS of TiO_2-terminated CaTiO3 (100) surface slabs with and without adsorbed H

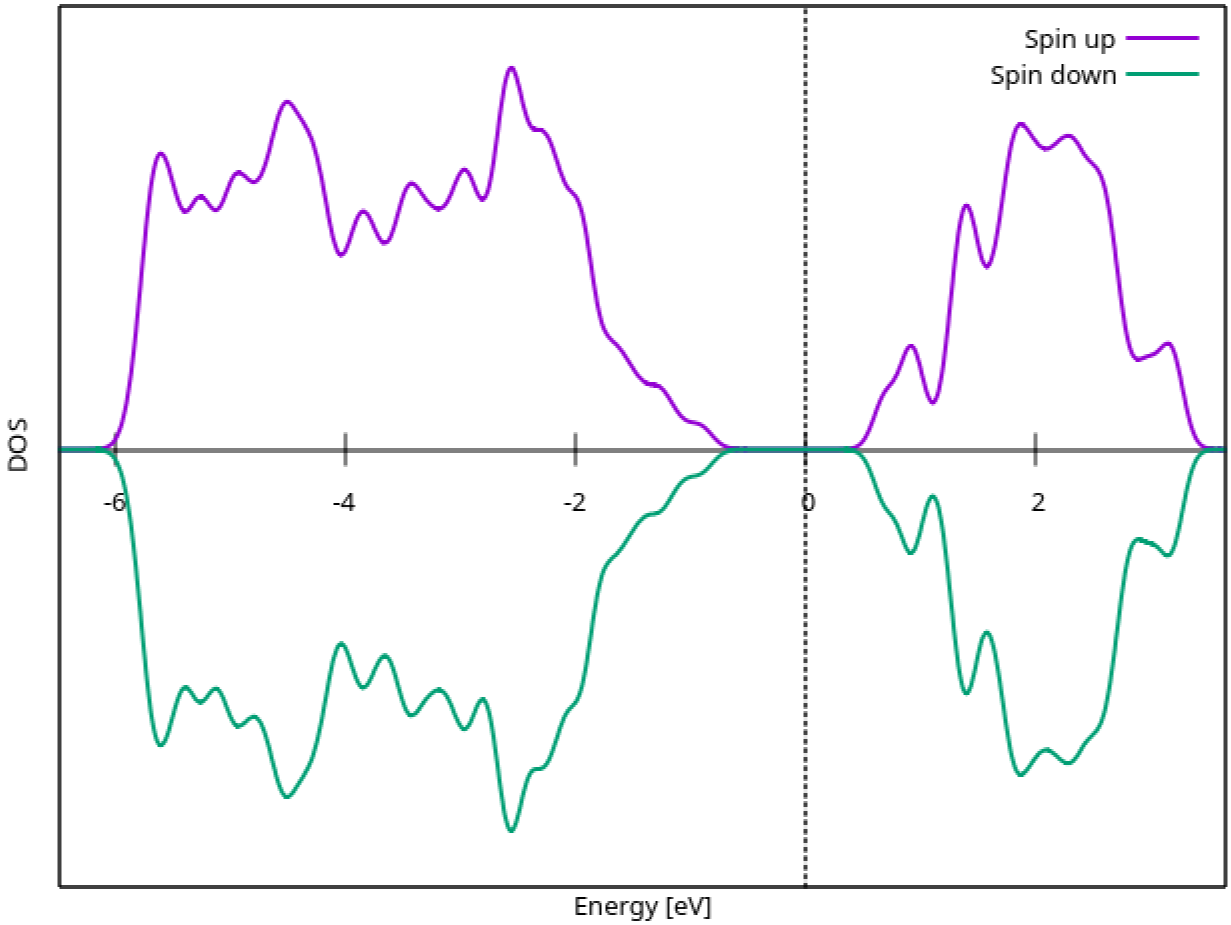

Figure S1. Total spin-up and spin-down electronic density of states (DOS) of the TiO_2-terminated $CaTiO_3$ (100) surface modeled by the non-stoichiometric 5-layer symmetric slab. The Fermi energy (black dashed line) is aligned to 0 eV and lies in the middle of the valence and conduction bands, meaning that no partial charge transfer occurs within the slab.

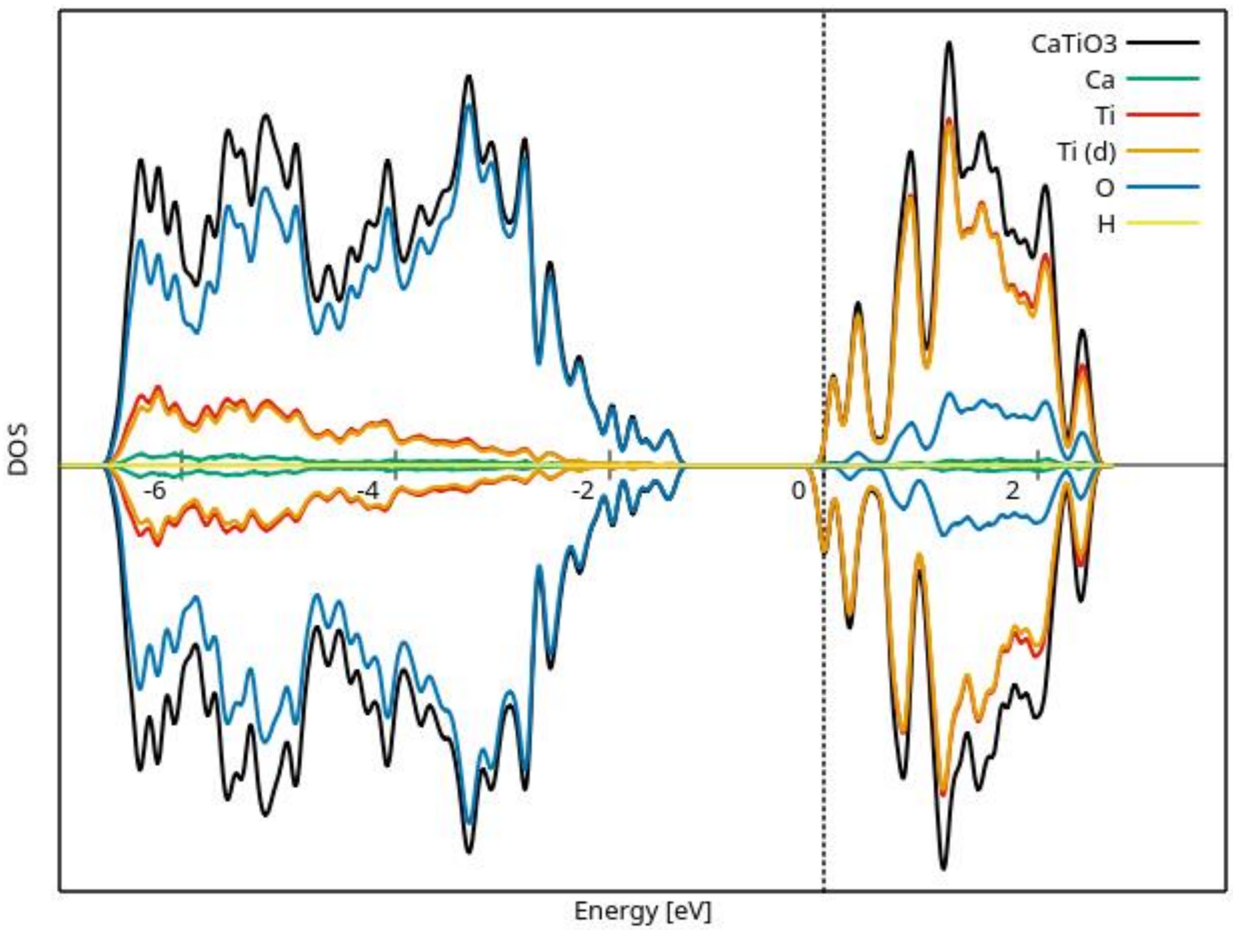

Figure S2. Total spin-up and spin-down electronic projected density of states (PDOS) of individual atoms (including the d electrons of Ti represented separately by the orange line) that constitute the TiO_2-terminated $CaTiO_3$ (100) surface slab with one adsorbed H atom. When one H atom is introduced on top of the pure $CaTiO_3$ surface, the Fermi energy (black dashed line) shifts to the Ti 3d electronic states (orange line) of the conduction band as a result of the electron charge transfer from the adsorbed H to the $CaTiO_3$ surface. In particular, this excess electron homogeneously redistributes over the 3d orbitals of Ti atoms (orange line) as calculated by the Lowdin charge analysis (not shown here).

Example of the PWscf input file template

```
 &CONTROL
   calculation     =  'scf',
   outdir          =  './tmp',
   pseudo_dir      =  './pseudo',
   tprnfor         =  .TRUE.,
 /

 &SYSTEM
   a               =  10.7885351181,
   b               =  10.9949169159,
   c               =  22.9971080000,
   ecutrho         =  450,
   ecutwfc         =  45,
   ibrav           =  8,
   nat             =  108,
   ntyp            =  5,
   nspin           =  2,
   starting_magnetization(2) = 0.5,
   occupations     =  'smearing',
   smearing        =  'mp',
   degauss         =  0.001,
   vdw_corr        =  'DFT-D3',
 /

 &ELECTRONS
   conv_thr        =  1.00000e-07,
   mixing_mode     =  'local-TF',
   mixing_beta     =  0.7,
 /

ATOMIC_SPECIES
  Ca    40.078   Ca.pbe-spn-rrkjus_psl.1.0.0.UPF
  Ti    47.867   Ti.pbe-spn-rrkjus_psl.1.0.0.UPF
   O    15.999    O.pbe-nl-rrkjus_psl.1.0.0.UPF
   C    12.011    C.pbe-n-rrkjus_psl.1.0.0.UPF
   H     1.007    H.pbe-rrkjus.UPF


ATOMIC_POSITIONS angstrom
 ...

K_POINTS gamma
```

Cartesian atomic coordinates (Å) of main equilibrium structures

CO_2-L1

```
107
CO2_L1
Ca          -0.1103452506      5.6961130752      5.9071972736
Ca           2.8026600089      2.9468717895      5.9066821541
Ca           2.8136603564      8.4365171703      5.9153353974
Ca           8.1974879833      2.9488268818      5.9000924627
Ca           8.1931950056      8.4455832211      5.9031764020
Ca           2.6474700000      2.5105950000      1.9196440000    0    0    0
Ca           2.6474700000      8.0080540000      1.9196440000    0    0    0
Ca           8.0417380000      2.5105950000      1.9196440000    0    0    0
Ca           8.0417380000      8.0080540000      1.9196440000    0    0    0
Ca           5.2793168386      0.1987298563      5.8973740206
Ca           5.2763106146      5.7014786051      5.9240175847
Ca          10.6734078500      0.1976240499      5.8976770323
Ca           0.0496100000      5.2593300000      1.9196450000    0    0    0
Ca           0.0496100000     10.7567880000      1.9196450000    0    0    0
Ca           5.4438780000      5.2593300000      1.9196450000    0    0    0
Ca           5.4438780000     10.7567880000      1.9196450000    0    0    0
Ti          -0.0174525392      2.6408981864      3.8092801210
Ti          -0.0171776099      8.1373124190      3.8122363795
Ti           5.3749012598      2.6423509552      3.8132806300
Ti           5.3775962684      8.1350368177      3.8227749125
Ti           0.0000000000      2.7487280000      0.0000000000    0    0    0
Ti           0.1078215564      2.8460270056      7.5622864404
Ti           0.0000000000      8.2461860000      0.0000000000    0    0    0
Ti           0.1159903467      8.3578089729      7.5616772394
Ti           5.3942680000      2.7487280000      0.0000000000    0    0    0
Ti           5.5094296785      2.8530670966      7.5651493874
Ti           5.3942680000      8.2461860000      0.0000000000    0    0    0
Ti           5.5164453397      8.3734465420      7.5961607244
Ti           2.7114005363     -0.1115693349      3.8099596249
Ti           2.7129001113      5.3868885252      3.8187665992
Ti           8.1044971555     -0.1076422143      3.8096205570
Ti           8.1070605757      5.3897855902      3.8112341519
Ti           2.6971090000      0.0000000000      0.0000000000    0    0    0
Ti           2.5733427328      0.1055987001      7.5626075559
Ti           2.6971090000      5.4974580000      0.0000000000    0    0    0
Ti           2.5585044768      5.5859283025      7.5846221547
Ti           8.0913760000      0.0000000000      0.0000000000    0    0    0
Ti           7.9687712478      0.1119764920      7.5631775705
Ti           8.0913760000      5.4974580000      0.0000000000    0    0    0
Ti           7.9725504744      5.6033239200      7.5615716781
O            0.4603761197      2.6525982408      5.7490894825
O            0.4699004802      8.1674613307      5.7471998836
O            5.8600771794      2.6571640572      5.7486241173
O            5.8496801467      8.1587690470      5.7615980568
O            3.1238100000      0.1116490000      1.9196440000    0    0    0
O            3.1238100000      5.6091080000      1.9196440000    0    0    0
O            8.5180770000      0.1116490000      1.9196440000    0    0    0
O            8.5180770000      5.6091080000      1.9196440000    0    0    0
O            1.1317856831      1.2075706692      3.4826583852
O            1.1313269169      6.7039882300      3.4884881246
O            6.5265661586      1.2089647185      3.4853576440
```

O	6.5276326474	6.7056089730	3.4883300319			
O	1.1266440000	1.1517870000	0.3184580000	0	0	0
O	1.1492521162	1.1494079196	7.9597492962			
O	1.1266440000	6.6492460000	0.3184580000	0	0	0
O	1.1412076594	6.6491686153	7.9486633504			
O	6.5209110000	1.1517870000	0.3184580000	0	0	0
O	6.5446599749	1.1534980845	7.9621924144			
O	6.5209110000	6.6492460000	0.3184580000	0	0	0
O	6.5474884573	6.6479568358	7.9589067427			
O	4.2666494057	4.3939294643	4.1767249514			
O	4.2624486527	9.8905343454	4.1755871452			
O	9.6590637690	4.3948352902	4.1786816859			
O	9.6544614327	9.8906406905	4.1770562280			
O	1.5631891187	3.9531035592	3.4850423910			
O	1.5610960691	9.4545386862	3.4791465261			
O	6.9602441246	3.9564210097	3.4827641393			
O	6.9541135913	9.4548261561	3.4875876360			
O	1.5704400000	3.9005160000	0.3184570000	0	0	0
O	1.5333999742	3.8965824103	7.9560168154			
O	1.5704400000	9.3979740000	0.3184570000	0	0	0
O	1.5499002044	9.3895860922	7.9725419809			
O	6.9647070000	3.9005160000	0.3184570000	0	0	0
O	6.9304791226	3.9044311502	7.9577467755			
O	6.9647070000	9.3979740000	0.3184570000	0	0	0
O	6.9479254610	9.4068122490	7.9586264616			
O	3.8252941662	1.6444526939	4.1791948078			
O	3.8234819181	7.1405769712	4.1793847735			
O	9.2192642338	1.6460102105	4.1763151661			
O	9.2177283252	7.1453532314	4.1784800423			
O	3.8911432477	1.4271760482	7.3849043433			
O	3.8797207484	6.9223289128	7.3862400555			
O	9.2872201285	1.4379648667	7.3919431368			
O	9.2879288627	6.9332909331	7.3881103329			
O	4.9675650000	2.8603790000	1.9196440000	0	0	0
O	4.9675650000	8.3578380000	1.9196440000	0	0	0
O	4.1917476213	4.1720440184	7.3957039904			
O	4.1981549852	9.6842767061	7.3858893962			
O	9.5850778007	4.1806890990	7.3919222199			
O	9.5919716475	9.6850459309	7.3919212772			
O	2.2303685846	5.3939418368	5.7537574633			
O	7.6205357574	5.4091911894	5.7477230975			
O	3.8237770000	1.5969400000	-0.3184580000	0	0	0
O	3.8237770000	7.0943990000	-0.3184580000	0	0	0
O	9.2180450000	1.5969400000	-0.3184580000	0	0	0
O	9.2180450000	7.0943990000	-0.3184580000	0	0	0
O	7.6223309973	-0.0850113232	5.7483715611			
O	-0.4267020000	2.8603790000	1.9196440000	0	0	0
O	-0.4267020000	8.3578380000	1.9196440000	0	0	0
O	4.2675730000	4.3456700000	-0.3184590000	0	0	0
O	4.2675730000	9.8431280000	-0.3184590000	0	0	0
O	9.6618410000	4.3456700000	-0.3184590000	0	0	0
O	9.6618410000	9.8431280000	-0.3184590000	0	0	0
O	2.2213589515	-0.0945440196	5.7486258864			
C	4.0554297068	6.9757197369	10.0449278518			
O	4.7041472413	7.9577657766	10.0202139267			
O	3.4079702419	5.9992373058	10.1355356626			

CO$_2$-L2

```
107
CO2_L2
Ca        -0.0986333816     5.6938296138     5.9226960483
Ca         2.7972243736     2.9580549378     5.9186894484
Ca         2.7986466238     8.4415154332     5.9039604257
Ca         8.1963297702     2.9531607342     5.8965535646
Ca         8.1985273629     8.4456790666     5.9026982252
Ca         2.6474700000     2.5105950000     1.9196440000   0   0   0
Ca         2.6474700000     8.0080540000     1.9196440000   0   0   0
Ca         8.0417380000     2.5105950000     1.9196440000   0   0   0
Ca         8.0417380000     8.0080540000     1.9196440000   0   0   0
Ca         5.2738698833     0.1972990577     5.9078561270
Ca         5.2655716131     5.6985091934     5.9025417404
Ca        10.6706277488     0.2046171670     5.9041307004
Ca         0.0496100000     5.2593300000     1.9196450000   0   0   0
Ca         0.0496100000    10.7567880000     1.9196450000   0   0   0
Ca         5.4438780000     5.2593300000     1.9196450000   0   0   0
Ca         5.4438780000    10.7567880000     1.9196450000   0   0   0
Ti        -0.0182157869     2.6426202628     3.8113993759
Ti        -0.0210745674     8.1378306397     3.8120801170
Ti         5.3719948982     2.6394539322     3.8119939589
Ti         5.3772711236     8.1379137301     3.8091639987
Ti         0.0000000000     2.7487280000     0.0000000000   0   0   0
Ti         0.1040316211     2.8564897754     7.5614105115
Ti         0.0000000000     8.2461860000     0.0000000000   0   0   0
Ti         0.1067096937     8.3602871980     7.5644082146
Ti         5.3942680000     2.7487280000     0.0000000000   0   0   0
Ti         5.5101471634     2.8581548293     7.5590456254
Ti         5.3942680000     8.2461860000     0.0000000000   0   0   0
Ti         5.5086424560     8.3568740455     7.5574200586
Ti         2.7102269614    -0.1070863831     3.8129383941
Ti         2.7093313224     5.3899044018     3.8243507070
Ti         8.1037942038    -0.1064269252     3.8103988131
Ti         8.1071861469     5.3900544111     3.8118694968
Ti         2.6971090000     0.0000000000     0.0000000000   0   0   0
Ti         2.5688147041     0.1086039637     7.5648095689
Ti         2.6971090000     5.4974580000     0.0000000000   0   0   0
Ti         2.5848948458     5.6184387069     7.6042779191
Ti         8.0913760000     0.0000000000     0.0000000000   0   0   0
Ti         7.9629314432     0.1111013919     7.5592758158
Ti         8.0913760000     5.4974580000     0.0000000000   0   0   0
Ti         7.9616163890     5.6076225128     7.5583558910
O          0.4566335979     2.6623429434     5.7469299951
O          0.4590915005     8.1615844274     5.7513612060
O          5.8598884531     2.6577077706     5.7446077451
O          5.8612221488     8.1656836419     5.7437876366
O          3.1238100000     0.1116490000     1.9196440000   0   0   0
O          3.1238100000     5.6091080000     1.9196440000   0   0   0
O          8.5180770000     0.1116490000     1.9196440000   0   0   0
O          8.5180770000     5.6091080000     1.9196440000   0   0   0
O          1.1290042151     1.2083737763     3.4863363175
O          1.1308454578     6.7071501237     3.4887244629
O          6.5249599765     1.2097386245     3.4808106148
O          6.5265368475     6.7085483259     3.4795027724
O          1.1266440000     1.1517870000     0.3184580000   0   0   0
O          1.1463504999     1.1619093128     7.9566360518
O          1.1266440000     6.6492460000     0.3184580000   0   0   0
```

O	1.1474257092	6.6615477650	7.9599295092			
O	6.5209110000	1.1517870000	0.3184580000	0	0	0
O	6.5416701398	1.1608835453	7.9570693705			
O	6.5209110000	6.6492460000	0.3184580000	0	0	0
O	6.5413543255	6.6620881641	7.9572750396			
O	4.2623087313	4.3926944926	4.1762110597			
O	4.2596064079	9.8925073168	4.1766635999			
O	9.6578202283	4.3996346752	4.1843533640			
O	9.6525515875	9.8919166871	4.1829665426			
O	1.5591639964	3.9580659172	3.4896448991			
O	1.5589709926	9.4560569415	3.4875794239			
O	6.9586051524	3.9572233858	3.4852401910			
O	6.9541334196	9.4569420932	3.4800373798			
O	1.5704400000	3.9005160000	0.3184570000	0	0	0
O	1.5184448410	3.9113859158	7.9505922697			
O	1.5704400000	9.3979740000	0.3184570000	0	0	0
O	1.5272551190	9.4078122420	7.9712296562			
O	6.9647070000	3.9005160000	0.3184570000	0	0	0
O	6.9288696512	3.9120344872	7.9556345105			
O	6.9647070000	9.3979740000	0.3184570000	0	0	0
O	6.9264449597	9.4115245715	7.9527578660			
O	3.8202276071	1.6468596950	4.1808204694			
O	3.8268350721	7.1426958062	4.1807248630			
O	9.2158893289	1.6482767164	4.1742453360			
O	9.2151764713	7.1468709578	4.1740318383			
O	3.8865666293	1.4342376650	7.3968035351			
O	3.8932946754	6.9412999924	7.3816467606			
O	9.2806509381	1.4368117455	7.3864604021			
O	9.2840782773	6.9339701276	7.3946810313			
O	4.9675650000	2.8603790000	1.9196440000	0	0	0
O	4.9675650000	8.3578380000	1.9196440000	0	0	0
O	4.1942613723	4.1793117324	7.3842500222			
O	4.1838294418	9.6796197616	7.3922058851			
O	9.5738579258	4.1855149494	7.3921189659			
O	9.5787474456	9.6851580306	7.3909333969			
O	2.2429530072	5.4127990449	5.7616706529			
O	7.6107185642	5.4173403021	5.7450285693			
O	3.8237770000	1.5969400000	-0.3184580000	0	0	0
O	3.8237770000	7.0943990000	-0.3184580000	0	0	0
O	9.2180450000	1.5969400000	-0.3184580000	0	0	0
O	9.2180450000	7.0943990000	-0.3184580000	0	0	0
O	7.6148214959	-0.0832747154	5.7449487123			
O	-0.4267020000	2.8603790000	1.9196440000	0	0	0
O	-0.4267020000	8.3578380000	1.9196440000	0	0	0
O	4.2675730000	4.3456700000	-0.3184590000	0	0	0
O	4.2675730000	9.8431280000	-0.3184590000	0	0	0
O	9.6618410000	4.3456700000	-0.3184590000	0	0	0
O	9.6618410000	9.8431280000	-0.3184590000	0	0	0
O	2.2224916790	-0.0890595280	5.7505289514			
C	2.7552066465	5.5882673926	11.1452705309			
O	2.4070933545	5.6209213719	12.2630512145			
O	3.1292663809	5.5544506365	10.0310984040			

CO$_2$-BC

107
CO2_BC

Ca	-0.1094637746	5.6987060411	5.8613080263			
Ca	2.7562563650	2.9713937345	5.8233430048			
Ca	2.8404098588	8.4088921409	5.8681114171			
Ca	8.1369524096	2.9493117052	5.8976640048			
Ca	8.1866618804	8.4373688746	5.8936568810			
Ca	2.6474700000	2.5105950000	1.9196440000	0	0	0
Ca	2.6474700000	8.0080540000	1.9196440000	0	0	0
Ca	8.0417380000	2.5105950000	1.9196440000	0	0	0
Ca	8.0417380000	8.0080540000	1.9196440000	0	0	0
Ca	5.2980113156	0.1647954538	5.8580617402			
Ca	5.3889636898	5.6109843656	5.9772585535			
Ca	10.6298279080	0.2416191643	5.8386436640			
Ca	0.0496100000	5.2593300000	1.9196450000	0	0	0
Ca	0.0496100000	10.7567880000	1.9196450000	0	0	0
Ca	5.4438780000	5.2593300000	1.9196450000	0	0	0
Ca	5.4438780000	10.7567880000	1.9196450000	0	0	0
Ti	-0.0343556453	2.6541754591	3.8015724147			
Ti	-0.0082226207	8.1481409878	3.7953138052			
Ti	5.3724352147	2.6372619290	3.8643872248			
Ti	5.3776828015	8.1223354595	3.8044713733			
Ti	0.0000000000	2.7487280000	0.0000000000	0	0	0
Ti	0.1057190255	2.8655616081	7.5568696552			
Ti	0.0000000000	8.2461860000	0.0000000000	0	0	0
Ti	0.1281162827	8.3672826314	7.5467950630			
Ti	5.3942680000	2.7487280000	0.0000000000	0	0	0
Ti	5.2035121591	2.6598067421	7.6748007719			
Ti	5.3942680000	8.2461860000	0.0000000000	0	0	0
Ti	5.5054814865	8.3649743729	7.5476490514			
Ti	2.7040654969	-0.1039297869	3.8001092331			
Ti	2.7286111297	5.3972662415	3.8079927462			
Ti	8.0986443815	-0.1138690731	3.7993089717			
Ti	8.1152034217	5.3960139378	3.8588123396			
Ti	2.6971090000	0.0000000000	0.0000000000	0	0	0
Ti	2.4681219555	0.0084612310	7.5712683860			
Ti	2.6971090000	5.4974580000	0.0000000000	0	0	0
Ti	2.5740723438	5.6079374849	7.5565044727			
Ti	8.0913760000	0.0000000000	0.0000000000	0	0	0
Ti	7.9686393272	0.1025293753	7.5543431034			
Ti	8.0913760000	5.4974580000	0.0000000000	0	0	0
Ti	8.1135640781	5.7062884065	7.6981100120			
O	0.4530420306	2.6507959555	5.7441824803			
O	0.4987239732	8.1688217547	5.7521058815			
O	5.7472792102	2.6078031672	5.7800797063			
O	5.8441604695	8.1935774275	5.7292677587			
O	3.1238100000	0.1116490000	1.9196440000	0	0	0
O	3.1238100000	5.6091080000	1.9196440000	0	0	0
O	8.5180770000	0.1116490000	1.9196440000	0	0	0
O	8.5180770000	5.6091080000	1.9196440000	0	0	0
O	1.1149162495	1.2003839565	3.4944495263			
O	1.1301231025	6.7035019010	3.4839369405			
O	6.5169422435	1.1943148369	3.5065063231			
O	6.5307964280	6.6922263081	3.5027475028			
O	1.1266440000	1.1517870000	0.3184580000	0	0	0
O	1.1283809299	1.1343118124	7.9571015739			
O	1.1266440000	6.6492460000	0.3184580000	0	0	0

O	1.1775929615	6.6626834351	7.9586825373			
O	6.5209110000	1.1517870000	0.3184580000	0	0	0
O	6.5345705092	1.1299486033	7.8809556221			
O	6.5209110000	6.6492460000	0.3184580000	0	0	0
O	6.6045117458	6.6890474629	7.8631620323			
O	4.2718806042	4.4009026203	4.1902876209			
O	4.2437365565	9.8842241365	4.1741986865			
O	9.6939987781	4.3984371108	4.1451138965			
O	9.6581126337	9.8886551777	4.1590153318			
O	1.5855697980	3.9434845819	3.5037217170			
O	1.5556272029	9.4516717697	3.4675019965			
O	6.9713622375	3.9391708504	3.5606627610			
O	6.9509838352	9.4482006429	3.4745454933			
O	1.5704400000	3.9005160000	0.3184570000	0	0	0
O	1.5461684369	3.8940341884	7.9281601866			
O	1.5704400000	9.3979740000	0.3184570000	0	0	0
O	1.5819454568	9.3804852392	8.0407377091			
O	6.9647070000	3.9005160000	0.3184570000	0	0	0
O	6.9259522920	3.8960687613	7.7389170210			
O	6.9647070000	9.3979740000	0.3184570000	0	0	0
O	6.9487461441	9.3981745339	7.9444962632			
O	3.7913156840	1.6313316102	4.1653019018			
O	3.8170342594	7.1429303359	4.1487758029			
O	9.2072304422	1.6506215087	4.1809581074			
O	9.2346156074	7.1547180466	4.1812910618			
O	3.8496429314	1.4233355478	7.4125114104			
O	3.9322222351	6.9059755622	7.3808141795			
O	9.2690082007	1.4505303714	7.3854487880			
O	9.3666075230	7.0039097860	7.4417279784			
O	4.9675650000	2.8603790000	1.9196440000	0	0	0
O	4.9675650000	8.3578380000	1.9196440000	0	0	0
O	4.1588094796	4.1545952813	7.3767868207			
O	4.1943834669	9.6860977012	7.3723804446			
O	9.6104099863	4.2028037593	7.3757468695			
O	9.6284344463	9.6936029875	7.3677488983			
O	2.2172264760	5.4256007205	5.7401095768			
O	7.7534636932	5.4480060677	5.7793936741			
O	3.8237770000	1.5969400000	-0.3184580000	0	0	0
O	3.8237770000	7.0943990000	-0.3184580000	0	0	0
O	9.2180450000	1.5969400000	-0.3184580000	0	0	0
O	9.2180450000	7.0943990000	-0.3184580000	0	0	0
O	7.6436166917	-0.1205890051	5.7352435142			
O	-0.4267020000	2.8603790000	1.9196440000	0	0	0
O	-0.4267020000	8.3578380000	1.9196440000	0	0	0
O	4.2675730000	4.3456700000	-0.3184590000	0	0	0
O	4.2675730000	9.8431280000	-0.3184590000	0	0	0
O	9.6618410000	4.3456700000	-0.3184590000	0	0	0
O	9.6618410000	9.8431280000	-0.3184590000	0	0	0
O	2.1809900558	-0.1346122590	5.7596893223			
C	6.8423530180	3.9927565381	9.0817551551			
O	7.6748839626	4.7955954890	9.6086629412			
O	5.8926120691	3.3069417105	9.5886398010			

CO_2-B1*

```
108
CO2_B1
Ca      -0.0921320894    5.7036950762    5.8989982188
Ca       2.8473645120    3.0227504635    5.9309982906
Ca       2.7906797503    8.4827957620    5.8274221856
Ca       8.2399248110    2.9677361868    5.8316440145
Ca       8.0908414926    8.4135546494    5.9132101864
Ca       2.6474700000    2.5105950000    1.9196440000   0   0   0
Ca       2.6474700000    8.0080540000    1.9196440000   0   0   0
Ca       8.0417380000    2.5105950000    1.9196440000   0   0   0
Ca       8.0417380000    8.0080540000    1.9196440000   0   0   0
Ca       5.2774840719    0.1258283863    5.8861620740
Ca       5.2765711659    5.8065077690    5.7870698400
Ca      10.6954897414    0.2086456103    5.8672138698
Ca       0.0496100000    5.2593300000    1.9196450000   0   0   0
Ca       0.0496100000   10.7567880000    1.9196450000   0   0   0
Ca       5.4438780000    5.2593300000    1.9196450000   0   0   0
Ca       5.4438780000   10.7567880000    1.9196450000   0   0   0
Ti       0.0112873336    2.6599914293    3.7984663471
Ti      -0.0453596944    8.1390757394    3.8107017872
Ti       5.3720071710    2.6352609031    3.8101955506
Ti       5.3769287793    8.1652493883    3.8467368201
Ti       0.0000000000    2.7487280000    0.0000000000   0   0   0
Ti       0.1548048321    2.8725496172    7.5448361341
Ti       0.0000000000    8.2461860000    0.0000000000   0   0   0
Ti       0.0899280249    8.3815813577    7.5576631098
Ti       5.3942680000    2.7487280000    0.0000000000   0   0   0
Ti       5.6723361079    2.6549818367    7.6181741417
Ti       5.3942680000    8.2461860000    0.0000000000   0   0   0
Ti       5.4057622089    8.3675787703    7.7227620865
Ti       2.7016683635   -0.0877425660    3.7943461839
Ti       2.6959902680    5.4028523202    3.8811031714
Ti       8.1169480387   -0.0992754809    3.8028725254
Ti       8.1004682450    5.3915951050    3.8027727925
Ti       2.6971090000    0.0000000000    0.0000000000   0   0   0
Ti       2.5863510184    0.1419109113    7.5596808073
Ti       2.6971090000    5.4974580000    0.0000000000   0   0   0
Ti       2.5288905652    5.7185767299    7.7469800167
Ti       8.0913760000    0.0000000000    0.0000000000   0   0   0
Ti       8.0448358728    0.0615288687    7.5374343971
Ti       8.0913760000    5.4974580000    0.0000000000   0   0   0
Ti       7.9452789383    5.5791261870    7.5420759705
O        0.5009023878    2.6419164977    5.7353244965
O        0.4346524781    8.1859789767    5.7552684971
O        5.8860640304    2.5844313019    5.7919840661
O        5.7690996564    8.1855092781    5.7477348147
O        3.1238100000    0.1116490000    1.9196440000   0   0   0
O        3.1238100000    5.6091080000    1.9196440000   0   0   0
O        8.5180770000    0.1116490000    1.9196440000   0   0   0
O        8.5180770000    5.6091080000    1.9196440000   0   0   0
O        1.1387007127    1.2032980190    3.4659346624
O        1.1054777340    6.6995234041    3.5221424882
O        6.5291124137    1.2051981904    3.4850427128
O        6.5184156486    6.6950291499    3.5189644593
O        1.1266440000    1.1517870000    0.3184580000   0   0   0
O        1.1504433344    1.1809774141    7.9679299734
O        1.1266440000    6.6492460000    0.3184580000   0   0   0
```

O	1.0502913537	6.6966851470	7.9636467369			
O	6.5209110000	1.1517870000	0.3184580000	0	0	0
O	6.5540500833	1.0734922883	8.0672331748			
O	6.5209110000	6.6492460000	0.3184580000	0	0	0
O	6.4445090496	6.5415927409	7.8539123811			
O	4.2625507536	4.3663855424	4.2119077472			
O	4.2529366995	9.9001341610	4.1595860091			
O	9.6582752872	4.3904969244	4.1818474136			
O	9.6601913140	9.8992071558	4.1697240332			
O	1.5475465201	3.9516556664	3.4712673393			
O	1.5628215883	9.4563809077	3.5090658403			
O	6.9565975715	3.9448981420	3.4906010762			
O	6.9614943063	9.4530004135	3.4942258846			
O	1.5704400000	3.9005160000	0.3184570000	0	0	0
O	1.5859879437	3.9303081177	7.8906166494			
O	1.5704400000	9.3979740000	0.3184570000	0	0	0
O	1.4789407401	9.4472854915	7.9391951416			
O	6.9647070000	3.9005160000	0.3184570000	0	0	0
O	6.9986130755	3.8237805720	7.9920999177			
O	6.9647070000	9.3979740000	0.3184570000	0	0	0
O	6.8815037836	9.3594100254	7.9274723427			
O	3.8235828006	1.6522512644	4.1856780692			
O	3.8070629577	7.1345370824	4.1360031245			
O	9.2351293234	1.6358642980	4.1688301767			
O	9.1954365564	7.1505786906	4.1832916730			
O	3.9048054343	1.4945138152	7.4032058662			
O	3.7922229844	6.9553413146	7.2018243019			
O	9.2723640845	1.4272749578	7.3480130340			
O	9.2160651417	6.9314068946	7.3873082240			
O	4.9675650000	2.8603790000	1.9196440000	0	0	0
O	4.9675650000	8.3578380000	1.9196440000	0	0	0
O	4.3658975853	4.2904519820	7.6499981001			
O	4.1360420398	9.7196460602	7.3963166785			
O	9.6155771009	4.1723818872	7.3628264135			
O	9.5187673534	9.7014306127	7.3976094732			
O	2.2521236295	5.3689991307	5.7620224441			
O	7.6135534272	5.3611632998	5.7314600917			
O	3.8237770000	1.5969400000	-0.3184580000	0	0	0
O	3.8237770000	7.0943990000	-0.3184580000	0	0	0
O	9.2180450000	1.5969400000	-0.3184580000	0	0	0
O	9.2180450000	7.0943990000	-0.3184580000	0	0	0
O	7.6130062262	-0.0928506546	5.7529132404			
O	-0.4267020000	2.8603790000	1.9196440000	0	0	0
O	-0.4267020000	8.3578380000	1.9196440000	0	0	0
O	4.2675730000	4.3456700000	-0.3184590000	0	0	0
O	4.2675730000	9.8431280000	-0.3184590000	0	0	0
O	9.6618410000	4.3456700000	-0.3184590000	0	0	0
O	9.6618410000	9.8431280000	-0.3184590000	0	0	0
O	2.2373076103	-0.0453133359	5.7515924541			
C	3.9766440355	6.9942064430	10.1465343869			
O	3.2913140832	6.0458077761	9.7140285237			
O	4.7035212169	7.8787054013	9.6672593328			
H	4.9488593204	4.9290053264	8.1258090286			

CO_2-B2*

109

CO2_B2

Ca	-0.0818349523	5.7557664122	5.7801862922			
Ca	2.8598679012	3.0214152913	5.9038758308			
Ca	2.7963805720	8.5202910743	5.7377730263			
Ca	8.2577804299	2.9438374094	5.7311987490			
Ca	8.1507753092	8.4654613052	5.8256434047			
Ca	2.6474700000	2.5105950000	1.9196440000	0	0	0
Ca	2.6474700000	8.0080540000	1.9196440000	0	0	0
Ca	8.0417380000	2.5105950000	1.9196440000	0	0	0
Ca	8.0417380000	8.0080540000	1.9196440000	0	0	0
Ca	5.2751501812	0.1985306594	5.8075385088			
Ca	5.2549432863	5.7688904917	5.8049528911			
Ca	10.6801065362	0.2353418205	5.7951450459			
Ca	0.0496100000	5.2593300000	1.9196450000	0	0	0
Ca	0.0496100000	10.7567880000	1.9196450000	0	0	0
Ca	5.4438780000	5.2593300000	1.9196450000	0	0	0
Ca	5.4438780000	10.7567880000	1.9196450000	0	0	0
Ti	0.0274174659	2.7365493185	3.7911493742			
Ti	-0.0207994566	8.2262172285	3.8041255461			
Ti	5.3850273847	2.7186212286	3.8002924819			
Ti	5.3910793317	8.2229260463	3.8287979153			
Ti	0.0000000000	2.7487280000	0.0000000000	0	0	0
Ti	0.1884370898	2.8277133280	7.5576056689			
Ti	0.0000000000	8.2461860000	0.0000000000	0	0	0
Ti	0.1160745493	8.4223182108	7.5571857371			
Ti	5.3942680000	2.7487280000	0.0000000000	0	0	0
Ti	5.7038848774	2.6576685378	7.6249390880			
Ti	5.3942680000	8.2461860000	0.0000000000	0	0	0
Ti	5.4098188911	8.4118124679	7.6260591676			
Ti	2.6935348886	-0.0087037968	3.7895967274			
Ti	2.6834680188	5.4727356026	3.8545808433			
Ti	8.0958490406	-0.0311014698	3.7936565863			
Ti	8.0845976561	5.4686723960	3.7950714333			
Ti	2.6971090000	0.0000000000	0.0000000000	0	0	0
Ti	2.5998577030	0.1422409723	7.5539535249			
Ti	2.6971090000	5.4974580000	0.0000000000	0	0	0
Ti	2.4889769161	5.7628839816	7.6627694325			
Ti	8.0913760000	0.0000000000	0.0000000000	0	0	0
Ti	8.0521498675	0.0608853350	7.5337672169			
Ti	8.0913760000	5.4974580000	0.0000000000	0	0	0
Ti	8.1321749980	5.4663797597	7.5145284968			
O	0.5069956793	2.6552590340	5.7530798641			
O	0.4508301396	8.2102315008	5.7594447274			
O	5.9118099818	2.6191828807	5.8123817887			
O	5.8382036030	8.1175786662	5.7842853947			
O	3.1238100000	0.1116490000	1.9196440000	0	0	0
O	3.1238100000	5.6091080000	1.9196440000	0	0	0
O	8.5180770000	0.1116490000	1.9196440000	0	0	0
O	8.5180770000	5.6091080000	1.9196440000	0	0	0
O	1.1538604614	1.1844878798	3.4845877553			
O	1.1241558809	6.6747819069	3.5427810450			
O	6.5371549306	1.1854084031	3.5102613205			
O	6.5402975903	6.6713959961	3.5246353563			
O	1.1266440000	1.1517870000	0.3184580000	0	0	0
O	1.1506517192	1.2081313739	7.9829554150			
O	1.1266440000	6.6492460000	0.3184580000	0	0	0

O	1.0069620176	6.7127764804	7.9411154102			
O	6.5209110000	1.1517870000	0.3184580000	0	0	0
O	6.5682904105	1.0893334118	8.0753165368			
O	6.5209110000	6.6492460000	0.3184580000	0	0	0
O	6.4554012410	6.5382225065	8.1075334941			
O	4.2586552446	4.3148889681	4.2398115160			
O	4.2477922743	9.8306643561	4.1940757628			
O	9.6473578950	4.3272741026	4.1970557024			
O	9.6357487105	9.8289342842	4.1860737340			
O	1.5520164112	3.9324003377	3.4696842242			
O	1.5597657287	9.4360059173	3.5161356246			
O	6.9541194017	3.9223333584	3.5006286730			
O	6.9530379770	9.4270388629	3.5072461546			
O	1.5704400000	3.9005160000	0.3184570000	0	0	0
O	1.5271665751	3.9586872515	7.9476437932			
O	1.5704400000	9.3979740000	0.3184570000	0	0	0
O	1.5026753357	9.4650797669	7.9738392071			
O	6.9647070000	3.9005160000	0.3184570000	0	0	0
O	6.9409524139	3.8789504376	8.0991550901			
O	6.9647070000	9.3979740000	0.3184570000	0	0	0
O	6.9010425353	9.3649007775	7.9315286628			
O	3.8537692735	1.5926071942	4.2106473890			
O	3.8372860389	7.0758717996	4.1474410559			
O	9.2633545548	1.5628835395	4.1704869584			
O	9.2316588598	7.0769196551	4.2002980053			
O	3.9111951983	1.4741283243	7.4097248727			
O	3.7908451377	6.9938947563	7.1616654923			
O	9.2694662417	1.4533869771	7.3358944788			
O	9.1716312820	6.9566941859	7.3605944290			
O	4.9675650000	2.8603790000	1.9196440000	0	0	0
O	4.9675650000	8.3578380000	1.9196440000	0	0	0
O	4.2690220273	4.2290220093	7.5730445310			
O	4.1520757985	9.7427829078	7.3772994226			
O	9.5644850833	4.2160727182	7.3567693548			
O	9.5409054826	9.7275755490	7.3867613475			
O	2.2314246956	5.3667607157	5.7681029793			
O	7.5986083963	5.3443719984	5.7732061908			
O	3.8237770000	1.5969400000	-0.3184580000	0	0	0
O	3.8237770000	7.0943990000	-0.3184580000	0	0	0
O	9.2180450000	1.5969400000	-0.3184580000	0	0	0
O	9.2180450000	7.0943990000	-0.3184580000	0	0	0
O	7.6128437551	-0.0878917147	5.7579304100			
O	-0.4267020000	2.8603790000	1.9196440000	0	0	0
O	-0.4267020000	8.3578380000	1.9196440000	0	0	0
O	4.2675730000	4.3456700000	-0.3184590000	0	0	0
O	4.2675730000	9.8431280000	-0.3184590000	0	0	0
O	9.6618410000	4.3456700000	-0.3184590000	0	0	0
O	9.6618410000	9.8431280000	-0.3184590000	0	0	0
O	2.2295149321	-0.0438473513	5.7654778167			
C	4.0504393355	7.1564275155	9.8053321213			
O	4.3118394832	7.9004844206	10.7177290704			
O	3.4146218710	6.0814576642	9.5920234927			
H	4.2626001517	4.4566870074	8.5266554960			
H	5.8996896551	5.9317090057	8.6307710863			

CO_2-B3*

```
109
CO2_B3
Ca     0.0219269317     5.6704302221    5.8707003850
Ca     2.8250620396     3.0539631897    5.8950468940
Ca     2.7684939257     8.5114476466    5.7630099667
Ca     8.2343644412     2.9093739020    5.8184161592
Ca     8.1187901229     8.4505773083    5.9052640202
Ca     2.6474700000     2.5105950000    1.9196440000   0   0   0
Ca     2.6474700000     8.0080540000    1.9196440000   0   0   0
Ca     8.0417380000     2.5105950000    1.9196440000   0   0   0
Ca     8.0417380000     8.0080540000    1.9196440000   0   0   0
Ca     5.2779392443     0.2330118157    5.8597314330
Ca     5.3034307883     5.7406422146    5.7994603015
Ca    10.6518063650     0.2411112761    5.8743985841
Ca     0.0496100000     5.2593300000    1.9196450000   0   0   0
Ca     0.0496100000    10.7567880000    1.9196450000   0   0   0
Ca     5.4438780000     5.2593300000    1.9196450000   0   0   0
Ca     5.4438780000    10.7567880000    1.9196450000   0   0   0
Ti     0.0383129225     2.6750004260    3.7899289092
Ti    -0.0506361428     8.1368439377    3.8107985612
Ti     5.3623260063     2.6711725802    3.8007786798
Ti     5.3978651834     8.1518308993    3.8667504838
Ti     0.0000000000     2.7487280000    0.0000000000   0   0   0
Ti     0.1907295902     2.8194422635    7.5969920047
Ti     0.0000000000     8.2461860000    0.0000000000   0   0   0
Ti     0.0819413154     8.4122921837    7.5574412655
Ti     5.3942680000     2.7487280000    0.0000000000   0   0   0
Ti     5.5714776037     2.8192133296    7.5920054768
Ti     5.3942680000     8.2461860000    0.0000000000   0   0   0
Ti     5.3670328951     8.4373090636    7.7171538838
Ti     2.7098696880    -0.0822875901    3.7933592734
Ti     2.7036322856     5.4299402950    3.9013634412
Ti     8.1143262303    -0.0878039255    3.8045163060
Ti     8.1214569142     5.3949279668    3.7941450110
Ti     2.6971090000     0.0000000000    0.0000000000   0   0   0
Ti     2.5956591969     0.1454142422    7.5398150147
Ti     2.6971090000     5.4974580000    0.0000000000   0   0   0
Ti     2.9483765083     5.6309873734    7.8985187986
Ti     8.0913760000     0.0000000000    0.0000000000   0   0   0
Ti     7.9526325612     0.0973421492    7.5671120065
Ti     8.0913760000     5.4974580000    0.0000000000   0   0   0
Ti     8.1248110874     5.4736435105    7.5221999043
O      0.4706528530     2.6688481849    5.7703520875
O      0.4488630313     8.0998385417    5.7740642193
O      5.8768460391     2.6946150724    5.7795546105
O      5.8249360714     8.1189697851    5.7670556017
O      3.1238100000     0.1116490000    1.9196440000   0   0   0
O      3.1238100000     5.6091080000    1.9196440000   0   0   0
O      8.5180770000     0.1116490000    1.9196440000   0   0   0
O      8.5180770000     5.6091080000    1.9196440000   0   0   0
O      1.1449055139     1.2055669854    3.4726932571
O      1.0948088175     6.6952013508    3.5145833402
O      6.5329146849     1.2163926548    3.5078892451
O      6.5253937675     6.6830608702    3.5217604137
O      1.1266440000     1.1517870000    0.3184580000   0   0   0
O      1.1310956403     1.1856289934    7.9973019144
O      1.1266440000     6.6492460000    0.3184580000   0   0   0
```

O	1.0413359011	6.6193408233	8.1186540379			
O	6.5209110000	1.1517870000	0.3184580000	0	0	0
O	6.5040422568	1.1599080178	7.9657339394			
O	6.5209110000	6.6492460000	0.3184580000	0	0	0
O	6.4607423608	6.5260838398	8.0542439441			
O	4.2916908336	4.3777474937	4.1610214516			
O	4.2524252595	9.9042198585	4.1887148353			
O	9.6681485456	4.4003401497	4.1964468571			
O	9.6579030015	9.8882399555	4.1908889727			
O	1.5418383345	3.9317024716	3.4711144515			
O	1.5687330890	9.4499468437	3.5298745177			
O	6.9799806412	3.9497955525	3.4962174785			
O	6.9599114995	9.4546812680	3.5015896893			
O	1.5704400000	3.9005160000	0.3184570000	0	0	0
O	1.4911141205	3.9733593024	7.9759159755			
O	1.5704400000	9.3979740000	0.3184570000	0	0	0
O	1.5093763634	9.4237658063	7.9075322897			
O	6.9647070000	3.9005160000	0.3184570000	0	0	0
O	6.9231857127	3.9089837137	8.0496075393			
O	6.9647070000	9.3979740000	0.3184570000	0	0	0
O	6.9013271061	9.4032400913	7.9594053818			
O	3.8354545498	1.6534413638	4.1936793475			
O	3.8080407429	7.1489806244	4.1131201098			
O	9.2466340505	1.6285899504	4.1738857263			
O	9.1875353009	7.1573443953	4.1757133441			
O	3.8652570410	1.5019606506	7.3834898284			
O	3.8002108282	6.9924806271	7.0680001799			
O	9.2496931020	1.4377716456	7.3899070328			
O	9.2255061850	6.9415129842	7.4417579712			
O	4.9675650000	2.8603790000	1.9196440000	0	0	0
O	4.9675650000	8.3578380000	1.9196440000	0	0	0
O	4.2198280043	4.2672225927	7.4489821759			
O	4.1345057333	9.7715341352	7.3579793027			
O	9.5567731694	4.2137937411	7.4171724634			
O	9.5564601435	9.6980283212	7.3718458058			
O	2.2627071401	5.3581422816	5.6974864565			
O	7.6468093857	5.3768059839	5.7660349529			
O	3.8237770000	1.5969400000	-0.3184580000	0	0	0
O	3.8237770000	7.0943990000	-0.3184580000	0	0	0
O	9.2180450000	1.5969400000	-0.3184580000	0	0	0
O	9.2180450000	7.0943990000	-0.3184580000	0	0	0
O	7.6111635159	-0.0736490831	5.7559446502			
O	-0.4267020000	2.8603790000	1.9196440000	0	0	0
O	-0.4267020000	8.3578380000	1.9196440000	0	0	0
O	4.2675730000	4.3456700000	-0.3184590000	0	0	0
O	4.2675730000	9.8431280000	-0.3184590000	0	0	0
O	9.6618410000	4.3456700000	-0.3184590000	0	0	0
O	9.6618410000	9.8431280000	-0.3184590000	0	0	0
O	2.2084383629	-0.0216465470	5.7474375277			
C	4.2924826304	7.5897331844	9.5566691206			
O	4.2124984021	8.2410810163	10.5762991042			
O	3.6787141460	6.3130624935	9.4798113093			
H	5.8799952379	5.9808366384	8.6235711913			
H	0.4785272283	5.9673991816	8.5817130901			

HCOO$^-$

109
HCOO

Ca	-0.0621698289	5.6898619140	5.9056832578			
Ca	2.8738451415	3.0251774406	5.9239298609			
Ca	2.8041121973	8.4795817292	5.8104694733			
Ca	8.2647641892	2.9505670043	5.8378590239			
Ca	8.0915347715	8.3876247630	5.9320068253			
Ca	2.6474700000	2.5105950000	1.9196440000	0	0	0
Ca	2.6474700000	8.0080540000	1.9196440000	0	0	0
Ca	8.0417380000	2.5105950000	1.9196440000	0	0	0
Ca	8.0417380000	8.0080540000	1.9196440000	0	0	0
Ca	5.2968996354	0.1038295074	5.8923528592			
Ca	5.3021439252	5.8014142854	5.7645932138			
Ca	10.7102381549	0.1977357147	5.8618670254			
Ca	0.0496100000	5.2593300000	1.9196450000	0	0	0
Ca	0.0496100000	10.7567880000	1.9196450000	0	0	0
Ca	5.4438780000	5.2593300000	1.9196450000	0	0	0
Ca	5.4438780000	10.7567880000	1.9196450000	0	0	0
Ti	0.0194124289	2.6595614861	3.7979127313			
Ti	-0.0432557441	8.1336549369	3.8122300721			
Ti	5.3782045282	2.6311879342	3.8099451075			
Ti	5.3840167432	8.1651900628	3.8500607225			
Ti	0.0000000000	2.7487280000	0.0000000000	0	0	0
Ti	0.1934614115	2.8646689816	7.5452317054			
Ti	0.0000000000	8.2461860000	0.0000000000	0	0	0
Ti	0.1088631643	8.3702447103	7.5558971540			
Ti	5.3942680000	2.7487280000	0.0000000000	0	0	0
Ti	5.6940029357	2.6404132913	7.6157640402			
Ti	5.3942680000	8.2461860000	0.0000000000	0	0	0
Ti	5.3981475121	8.3440530604	7.7410178239			
Ti	2.7062429480	-0.0888842592	3.7930812097			
Ti	2.7004695180	5.4015491814	3.8872665129			
Ti	8.1247153363	-0.1070114336	3.8020835048			
Ti	8.1106180254	5.3829225024	3.8028405932			
Ti	2.6971090000	0.0000000000	0.0000000000	0	0	0
Ti	2.6051701524	0.1346195868	7.5607227186			
Ti	2.6971090000	5.4974580000	0.0000000000	0	0	0
Ti	2.5756648234	5.7245806996	7.7686618525			
Ti	8.0913760000	0.0000000000	0.0000000000	0	0	0
Ti	8.0633741317	0.0428861108	7.5368235740			
Ti	8.0913760000	5.4974580000	0.0000000000	0	0	0
Ti	7.9657582778	5.5551230493	7.5421054614			
O	0.5214157529	2.6303573654	5.7349666817			
O	0.4498927750	8.1790104276	5.7540394481			
O	5.9081682939	2.5760267898	5.7930773008			
O	5.7736845475	8.1893947992	5.7453478189			
O	3.1238100000	0.1116490000	1.9196440000	0	0	0
O	3.1238100000	5.6091080000	1.9196440000	0	0	0
O	8.5180770000	0.1116490000	1.9196440000	0	0	0
O	8.5180770000	5.6091080000	1.9196440000	0	0	0
O	1.1479631544	1.1985113640	3.4667544569			
O	1.1094936508	6.6951754656	3.5205616542			
O	6.5384364777	1.2021312870	3.4868331864			
O	6.5248315244	6.6872224000	3.5290214425			
O	1.1266440000	1.1517870000	0.3184580000	0	0	0
O	1.1736249100	1.1779039453	7.9883192281			
O	1.1266440000	6.6492460000	0.3184580000	0	0	0

O	1.0873418447	6.6930158472	7.9625417006			
O	6.5209110000	1.1517870000	0.3184580000	0	0	0
O	6.5701228633	1.0531834972	8.0649164388			
O	6.5209110000	6.6492460000	0.3184580000	0	0	0
O	6.4647768299	6.5209757038	7.8453321232			
O	4.2774412699	4.3605734205	4.2041469683			
O	4.2624126134	9.8995402648	4.1580635085			
O	9.6725500196	4.3830808308	4.1823977761			
O	9.6748911021	9.8958453902	4.1606901533			
O	1.5539439022	3.9456418970	3.4702461256			
O	1.5730107108	9.4516096394	3.5144019192			
O	6.9675482792	3.9397216743	3.4882772047			
O	6.9667361065	9.4510590215	3.4858292056			
O	1.5704400000	3.9005160000	0.3184570000	0	0	0
O	1.6226950311	3.9295582244	7.8762465747			
O	1.5704400000	9.3979740000	0.3184570000	0	0	0
O	1.4856240813	9.4458348465	7.9467955551			
O	6.9647070000	3.9005160000	0.3184570000	0	0	0
O	7.0284191377	3.8017297145	8.0024708725			
O	6.9647070000	9.3979740000	0.3184570000	0	0	0
O	6.8804035639	9.3328324560	7.9285046247			
O	3.8342559480	1.6470039500	4.1889304240			
O	3.8136919884	7.1327204368	4.1271637372			
O	9.2493877324	1.6257060960	4.1749856838			
O	9.2020522749	7.1447712287	4.1877111173			
O	3.9235813226	1.4894980418	7.4019880065			
O	3.8165667846	6.9459785219	7.1567881427			
O	9.2891900305	1.4046002257	7.3464665951			
O	9.2417666963	6.9056839503	7.3926532629			
O	4.9675650000	2.8603790000	1.9196440000	0	0	0
O	4.9675650000	8.3578380000	1.9196440000	0	0	0
O	4.4022870122	4.2893208801	7.6496999127			
O	4.1402325509	9.7089147253	7.4071238758			
O	9.6454426535	4.1562303568	7.3703077152			
O	9.5267956185	9.6779790272	7.4024633493			
O	2.2702948903	5.3612524071	5.7514137897			
O	7.6335178335	5.3392625170	5.7326750978			
O	3.8237770000	1.5969400000	-0.3184580000	0	0	0
O	3.8237770000	7.0943990000	-0.3184580000	0	0	0
O	9.2180450000	1.5969400000	-0.3184580000	0	0	0
O	9.2180450000	7.0943990000	-0.3184580000	0	0	0
O	7.6278484153	-0.1170179328	5.7534674846			
O	-0.4267020000	2.8603790000	1.9196440000	0	0	0
O	-0.4267020000	8.3578380000	1.9196440000	0	0	0
O	4.2675730000	4.3456700000	-0.3184590000	0	0	0
O	4.2675730000	9.8431280000	-0.3184590000	0	0	0
O	9.6618410000	4.3456700000	-0.3184590000	0	0	0
O	9.6618410000	9.8431280000	-0.3184590000	0	0	0
O	2.2507466732	-0.0533152272	5.7564276852			
C	4.0490432383	6.9392454629	10.2032405845			
O	3.3125034551	6.0446762649	9.6761164660			
O	4.7371641889	7.8409193785	9.6388162579			
H	5.0049547098	4.9292436986	8.1006829659			
H	4.0977261662	6.9180088071	11.3103478870			

HCOOH

109
HCOOH

Ca	-0.1182845713	5.6904293934	5.9067707609			
Ca	2.8137376818	2.9524288142	5.8986003239			
Ca	2.8073110514	8.4708439380	5.8523856088			
Ca	8.2196656708	2.9358671440	5.9090540848			
Ca	8.1604265450	8.4204735867	5.9433500368			
Ca	2.6474700000	2.5105950000	1.9196440000	0	0	0
Ca	2.6474700000	8.0080540000	1.9196440000	0	0	0
Ca	8.0417380000	2.5105950000	1.9196440000	0	0	0
Ca	8.0417380000	8.0080540000	1.9196440000	0	0	0
Ca	5.2887317442	0.1716871802	5.9156980362			
Ca	5.3092073378	5.7096337684	5.8775298514			
Ca	10.6672449816	0.1974256194	5.9099654243			
Ca	0.0496100000	5.2593300000	1.9196450000	0	0	0
Ca	0.0496100000	10.7567880000	1.9196450000	0	0	0
Ca	5.4438780000	5.2593300000	1.9196450000	0	0	0
Ca	5.4438780000	10.7567880000	1.9196450000	0	0	0
Ti	-0.0110271787	2.6470263387	3.8116550642			
Ti	-0.0272220990	8.1358322242	3.8151459505			
Ti	5.3788808505	2.6353992607	3.8102449339			
Ti	5.3790195654	8.1413772748	3.8417888573			
Ti	0.0000000000	2.7487280000	0.0000000000	0	0	0
Ti	0.1416857747	2.8590699436	7.5776110987			
Ti	0.0000000000	8.2461860000	0.0000000000	0	0	0
Ti	0.1166766993	8.3510050532	7.5607461443			
Ti	5.3942680000	2.7487280000	0.0000000000	0	0	0
Ti	5.5150428735	2.8484365508	7.5497499137			
Ti	5.3942680000	8.2461860000	0.0000000000	0	0	0
Ti	5.4680665536	8.3565133142	7.6417832053			
Ti	2.7077451460	-0.1006989456	3.8053088879			
Ti	2.7092192624	5.3855903709	3.8215197408			
Ti	8.1088386668	-0.1148393868	3.8128825559			
Ti	8.1090014802	5.3848680601	3.8069439062			
Ti	2.6971090000	0.0000000000	0.0000000000	0	0	0
Ti	2.5748691505	0.1084251496	7.5631783210			
Ti	2.6971090000	5.4974580000	0.0000000000	0	0	0
Ti	2.5980226423	5.6246107875	7.5475552652			
Ti	8.0913760000	0.0000000000	0.0000000000	0	0	0
Ti	7.9740047975	0.1049351997	7.5675823684			
Ti	8.0913760000	5.4974580000	0.0000000000	0	0	0
Ti	7.9774882116	5.5822221276	7.5612962259			
O	0.4720644124	2.6684924892	5.7576886289			
O	0.4638866234	8.1635753307	5.7488170713			
O	5.8796317540	2.6490734074	5.7435456122			
O	5.8269904064	8.1900442022	5.7596087891			
O	3.1238100000	0.1116490000	1.9196440000	0	0	0
O	3.1238100000	5.6091080000	1.9196440000	0	0	0
O	8.5180770000	0.1116490000	1.9196440000	0	0	0
O	8.5180770000	5.6091080000	1.9196440000	0	0	0
O	1.1371964071	1.2083013382	3.4850983639			
O	1.1252452758	6.7072130115	3.4882212078			
O	6.5299772277	1.2064097258	3.4793453021			
O	6.5276419242	6.6992197461	3.5093231936			
O	1.1266440000	1.1517870000	0.3184580000	0	0	0
O	1.1551672305	1.1628040480	7.9825548256			
O	1.1266440000	6.6492460000	0.3184580000	0	0	0

O	1.1622082270	6.6587317737	7.9494136638			
O	6.5209110000	1.1517870000	0.3184580000	0	0	0
O	6.5441991701	1.1473085666	7.9625956690			
O	6.5209110000	6.6492460000	0.3184580000	0	0	0
O	6.5438882422	6.6335086518	7.8965414017			
O	4.2638391841	4.3877255844	4.1742468121			
O	4.2561884072	9.8944452327	4.1796258314			
O	9.6682148806	4.3930326753	4.1664923760			
O	9.6643508780	9.8953656935	4.1699727797			
O	1.5595070075	3.9510455980	3.4639992797			
O	1.5674284693	9.4538210082	3.4927718620			
O	6.9588441935	3.9564169893	3.4789546076			
O	6.9581210015	9.4544047607	3.4844590136			
O	1.5704400000	3.9005160000	0.3184570000	0	0	0
O	1.5480231825	3.9091047636	8.0025041911			
O	1.5704400000	9.3979740000	0.3184570000	0	0	0
O	1.5205124555	9.4132269589	7.9787215515			
O	6.9647070000	3.9005160000	0.3184570000	0	0	0
O	6.9304200943	3.8978970771	7.9844135565			
O	6.9647070000	9.3979740000	0.3184570000	0	0	0
O	6.9057517093	9.3958497706	7.9691257359			
O	3.8285005172	1.6442978362	4.1788821543			
O	3.8193359710	7.1376108356	4.1567070008			
O	9.2264441935	1.6449142008	4.1823693636			
O	9.2124216505	7.1464132803	4.1846305564			
O	3.8962528795	1.4353756634	7.3926604881			
O	3.8655173480	6.9336308214	7.2268003713			
O	9.2873245061	1.4235616406	7.4048764090			
O	9.2903798956	6.9192823285	7.4123188086			
O	4.9675650000	2.8603790000	1.9196440000	0	0	0
O	4.9675650000	8.3578380000	1.9196440000	0	0	0
O	4.2032083018	4.1796661831	7.3690630531			
O	4.1660688063	9.6867575359	7.4091423174			
O	9.5924212262	4.1712873054	7.4101606238			
O	9.5706511938	9.6678480538	7.4000006325			
O	2.1953616697	5.3584872774	5.7314747943			
O	7.6539852518	5.3789654374	5.7461614525			
O	3.8237770000	1.5969400000	-0.3184580000	0	0	0
O	3.8237770000	7.0943990000	-0.3184580000	0	0	0
O	9.2180450000	1.5969400000	-0.3184580000	0	0	0
O	9.2180450000	7.0943990000	-0.3184580000	0	0	0
O	7.6271114928	-0.0943658592	5.7534898117			
O	-0.4267020000	2.8603790000	1.9196440000	0	0	0
O	-0.4267020000	8.3578380000	1.9196440000	0	0	0
O	4.2675730000	4.3456700000	-0.3184590000	0	0	0
O	4.2675730000	9.8431280000	-0.3184590000	0	0	0
O	9.6618410000	4.3456700000	-0.3184590000	0	0	0
O	9.6618410000	9.8431280000	-0.3184590000	0	0	0
O	2.2195060196	-0.0776501010	5.7534215506			
C	4.1776774037	6.9342726343	10.4118126660			
O	3.5108810048	5.9120062008	9.8160037434			
O	4.7527287519	7.8115026478	9.8070297840			
H	4.1531713475	6.8896280964	11.5174055152			
H	3.1264182158	5.3040171351	10.4810912995			

CO-v1

106
CO_v1

Ca	-0.0841349126	5.6861255348	5.9227238718			
Ca	2.7990992363	2.9621991761	5.9233734777			
Ca	2.8054063458	8.4463014202	5.9046347839			
Ca	8.1999446065	2.9566067500	5.8930700831			
Ca	8.2062284165	8.4471365125	5.8967184802			
Ca	2.6474700000	2.5105950000	1.9196440000	0	0	0
Ca	2.6474700000	8.0080540000	1.9196440000	0	0	0
Ca	8.0417380000	2.5105950000	1.9196440000	0	0	0
Ca	8.0417380000	8.0080540000	1.9196440000	0	0	0
Ca	5.2748225295	0.2056744744	5.9008821385			
Ca	5.2716444318	5.6995754249	5.9014568329			
Ca	10.6765690978	0.2067500171	5.8957429745			
Ca	0.0496100000	5.2593300000	1.9196450000	0	0	0
Ca	0.0496100000	10.7567880000	1.9196450000	0	0	0
Ca	5.4438780000	5.2593300000	1.9196450000	0	0	0
Ca	5.4438780000	10.7567880000	1.9196450000	0	0	0
Ti	-0.0146858562	2.6443714751	3.8082756668			
Ti	-0.0178115275	8.1362233941	3.8103510059			
Ti	5.3719357777	2.6436658269	3.8111977846			
Ti	5.3793302027	8.1392987732	3.8088169091			
Ti	0.0000000000	2.7487280000	0.0000000000	0	0	0
Ti	0.1008154900	2.8491398006	7.5598312345			
Ti	0.0000000000	8.2461860000	0.0000000000	0	0	0
Ti	0.1082936762	8.3673050934	7.5599134835			
Ti	5.3942680000	2.7487280000	0.0000000000	0	0	0
Ti	5.5188911956	2.8578578531	7.5631577647			
Ti	5.3942680000	8.2461860000	0.0000000000	0	0	0
Ti	5.5231733784	8.3636874334	7.5606262216			
Ti	2.7124790942	-0.1063746551	3.8115897291			
Ti	2.7102631056	5.3875135981	3.8342561791			
Ti	8.1055530145	-0.1043671997	3.8084008599			
Ti	8.1107003355	5.3908354103	3.8118820227			
Ti	2.6971090000	0.0000000000	0.0000000000	0	0	0
Ti	2.5749093667	0.1095731985	7.5654647167			
Ti	2.6971090000	5.4974580000	0.0000000000	0	0	0
Ti	2.5992302961	5.6228636968	7.6200605911			
Ti	8.0913760000	0.0000000000	0.0000000000	0	0	0
Ti	7.9655616849	0.1124209242	7.5599156983			
Ti	8.0913760000	5.4974580000	0.0000000000	0	0	0
Ti	7.9700348992	5.6095807064	7.5591536599			
O	0.4557429803	2.6586879311	5.7465147837			
O	0.4640527307	8.1619757902	5.7495446541			
O	5.8624346244	2.6622148974	5.7480334928			
O	5.8670521716	8.1671510407	5.7473999467			
O	3.1238100000	0.1116490000	1.9196440000	0	0	0
O	3.1238100000	5.6091080000	1.9196440000	0	0	0
O	8.5180770000	0.1116490000	1.9196440000	0	0	0
O	8.5180770000	5.6091080000	1.9196440000	0	0	0
O	1.1303841165	1.2066257098	3.4867162158			
O	1.1320760131	6.7059909236	3.4899001341			
O	6.5256141141	1.2099414970	3.4833929348			
O	6.5280312778	6.7081736584	3.4833579072			
O	1.1266440000	1.1517870000	0.3184580000	0	0	0
O	1.1514231308	1.1600266172	7.9572152793			
O	1.1266440000	6.6492460000	0.3184580000	0	0	0

O	1.1511992770	6.6638587151	7.9519053867			
O	6.5209110000	1.1517870000	0.3184580000	0	0	0
O	6.5462727837	1.1621259535	7.9619152326			
O	6.5209110000	6.6492460000	0.3184580000	0	0	0
O	6.5484051588	6.6640208418	7.9592627102			
O	4.2735054733	4.3936317314	4.1690889844			
O	4.2627361049	9.8931582780	4.1761200076			
O	9.6615270264	4.3996568555	4.1850033105			
O	9.6542377572	9.8926226361	4.1817501125			
O	1.5613144922	3.9532909139	3.4858501284			
O	1.5617382376	9.4558707895	3.4872666719			
O	6.9646155650	3.9560830753	3.4883637415			
O	6.9557392166	9.4575604646	3.4805972617			
O	1.5704400000	3.9005160000	0.3184570000	0	0	0
O	1.5180017923	3.9084393986	7.9412696098			
O	1.5704400000	9.3979740000	0.3184570000	0	0	0
O	1.5361294207	9.4063824704	7.9666933906			
O	6.9647070000	3.9005160000	0.3184570000	0	0	0
O	6.9354989244	3.9135812910	7.9585452458			
O	6.9647070000	9.3979740000	0.3184570000	0	0	0
O	6.9427854042	9.4121277586	7.9605511630			
O	3.8232076868	1.6475313608	4.1827380350			
O	3.8306540667	7.1451166575	4.1820343365			
O	9.2183955583	1.6480239741	4.1726620821			
O	9.2176111504	7.1469932292	4.1727904952			
O	3.8916930411	1.4357939479	7.3972941917			
O	3.8985239081	6.9458711380	7.3963335887			
O	9.2852615004	1.4394316860	7.3879458749			
O	9.2925129632	6.9363889453	7.3942467748			
O	4.9675650000	2.8603790000	1.9196440000	0	0	0
O	4.9675650000	8.3578380000	1.9196440000	0	0	0
O	4.2010584570	4.1833403339	7.3966064435			
O	4.1968259017	9.6829603221	7.3926422445			
O	9.5772008173	4.1856382610	7.3918951050			
O	9.5876928365	9.6899453567	7.3855635206			
O	2.2559714263	5.4013330462	5.7496716143			
O	7.6161146356	5.4192393539	5.7469566753			
O	3.8237770000	1.5969400000	-0.3184580000	0	0	0
O	3.8237770000	7.0943990000	-0.3184580000	0	0	0
O	9.2180450000	1.5969400000	-0.3184580000	0	0	0
O	9.2180450000	7.0943990000	-0.3184580000	0	0	0
O	7.6163196616	-0.0810528263	5.7477542054			
O	-0.4267020000	2.8603790000	1.9196440000	0	0	0
O	-0.4267020000	8.3578380000	1.9196440000	0	0	0
O	4.2675730000	4.3456700000	-0.3184590000	0	0	0
O	4.2675730000	9.8431280000	-0.3184590000	0	0	0
O	9.6618410000	4.3456700000	-0.3184590000	0	0	0
O	9.6618410000	9.8431280000	-0.3184590000	0	0	0
O	2.2294013719	-0.0891377275	5.7505427412			
C	2.7220026835	5.5652294739	9.9881976585			
O	2.5518716034	5.5806230098	11.1141644009			

CO-v2

```
106
CO_v2
Ca           -0.0992361076        5.6892261700        5.9159691791
Ca            2.8028372581        2.9522549746        5.9136072380
Ca            2.8022990265        8.4401991991        5.9083919920
Ca            8.1970356984        2.9483517873        5.9019223220
Ca            8.1979258266        8.4428084371        5.9060657210
Ca            2.6474700000        2.5105950000        1.9196440000    0    0    0
Ca            2.6474700000        8.0080540000        1.9196440000    0    0    0
Ca            8.0417380000        2.5105950000        1.9196440000    0    0    0
Ca            8.0417380000        8.0080540000        1.9196440000    0    0    0
Ca            5.2767704168        0.1990635130        5.9089517395
Ca            5.2777007363        5.6927227049        5.9141879740
Ca           10.6743549474        0.1988699038        5.9049980756
Ca            0.0496100000        5.2593300000        1.9196450000    0    0    0
Ca            0.0496100000       10.7567880000        1.9196450000    0    0    0
Ca            5.4438780000        5.2593300000        1.9196450000    0    0    0
Ca            5.4438780000       10.7567880000        1.9196450000    0    0    0
Ti           -0.0168747362        2.6407674795        3.8102459513
Ti           -0.0192482599        8.1368868813        3.8113101500
Ti            5.3740272658        2.6407362747        3.8131001227
Ti            5.3773757978        8.1371189154        3.8119765802
Ti            0.0000000000        2.7487280000        0.0000000000    0    0    0
Ti            0.1095029322        2.8538144943        7.5621003864
Ti            0.0000000000        8.2461860000        0.0000000000    0    0    0
Ti            0.1108859311        8.3575390188        7.5622650558
Ti            5.3942680000        2.7487280000        0.0000000000    0    0    0
Ti            5.5109188941        2.8550916636        7.5675994093
Ti            5.3942680000        8.2461860000        0.0000000000    0    0    0
Ti            5.5110424138        8.3556961002        7.5662719046
Ti            2.7128351311       -0.1093249328        3.8118186761
Ti            2.7130927825        5.3902662458        3.8209654268
Ti            8.1059286480       -0.1079407208        3.8111318038
Ti            8.1073653921        5.3898808497        3.8126841996
Ti            2.6971090000        0.0000000000        0.0000000000    0    0    0
Ti            2.5732204949        0.1064612400        7.5650822281
Ti            2.6971090000        5.4974580000        0.0000000000    0    0    0
Ti            2.5830027388        5.6124472881        7.5890037334
Ti            8.0913760000        0.0000000000        0.0000000000    0    0    0
Ti            7.9670933174        0.1088622760        7.5652856699
Ti            8.0913760000        5.4974580000        0.0000000000    0    0    0
Ti            7.9696095037        5.6064505049        7.5645739694
O             0.4591590466        2.6630825566        5.7469509926
O             0.4610599326        8.1638540404        5.7478382131
O             5.8572895553        2.6601295052        5.7508508807
O             5.8576476126        8.1597517841        5.7504442274
O             3.1238100000        0.1116490000        1.9196440000    0    0    0
O             3.1238100000        5.6091080000        1.9196440000    0    0    0
O             8.5180770000        0.1116490000        1.9196440000    0    0    0
O             8.5180770000        5.6091080000        1.9196440000    0    0    0
O             1.1330030364        1.2092933023        3.4849286095
O             1.1323271681        6.7058929714        3.4901557058
O             6.5262618956        1.2090322675        3.4849748590
O             6.5273920750        6.7077637913        3.4832302635
O             1.1266440000        1.1517870000        0.3184580000    0    0    0
O             1.1515929491        1.1593618222        7.9557878710
O             1.1266440000        6.6492460000        0.3184580000    0    0    0
```

O	1.1494039760	6.6600513640	7.9460663316			
O	6.5209110000	1.1517870000	0.3184580000	0	0	0
O	6.5452734545	1.1585201729	7.9633401573			
O	6.5209110000	6.6492460000	0.3184580000	0	0	0
O	6.5467357136	6.6577486542	7.9620451570			
O	4.2657034973	4.3928284350	4.1770688911			
O	4.2624901165	9.8914275065	4.1781206962			
O	9.6552908222	4.3941663471	4.1798910040			
O	9.6563509197	9.8921719710	4.1774445450			
O	1.5607412080	3.9588654582	3.4878535123			
O	1.5615389404	9.4555123716	3.4827037589			
O	6.9564493672	3.9581977534	3.4875354112			
O	6.9551909655	9.4552527179	3.4858188116			
O	1.5704400000	3.9005160000	0.3184570000	0	0	0
O	1.5285051563	3.9076028989	7.9473313823			
O	1.5704400000	9.3979740000	0.3184570000	0	0	0
O	1.5344572506	9.4043347103	7.9628851770			
O	6.9647070000	3.9005160000	0.3184570000	0	0	0
O	6.9296001865	3.9104676862	7.9587123263			
O	6.9647070000	9.3979740000	0.3184570000	0	0	0
O	6.9313494193	9.4070194522	7.9608113298			
O	3.8239514596	1.6448787952	4.1800391546			
O	3.8254751714	7.1435934323	4.1757014398			
O	9.2194543238	1.6448439340	4.1767980221			
O	9.2175718948	7.1444686370	4.1754780573			
O	3.8921109865	1.4318369675	7.3961327062			
O	3.8952915058	6.9368015945	7.3955483886			
O	9.2856986111	1.4356003197	7.3931945481			
O	9.2890629301	6.9328548023	7.3952277767			
O	4.9675650000	2.8603790000	1.9196440000	0	0	0
O	4.9675650000	8.3578380000	1.9196440000	0	0	0
O	4.1919624426	4.1791013814	7.3979158650			
O	4.1894378769	9.6792142461	7.3961891295			
O	9.5805092731	4.1839992090	7.3932617270			
O	9.5826602885	9.6824497731	7.3907988586			
O	2.2459735005	5.4125855647	5.7557665277			
O	7.6199899679	5.4163905765	5.7497292408			
O	3.8237770000	1.5969400000	-0.3184580000	0	0	0
O	3.8237770000	7.0943990000	-0.3184580000	0	0	0
O	9.2180450000	1.5969400000	-0.3184580000	0	0	0
O	9.2180450000	7.0943990000	-0.3184580000	0	0	0
O	7.6190256469	-0.0841345189	5.7504935562			
O	-0.4267020000	2.8603790000	1.9196440000	0	0	0
O	-0.4267020000	8.3578380000	1.9196440000	0	0	0
O	4.2675730000	4.3456700000	-0.3184590000	0	0	0
O	4.2675730000	9.8431280000	-0.3184590000	0	0	0
O	9.6618410000	4.3456700000	-0.3184590000	0	0	0
O	9.6618410000	9.8431280000	-0.3184590000	0	0	0
O	2.2268670738	-0.0906218891	5.7496064632			
O	2.9202715663	5.5663673113	10.1735255709			
C	2.6184532892	5.5864200873	11.2744972592			

CO-v3

```
106
CO_v3
Ca        -0.0903808130     5.6551091212     5.9248290394
Ca         2.7444314652     2.9185413901     5.9533956220
Ca         2.8156141822     8.4855922688     5.8628371160
Ca         8.1604514841     2.9596741790     5.8513876081
Ca         8.2442913624     8.4265065329     5.8921678635
Ca         2.6474700000     2.5105950000     1.9196440000   0   0   0
Ca         2.6474700000     8.0080540000     1.9196440000   0   0   0
Ca         8.0417380000     2.5105950000     1.9196440000   0   0   0
Ca         8.0417380000     8.0080540000     1.9196440000   0   0   0
Ca         5.2680317960     0.2340301165     5.8505523342
Ca         5.3177242113     5.7026620379     5.8569819349
Ca        10.6960161489     0.1942013545     5.8755145803
Ca         0.0496100000     5.2593300000     1.9196450000   0   0   0
Ca         0.0496100000    10.7567880000     1.9196450000   0   0   0
Ca         5.4438780000     5.2593300000     1.9196450000   0   0   0
Ca         5.4438780000    10.7567880000     1.9196450000   0   0   0
Ti        -0.0068684578     2.6515067241     3.8774259537
Ti        -0.0072091281     8.1302578704     3.8078463458
Ti         5.3623245769     2.6514004947     3.8083565296
Ti         5.3843949307     8.1523506086     3.8030657287
Ti         0.0000000000     2.7487280000     0.0000000000   0   0   0
Ti        -0.1497240027     2.7074040413     7.6682962923
Ti         0.0000000000     8.2461860000     0.0000000000   0   0   0
Ti         0.1381808678     8.3558439762     7.5601903440
Ti         5.3942680000     2.7487280000     0.0000000000   0   0   0
Ti         5.5002239423     2.8732687283     7.5617338602
Ti         5.3942680000     8.2461860000     0.0000000000   0   0   0
Ti         5.5255514494     8.3579715311     7.5396273126
Ti         2.7025209578    -0.0959774856     3.7995138249
Ti         2.7214458308     5.3830180669     3.8102849335
Ti         8.1113245885    -0.1056493926     3.8081182043
Ti         8.1160066088     5.3957290288     3.8019857722
Ti         2.6971090000     0.0000000000     0.0000000000   0   0   0
Ti         2.5901180852     0.1020411225     7.5589349491
Ti         2.6971090000     5.4974580000     0.0000000000   0   0   0
Ti         2.6563330093     5.6735426008     7.5564728655
Ti         8.0913760000     0.0000000000     0.0000000000   0   0   0
Ti         7.9017768549     0.0338998931     7.5721021978
Ti         8.0913760000     5.4974580000     0.0000000000   0   0   0
Ti         7.9756089404     5.6119095676     7.5690936757
O          0.4364519647     2.6988127610     5.7601733303
O          0.4753105944     8.1544748634     5.7439697018
O          5.8504376100     2.6726947060     5.7470887868
O          5.9047545247     8.1749595798     5.7410663890
O          3.1238100000     0.1116490000     1.9196440000   0   0   0
O          3.1238100000     5.6091080000     1.9196440000   0   0   0
O          8.5180770000     0.1116490000     1.9196440000   0   0   0
O          8.5180770000     5.6091080000     1.9196440000   0   0   0
O          1.1351635317     1.1975348168     3.5037760974
O          1.1488497220     6.7012194983     3.4908501873
O          6.5150376973     1.2053917104     3.5058492000
O          6.5316553536     6.7041134503     3.4886923746
O          1.1266440000     1.1517870000     0.3184580000   0   0   0
O          1.1674690535     1.1507290973     7.8630759108
O          1.1266440000     6.6492460000     0.3184580000   0   0   0
```

O	1.1978590404	6.6619974378	7.9419553131			
O	6.5209110000	1.1517870000	0.3184580000	0	0	0
O	6.5481172700	1.1481512271	7.9382121523			
O	6.5209110000	6.6492460000	0.3184580000	0	0	0
O	6.5577775166	6.6631283917	7.9494682096			
O	4.2875869683	4.4060748733	4.1542712304			
O	4.2660148853	9.8889478380	4.1652141603			
O	9.6655475774	4.3977644354	4.1664857841			
O	9.6539716178	9.8798080158	4.1783541947			
O	1.5783452200	3.9532777530	3.5003797386			
O	1.5650320926	9.4499187431	3.4853941612			
O	6.9704291168	3.9497881811	3.5014755427			
O	6.9545702873	9.4584001982	3.4665577119			
O	1.5704400000	3.9005160000	0.3184570000	0	0	0
O	1.6587191057	3.9418461732	8.1218431558			
O	1.5704400000	9.3979740000	0.3184570000	0	0	0
O	1.5785167409	9.3987934976	7.9421980220			
O	6.9647070000	3.9005160000	0.3184570000	0	0	0
O	6.9370135945	3.9048241934	7.9460509833			
O	6.9647070000	9.3979740000	0.3184570000	0	0	0
O	6.9784538421	9.3885152017	8.0013386085			
O	3.8141157770	1.6587439309	4.1887034994			
O	3.8421371151	7.1417824914	4.1930217125			
O	9.2132774034	1.6285435623	4.1899156126			
O	9.2318919876	7.1356105227	4.1700518708			
O	3.9064531349	1.4442660481	7.3937856082			
O	3.9439938365	6.9828700138	7.3522221167			
O	9.2645959046	1.4269374331	7.4119072002			
O	9.3162667767	6.9212805012	7.3973695841			
O	4.9675650000	2.8603790000	1.9196440000	0	0	0
O	4.9675650000	8.3578380000	1.9196440000	0	0	0
O	4.2097128673	4.2133958360	7.3758443191			
O	4.2404766466	9.6917855614	7.3647969330			
O	9.5651941920	4.1870342978	7.3847488048			
O	9.6191470126	9.6824111652	7.3775398737			
O	2.2454703625	5.3742875232	5.7739471496			
O	7.6414294332	5.4189381453	5.7476327321			
O	3.8237770000	1.5969400000	-0.3184580000	0	0	0
O	3.8237770000	7.0943990000	-0.3184580000	0	0	0
O	9.2180450000	1.5969400000	-0.3184580000	0	0	0
O	9.2180450000	7.0943990000	-0.3184580000	0	0	0
O	7.6083282705	-0.1271893432	5.7541770681			
O	-0.4267020000	2.8603790000	1.9196440000	0	0	0
O	-0.4267020000	8.3578380000	1.9196440000	0	0	0
O	4.2675730000	4.3456700000	-0.3184590000	0	0	0
O	4.2675730000	9.8431280000	-0.3184590000	0	0	0
O	9.6618410000	4.3456700000	-0.3184590000	0	0	0
O	9.6618410000	9.8431280000	-0.3184590000	0	0	0
O	2.2572030552	-0.1012947619	5.7360544008			
C	0.9487835172	3.5737540433	9.2488815265			
O	1.2311173444	3.8910808492	10.3832057134			

CO-h1

106						
CO_h1						
Ca	-0.0994327013	5.7113681557	5.8453213206			
Ca	2.8142957201	2.9319698026	5.8825997372			
Ca	2.8118420134	8.5242209766	5.8329541523			
Ca	8.1635844178	2.9504680900	5.8669530738			
Ca	8.1866196536	8.4375172829	5.9047318961			
Ca	2.6474700000	2.5105950000	1.9196440000	0	0	0
Ca	2.6474700000	8.0080540000	1.9196440000	0	0	0
Ca	8.0417380000	2.5105950000	1.9196440000	0	0	0
Ca	8.0417380000	8.0080540000	1.9196440000	0	0	0
Ca	5.2745806596	0.2339492874	5.8601766502			
Ca	5.2822929791	5.7166673366	5.8799145739			
Ca	10.6881099112	0.1794852356	5.8728934332			
Ca	0.0496100000	5.2593300000	1.9196450000	0	0	0
Ca	0.0496100000	10.7567880000	1.9196450000	0	0	0
Ca	5.4438780000	5.2593300000	1.9196450000	0	0	0
Ca	5.4438780000	10.7567880000	1.9196450000	0	0	0
Ti	-0.0227224418	2.6495031213	3.7991963755			
Ti	-0.0223663450	8.1410943260	3.8051336792			
Ti	5.3735512332	2.6582045176	3.8007525869			
Ti	5.3865000495	8.1485921339	3.8625060491			
Ti	0.0000000000	2.7487280000	0.0000000000	0	0	0
Ti	0.0135772570	2.7778719919	7.5347890436			
Ti	0.0000000000	8.2461860000	0.0000000000	0	0	0
Ti	0.1285886223	8.3546302243	7.5412790204			
Ti	5.3942680000	2.7487280000	0.0000000000	0	0	0
Ti	5.4955767325	2.8861059366	7.5648592229			
Ti	5.3942680000	8.2461860000	0.0000000000	0	0	0
Ti	5.5518917713	8.4692343760	7.6816409243			
Ti	2.7082128412	-0.0891632697	3.7955484225			
Ti	2.6996366384	5.3878641906	3.8720042261			
Ti	8.1018157901	-0.1092978537	3.8054213186			
Ti	8.1011145748	5.3922171045	3.8000197127			
Ti	2.6971090000	0.0000000000	0.0000000000	0	0	0
Ti	2.5836946559	0.1183211169	7.5694557551			
Ti	2.6971090000	5.4974580000	0.0000000000	0	0	0
Ti	2.3909274284	5.3856483664	7.7613925914			
Ti	8.0913760000	0.0000000000	0.0000000000	0	0	0
Ti	7.9506196139	0.1072026157	7.5741308636			
Ti	8.0913760000	5.4974580000	0.0000000000	0	0	0
Ti	7.9730953012	5.6102519443	7.5278733779			
O	0.4702679802	2.6416644392	5.7586564001			
O	0.4851045904	8.1590989423	5.7310181790			
O	5.8388809512	2.6756282345	5.7452547190			
O	5.8484121979	8.2245700461	5.7500287936			
O	3.1238100000	0.1116490000	1.9196440000	0	0	0
O	3.1238100000	5.6091080000	1.9196440000	0	0	0
O	8.5180770000	0.1116490000	1.9196440000	0	0	0
O	8.5180770000	5.6091080000	1.9196440000	0	0	0
O	1.1301758821	1.2060783603	3.4806085447			
O	1.1256012983	6.6916871467	3.5122073690			
O	6.5286248095	1.2075851505	3.5032195658			
O	6.5289529864	6.7009769441	3.5020510142			
O	1.1266440000	1.1517870000	0.3184580000	0	0	0
O	1.1439861582	1.1211381600	7.9597578213			
O	1.1266440000	6.6492460000	0.3184580000	0	0	0

O	1.0766190160	6.5883462364	7.9237608338			
O	6.5209110000	1.1517870000	0.3184580000	0	0	0
O	6.5626915419	1.1831726932	7.9423617356			
O	6.5209110000	6.6492460000	0.3184580000	0	0	0
O	6.5643021684	6.6855970511	7.8178846409			
O	4.2744885622	4.3919842314	4.1694479726			
O	4.2528104433	9.8973825008	4.1650780168			
O	9.6635957654	4.3844332376	4.1572491712			
O	9.6535389275	9.8852646551	4.1747471380			
O	1.5615961799	3.9461486932	3.4889317271			
O	1.5636981100	9.4540092993	3.4941349062			
O	6.9597964828	3.9463965527	3.4746507033			
O	6.9594546594	9.4592816236	3.4796307726			
O	1.5704400000	3.9005160000	0.3184570000	0	0	0
O	1.5050917121	3.8193631115	8.0948717183			
O	1.5704400000	9.3979740000	0.3184570000	0	0	0
O	1.5879011790	9.3595994416	7.9220452717			
O	6.9647070000	3.9005160000	0.3184570000	0	0	0
O	6.9389172379	3.9236323518	7.9431650205			
O	6.9647070000	9.3979740000	0.3184570000	0	0	0
O	7.0148716158	9.4592965534	8.0851808859			
O	3.8241937304	1.6465363974	4.1663891649			
O	3.8245830432	7.1323997009	4.1996756604			
O	9.2237820654	1.6406477287	4.1833920344			
O	9.2188579206	7.1415467033	4.1764851149			
O	3.8940802194	1.4546459492	7.3964870571			
O	3.8501859122	6.9867351039	7.2848353175			
O	9.3144728849	1.4556067368	7.3941942296			
O	9.2889847310	6.9264390400	7.3498127680			
O	4.9675650000	2.8603790000	1.9196440000	0	0	0
O	4.9675650000	8.3578380000	1.9196440000	0	0	0
O	4.1904491308	4.1981018718	7.3923054436			
O	4.2313846990	9.7421854771	7.3842158659			
O	9.6034838449	4.1746026571	7.3462321496			
O	9.6220324550	9.6822661265	7.3672034283			
O	2.2327339094	5.3092859187	5.8021509342			
O	7.6286602710	5.3722060941	5.7154370528			
O	3.8237770000	1.5969400000	-0.3184580000	0	0	0
O	3.8237770000	7.0943990000	-0.3184580000	0	0	0
O	9.2180450000	1.5969400000	-0.3184580000	0	0	0
O	9.2180450000	7.0943990000	-0.3184580000	0	0	0
O	7.6197684949	-0.0960327823	5.7636399863			
O	-0.4267020000	2.8603790000	1.9196440000	0	0	0
O	-0.4267020000	8.3578380000	1.9196440000	0	0	0
O	4.2675730000	4.3456700000	-0.3184590000	0	0	0
O	4.2675730000	9.8431280000	-0.3184590000	0	0	0
O	9.6618410000	4.3456700000	-0.3184590000	0	0	0
O	9.6618410000	9.8431280000	-0.3184590000	0	0	0
O	2.2407006027	-0.0657825773	5.7437777231			
C	4.1152867361	7.1365695547	8.6203014007			
O	3.5040818815	6.3203740243	9.3535966532			

CO-h2

106
CO_h2

Ca	-0.1139954039	5.7065675469	5.8810082248			
Ca	2.7724816517	2.9660663413	5.8359011717			
Ca	2.8243571579	8.4067720970	5.8811381913			
Ca	8.1695904528	2.9552120345	5.8411348526			
Ca	8.1900163378	8.4182433079	5.9121997699			
Ca	2.6474700000	2.5105950000	1.9196440000	0	0	0
Ca	2.6474700000	8.0080540000	1.9196440000	0	0	0
Ca	8.0417380000	2.5105950000	1.9196440000	0	0	0
Ca	8.0417380000	8.0080540000	1.9196440000	0	0	0
Ca	5.2861737424	0.1578084484	5.8638325124			
Ca	5.3879558380	5.6258889454	5.9623034356			
Ca	10.6424640239	0.2348622986	5.8413503861			
Ca	0.0496100000	5.2593300000	1.9196450000	0	0	0
Ca	0.0496100000	10.7567880000	1.9196450000	0	0	0
Ca	5.4438780000	5.2593300000	1.9196450000	0	0	0
Ca	5.4438780000	10.7567880000	1.9196450000	0	0	0
Ti	-0.0220766493	2.6530809942	3.7976836980			
Ti	-0.0067428620	8.1485429367	3.7944436508			
Ti	5.3753861231	2.6333719664	3.8567676760			
Ti	5.3758415004	8.1239789557	3.8041298092			
Ti	0.0000000000	2.7487280000	0.0000000000	0	0	0
Ti	0.1161144126	2.8658652104	7.5539615727			
Ti	0.0000000000	8.2461860000	0.0000000000	0	0	0
Ti	0.1053349152	8.3606425304	7.5547044829			
Ti	5.3942680000	2.7487280000	0.0000000000	0	0	0
Ti	5.2370979657	2.6569670612	7.6630726242			
Ti	5.3942680000	8.2461860000	0.0000000000	0	0	0
Ti	5.5029869964	8.3667622742	7.5498301409			
Ti	2.7093131556	-0.1003375069	3.8013252754			
Ti	2.7280064171	5.3959078513	3.8128201718			
Ti	8.0986506323	-0.1160937833	3.8023557153			
Ti	8.1174780111	5.4104088715	3.8631232986			
Ti	2.6971090000	0.0000000000	0.0000000000	0	0	0
Ti	2.4698485605	0.0086587991	7.5834889326			
Ti	2.6971090000	5.4974580000	0.0000000000	0	0	0
Ti	2.5863999990	5.6044195442	7.5568558982			
Ti	8.0913760000	0.0000000000	0.0000000000	0	0	0
Ti	7.9727992681	0.1166558500	7.5514612932			
Ti	8.0913760000	5.4974580000	0.0000000000	0	0	0
Ti	8.0836512215	5.6860781438	7.6999704103			
O	0.4617990401	2.6507172375	5.7422806421			
O	0.4810920273	8.1628248963	5.7590109778			
O	5.7358245203	2.5568932821	5.7779237895			
O	5.8508372005	8.1812483697	5.7348302505			
O	3.1238100000	0.1116490000	1.9196440000	0	0	0
O	3.1238100000	5.6091080000	1.9196440000	0	0	0
O	8.5180770000	0.1116490000	1.9196440000	0	0	0
O	8.5180770000	5.6091080000	1.9196440000	0	0	0
O	1.1186699078	1.1996542537	3.4905571104			
O	1.1333514689	6.7044617118	3.4893661236			
O	6.5202075002	1.1975216093	3.4908885363			
O	6.5229794766	6.6936070985	3.4921024176			
O	1.1266440000	1.1517870000	0.3184580000	0	0	0
O	1.1312202988	1.1369283813	7.9698744934			
O	1.1266440000	6.6492460000	0.3184580000	0	0	0

O	1.2051083230	6.6680036382	7.9615232388			
O	6.5209110000	1.1517870000	0.3184580000	0	0	0
O	6.5542660827	1.1595767659	7.9401955452			
O	6.5209110000	6.6492460000	0.3184580000	0	0	0
O	6.6081509541	6.7004210098	7.9121761902			
O	4.2702416794	4.3951458456	4.1970033455			
O	4.2439710468	9.8814743102	4.1753756858			
O	9.6869613495	4.3959877884	4.1600996274			
O	9.6536503315	9.8899492996	4.1681645889			
O	1.5819352176	3.9448997940	3.4972093868			
O	1.5523952277	9.4559402533	3.4760932165			
O	6.9679232740	3.9381552608	3.5810787367			
O	6.9479295601	9.4496372028	3.4775618407			
O	1.5704400000	3.9005160000	0.3184570000	0	0	0
O	1.5672207432	3.8877025703	7.9219890508			
O	1.5704400000	9.3979740000	0.3184570000	0	0	0
O	1.5727279878	9.3849698185	8.0317930172			
O	6.9647070000	3.9005160000	0.3184570000	0	0	0
O	6.9533942358	3.9199675094	7.5792489732			
O	6.9647070000	9.3979740000	0.3184570000	0	0	0
O	6.9423035938	9.4129565500	7.9334790751			
O	3.7859318320	1.6300545817	4.1553938244			
O	3.8168612727	7.1424338779	4.1535805222			
O	9.2179247697	1.6453382590	4.1676316284			
O	9.2317431168	7.1646141037	4.1609273028			
O	3.8671395844	1.4246279792	7.4452582933			
O	3.9450605705	6.9050382250	7.3720395893			
O	9.2775062527	1.4526652070	7.3648180557			
O	9.3837160361	6.9998990950	7.4875269240			
O	4.9675650000	2.8603790000	1.9196440000	0	0	0
O	4.9675650000	8.3578380000	1.9196440000	0	0	0
O	4.1886437692	4.1558397557	7.3837494539			
O	4.1889732363	9.6825808084	7.3780714707			
O	9.6238852298	4.2037711423	7.3978461196			
O	9.6202075359	9.7025441821	7.3710384867			
O	2.2208501603	5.4210243219	5.7400437598			
O	7.7666430883	5.5236322289	5.7590443822			
O	3.8237770000	1.5969400000	-0.3184580000	0	0	0
O	3.8237770000	7.0943990000	-0.3184580000	0	0	0
O	9.2180450000	1.5969400000	-0.3184580000	0	0	0
O	9.2180450000	7.0943990000	-0.3184580000	0	0	0
O	7.6288898653	-0.0972674721	5.7370851243			
O	-0.4267020000	2.8603790000	1.9196440000	0	0	0
O	-0.4267020000	8.3578380000	1.9196440000	0	0	0
O	4.2675730000	4.3456700000	-0.3184590000	0	0	0
O	4.2675730000	9.8431280000	-0.3184590000	0	0	0
O	9.6618410000	4.3456700000	-0.3184590000	0	0	0
O	9.6618410000	9.8431280000	-0.3184590000	0	0	0
O	2.1965474439	-0.1198530643	5.7667331247			
C	6.9660918468	4.1777968817	8.9064624667			
O	6.0848061230	3.5279337719	9.5351916576			

CO-v4*

```
110
CO_v4
Ca          -0.0319615496     5.6916135640     5.8011837428
Ca           2.7995778956     3.0580112745     5.6464304531
Ca           2.7763786974     8.5046038563     5.6199894348
Ca           8.1790843403     2.9829480898     5.6576101240
Ca           8.2311004483     8.5054999695     5.7525972923
Ca           2.6474700000     2.5105950000     1.9196440000    0   0   0
Ca           2.6474700000     8.0080540000     1.9196440000    0   0   0
Ca           8.0417380000     2.5105950000     1.9196440000    0   0   0
Ca           8.0417380000     8.0080540000     1.9196440000    0   0   0
Ca           5.3031492250     0.2145429354     5.6689431604
Ca           5.2414426416     5.7779026545     5.7927742397
Ca          10.6794869589     0.1853128041     5.7303215760
Ca           0.0496100000     5.2593300000     1.9196450000    0   0   0
Ca           0.0496100000    10.7567880000     1.9196450000    0   0   0
Ca           5.4438780000     5.2593300000     1.9196450000    0   0   0
Ca           5.4438780000    10.7567880000     1.9196450000    0   0   0
Ti          -0.0003538484     2.7312886032     3.7723217422
Ti          -0.0140951578     8.2121362910     3.7842335301
Ti           5.3931219463     2.7512504766     3.7742648913
Ti           5.4070582919     8.2323209784     3.7714673142
Ti           0.0000000000     2.7487280000     0.0000000000    0   0   0
Ti          -0.0618799604     2.6778947703     7.5353038816
Ti           0.0000000000     8.2461860000     0.0000000000    0   0   0
Ti           0.0730902865     8.2904634341     7.5532728243
Ti           5.3942680000     2.7487280000     0.0000000000    0   0   0
Ti           5.4698301217     2.8327836128     7.6215272291
Ti           5.3942680000     8.2461860000     0.0000000000    0   0   0
Ti           5.5615903810     8.4662408846     7.5761135623
Ti           2.6992960343    -0.0057070554     3.7730644795
Ti           2.6966843015     5.4972709323     3.8589456137
Ti           8.0964663560    -0.0185804880     3.7768663107
Ti           8.1072348884     5.4917797921     3.7880487301
Ti           2.6971090000     0.0000000000     0.0000000000    0   0   0
Ti           2.4821714658    -0.1168166758     7.6364420411
Ti           2.6971090000     5.4974580000     0.0000000000    0   0   0
Ti           2.5509585646     5.5492560253     7.8524682451
Ti           8.0913760000     0.0000000000     0.0000000000    0   0   0
Ti           7.9289978785     0.0554478337     7.5924313265
Ti           8.0913760000     5.4974580000     0.0000000000    0   0   0
Ti           7.9657845953     5.6818834559     7.5566004693
O            0.4839855986     2.6208024100     5.8223035083
O            0.4689924826     8.1196715353     5.7907627510
O            5.8073636414     2.6478537955     5.8600786908
O            5.8603933036     8.2334734978     5.8000189482
O            3.1238100000     0.1116490000     1.9196440000    0   0   0
O            3.1238100000     5.6091080000     1.9196440000    0   0   0
O            8.5180770000     0.1116490000     1.9196440000    0   0   0
O            8.5180770000     5.6091080000     1.9196440000    0   0   0
O            1.1409624196     1.1636542304     3.5313236829
O            1.1448919210     6.6563121779     3.5516515470
O            6.5301127698     1.1675195278     3.5580082451
O            6.5440552939     6.6673536000     3.5446322324
O            1.1266440000     1.1517870000     0.3184580000    0   0   0
O            1.1522263159     1.0462625181     8.0595138513
O            1.1266440000     6.6492460000     0.3184580000    0   0   0
```

O	1.0701245235	6.5672072616	8.0421931533			
O	6.5209110000	1.1517870000	0.3184580000	0	0	0
O	6.5641563990	1.2201824495	8.0165984153			
O	6.5209110000	6.6492460000	0.3184580000	0	0	0
O	6.4758053216	6.6628805288	7.9105786549			
O	4.2811659271	4.3267403603	4.1918948197			
O	4.2449163233	9.8254209350	4.1640701711			
O	9.6541235147	4.3140156851	4.2113558613			
O	9.6349487081	9.8014463603	4.1874619339			
O	1.5764334859	3.8915672349	3.5804230847			
O	1.5626484739	9.4079176196	3.5624433965			
O	6.9745718446	3.8909697761	3.5604366267			
O	6.9573905908	9.4208451641	3.5294944778			
O	1.5704400000	3.9005160000	0.3184570000	0	0	0
O	1.6148600240	3.6719942023	8.2550944089			
O	1.5704400000	9.3979740000	0.3184570000	0	0	0
O	1.6858468565	9.2245524107	8.0340257791			
O	6.9647070000	3.9005160000	0.3184570000	0	0	0
O	7.0663239130	3.8866381803	8.3050053263			
O	6.9647070000	9.3979740000	0.3184570000	0	0	0
O	7.0285674919	9.4314499144	8.0869810831			
O	3.8410987795	1.5756596602	4.2284732561			
O	3.8713044678	7.0726595877	4.2173770976			
O	9.2530588680	1.5593428291	4.2137750487			
O	9.2336833972	7.0566060361	4.2193576202			
O	3.7769916985	1.6591682787	7.7867964419			
O	3.7555044265	7.2936153237	7.5969956722			
O	9.2960600479	1.4625147903	7.4300564082			
O	9.2650969425	6.9738520619	7.3687832040			
O	4.9675650000	2.8603790000	1.9196440000	0	0	0
O	4.9675650000	8.3578380000	1.9196440000	0	0	0
O	4.3290683950	4.2906412176	7.5132266223			
O	4.2995406552	9.8836897835	7.4241770109			
O	9.6341401111	4.1746428477	7.4290485829			
O	9.6651924219	9.7041575820	7.3865728769			
O	2.3166033364	5.3625572357	5.8533593042			
O	7.5721527891	5.3296144563	5.8191179818			
O	3.8237770000	1.5969400000	-0.3184580000	0	0	0
O	3.8237770000	7.0943990000	-0.3184580000	0	0	0
O	9.2180450000	1.5969400000	-0.3184580000	0	0	0
O	9.2180450000	7.0943990000	-0.3184580000	0	0	0
O	7.6240664590	-0.1024367735	5.8076092874			
O	-0.4267020000	2.8603790000	1.9196440000	0	0	0
O	-0.4267020000	8.3578380000	1.9196440000	0	0	0
O	4.2675730000	4.3456700000	-0.3184590000	0	0	0
O	4.2675730000	9.8431280000	-0.3184590000	0	0	0
O	9.6618410000	4.3456700000	-0.3184590000	0	0	0
O	9.6618410000	9.8431280000	-0.3184590000	0	0	0
O	2.2451399844	-0.1226370154	5.8313997028			
C	3.0912703019	5.7105049116	9.9459076999			
O	3.3987831892	5.9316582495	11.0410323566			
H	3.0621315180	2.2681627008	8.0956037226			
H	1.8072856810	3.4469818042	9.1828865274			
H	3.1081814129	7.9270798686	8.0174041779			
H	7.7574105524	3.2427498835	8.5632810905			

COH-c1.1

108						
COH_c1.1						
Ca	-0.1065928501	5.7719555017	5.7286959522			
Ca	2.8550429791	2.9545029593	5.7469080794			
Ca	2.8130862703	8.5624986408	5.7553442659			
Ca	8.1828496696	2.9425517082	5.7325682078			
Ca	8.2227595244	8.4867643111	5.7905732221			
Ca	2.6474700000	2.5105950000	1.9196440000	0	0	0
Ca	2.6474700000	8.0080540000	1.9196440000	0	0	0
Ca	8.0417380000	2.5105950000	1.9196440000	0	0	0
Ca	8.0417380000	8.0080540000	1.9196440000	0	0	0
Ca	5.2763166509	0.3061766719	5.7766102647			
Ca	5.3180335730	5.6825962839	5.7367296531			
Ca	10.6730827901	0.1870574230	5.7739150116			
Ca	0.0496100000	5.2593300000	1.9196450000	0	0	0
Ca	0.0496100000	10.7567880000	1.9196450000	0	0	0
Ca	5.4438780000	5.2593300000	1.9196450000	0	0	0
Ca	5.4438780000	10.7567880000	1.9196450000	0	0	0
Ti	0.0051732759	2.7250928717	3.7806270934			
Ti	0.0001199722	8.2339614123	3.7851534191			
Ti	5.4025451915	2.7429386365	3.7755559387			
Ti	5.4012283538	8.2238392518	3.8333522891			
Ti	0.0000000000	2.7487280000	0.0000000000	0	0	0
Ti	0.0656977992	2.6975845609	7.5450635446			
Ti	0.0000000000	8.2461860000	0.0000000000	0	0	0
Ti	0.1592153395	8.3809888223	7.5482763985			
Ti	5.3942680000	2.7487280000	0.0000000000	0	0	0
Ti	5.4869365227	2.9061993496	7.5807692329			
Ti	5.3942680000	8.2461860000	0.0000000000	0	0	0
Ti	5.6100626852	8.5459849246	7.6928956015			
Ti	2.7055657336	-0.0092582678	3.7764696664			
Ti	2.6948425050	5.4781078401	3.7964878907			
Ti	8.0972034866	-0.0213124472	3.7889249583			
Ti	8.1074239463	5.4764473829	3.7835925397			
Ti	2.6971090000	0.0000000000	0.0000000000	0	0	0
Ti	2.5867619659	0.1297541263	7.5512846220			
Ti	2.6971090000	5.4974580000	0.0000000000	0	0	0
Ti	2.3099270710	5.3313853448	7.6750119579			
Ti	8.0913760000	0.0000000000	0.0000000000	0	0	0
Ti	7.9640773039	0.1450931065	7.5622544482			
Ti	8.0913760000	5.4974580000	0.0000000000	0	0	0
Ti	8.1594467170	5.4777474220	7.5065112924			
O	0.5027855262	2.6246593303	5.7933788745			
O	0.5017640248	8.1551781663	5.7545817497			
O	5.8364513712	2.7346310903	5.7792809221			
O	5.8581293406	8.0519205280	5.8812479040			
O	3.1238100000	0.1116490000	1.9196440000	0	0	0
O	3.1238100000	5.6091080000	1.9196440000	0	0	0
O	8.5180770000	0.1116490000	1.9196440000	0	0	0
O	8.5180770000	5.6091080000	1.9196440000	0	0	0
O	1.1530479028	1.1771771945	3.5033566198			
O	1.1405272533	6.6596283312	3.5281887131			
O	6.5461411387	1.1825015181	3.5340237149			
O	6.5250518424	6.6577802541	3.5437980610			
O	1.1266440000	1.1517870000	0.3184580000	0	0	0
O	1.1153249986	1.1184920437	7.9962516475			
O	1.1266440000	6.6492460000	0.3184580000	0	0	0

O	1.0301399897	6.5864127542	7.9557929022			
O	6.5209110000	1.1517870000	0.3184580000	0	0	0
O	6.5563793847	1.2073920503	7.9481400596			
O	6.5209110000	6.6492460000	0.3184580000	0	0	0
O	6.5153109324	6.5795747919	8.1519179645			
O	4.2439857368	4.3274919929	4.1771444149			
O	4.2523396009	9.8123311888	4.2177259588			
O	9.6454409452	4.3103471596	4.1900674794			
O	9.6379939032	9.8101146221	4.1851242687			
O	1.5602524656	3.9173684396	3.5265514178			
O	1.5663445979	9.4199387329	3.5213123695			
O	6.9569280280	3.9207714919	3.5042469904			
O	6.9456147726	9.4064180238	3.5543839483			
O	1.5704400000	3.9005160000	0.3184570000	0	0	0
O	1.4421475989	3.8168175632	8.1943135025			
O	1.5704400000	9.3979740000	0.3184570000	0	0	0
O	1.6399230108	9.3567862370	7.9487467268			
O	6.9647070000	3.9005160000	0.3184570000	0	0	0
O	6.8659196384	3.9773286960	8.0665669317			
O	6.9647070000	9.3979740000	0.3184570000	0	0	0
O	7.0603887487	9.4689983444	8.1225805151			
O	3.8624181831	1.5701769727	4.1765192313			
O	3.8328281544	7.0573627178	4.2684046431			
O	9.2582317707	1.5568720455	4.1971846880			
O	9.2411799513	7.0661846272	4.1919278941			
O	3.8688272251	1.4842577411	7.3902642540			
O	3.6939190744	6.8850879777	8.0095129666			
O	9.3021678485	1.4909113927	7.3737509269			
O	9.2100124250	6.9612352722	7.3411643675			
O	4.9675650000	2.8603790000	1.9196440000	0	0	0
O	4.9675650000	8.3578380000	1.9196440000	0	0	0
O	4.1532023923	4.2663686012	7.4246061364			
O	4.2701013149	9.7559213458	7.3439166694			
O	9.5615967801	4.2029628838	7.3471004393			
O	9.6500842631	9.7091015227	7.3622931572			
O	2.1973476842	5.3218613863	5.8439753312			
O	7.6266167810	5.3491549129	5.7671907068			
O	3.8237770000	1.5969400000	-0.3184580000	0	0	0
O	3.8237770000	7.0943990000	-0.3184580000	0	0	0
O	9.2180450000	1.5969400000	-0.3184580000	0	0	0
O	9.2180450000	7.0943990000	-0.3184580000	0	0	0
O	7.6150186232	-0.0907708823	5.7831173268			
O	-0.4267020000	2.8603790000	1.9196440000	0	0	0
O	-0.4267020000	8.3578380000	1.9196440000	0	0	0
O	4.2675730000	4.3456700000	-0.3184590000	0	0	0
O	4.2675730000	9.8431280000	-0.3184590000	0	0	0
O	9.6618410000	4.3456700000	-0.3184590000	0	0	0
O	9.6618410000	9.8431280000	-0.3184590000	0	0	0
O	2.2295216711	-0.0681241374	5.7650995491			
C	4.1794580815	7.5272389683	8.9914112339			
O	3.7019509571	7.1461121902	10.1845507940			
H	4.1511321005	7.6724982871	10.8746924246			
H	5.9690141297	5.8809564503	8.5651886275			

CHO-c1.1

108						
CHO_c1.1						
Ca	-0.1108545418	5.7319024860	5.8095816192			
Ca	2.8162532278	2.9729896216	5.7672432688			
Ca	2.7528111011	8.5868857476	5.7070374456			
Ca	8.1831492404	2.9834066036	5.7406300472			
Ca	8.2017964678	8.4320516729	5.7824715707			
Ca	2.6474700000	2.5105950000	1.9196440000	0	0	0
Ca	2.6474700000	8.0080540000	1.9196440000	0	0	0
Ca	8.0417380000	2.5105950000	1.9196440000	0	0	0
Ca	8.0417380000	8.0080540000	1.9196440000	0	0	0
Ca	5.2502764143	0.2363585323	5.7683981811			
Ca	5.3577372565	5.7278505537	5.6344344405			
Ca	10.6946738523	0.1838958280	5.7724648277			
Ca	0.0496100000	5.2593300000	1.9196450000	0	0	0
Ca	0.0496100000	10.7567880000	1.9196450000	0	0	0
Ca	5.4438780000	5.2593300000	1.9196450000	0	0	0
Ca	5.4438780000	10.7567880000	1.9196450000	0	0	0
Ti	-0.0057905636	2.7258064917	3.7873873243			
Ti	-0.0130283689	8.2191066996	3.7883074574			
Ti	5.3958346244	2.7252692784	3.7804514660			
Ti	5.3895244471	8.2468458013	3.8059810243			
Ti	0.0000000000	2.7487280000	0.0000000000	0	0	0
Ti	-0.0341408030	2.7401498170	7.5170640288			
Ti	0.0000000000	8.2461860000	0.0000000000	0	0	0
Ti	0.1296811461	8.3530061788	7.5667917992			
Ti	5.3942680000	2.7487280000	0.0000000000	0	0	0
Ti	5.6331118705	2.6788924723	7.6309875283			
Ti	5.3942680000	8.2461860000	0.0000000000	0	0	0
Ti	5.5666930814	8.4490276717	7.6392672697			
Ti	2.6952824593	0.0082790743	3.7746724864			
Ti	2.6964000699	5.4855535237	3.8364013705			
Ti	8.1004905663	-0.0265500246	3.7843994535			
Ti	8.1099875761	5.4716054043	3.7825487212			
Ti	2.6971090000	0.0000000000	0.0000000000	0	0	0
Ti	2.5740190403	0.1466648281	7.5636994550			
Ti	2.6971090000	5.4974580000	0.0000000000	0	0	0
Ti	2.2968270428	5.3457733573	7.8603048591			
Ti	8.0913760000	0.0000000000	0.0000000000	0	0	0
Ti	8.0303540837	0.0217666552	7.5332901729			
Ti	8.0913760000	5.4974580000	0.0000000000	0	0	0
Ti	7.9284109952	5.5904071510	7.5406862621			
O	0.4759419598	2.6132857370	5.7743402011			
O	0.4462477485	8.1399989763	5.7755842785			
O	5.8433900649	2.6575046383	5.8138013546			
O	5.8109216179	8.1274529025	5.8077455297			
O	3.1238100000	0.1116490000	1.9196440000	0	0	0
O	3.1238100000	5.6091080000	1.9196440000	0	0	0
O	8.5180770000	0.1116490000	1.9196440000	0	0	0
O	8.5180770000	5.6091080000	1.9196440000	0	0	0
O	1.1399124798	1.1751850230	3.4888475504			
O	1.1367650783	6.6533949203	3.5580122104			
O	6.5344357903	1.1691624560	3.5341686330			
O	6.5164795551	6.6568205279	3.5433001436			
O	1.1266440000	1.1517870000	0.3184580000	0	0	0
O	1.1291426487	1.1208543013	7.9879829213			
O	1.1266440000	6.6492460000	0.3184580000	0	0	0

O	1.0416519091	6.6359802205	8.0003498951			
O	6.5209110000	1.1517870000	0.3184580000	0	0	0
O	6.5614882647	1.1105826185	8.0110254738			
O	6.5209110000	6.6492460000	0.3184580000	0	0	0
O	6.3924457688	6.5593686212	7.8527072402			
O	4.2464852170	4.3138151183	4.1935902146			
O	4.2411125744	9.8284036814	4.1772607440			
O	9.6464211195	4.3136042076	4.1859412237			
O	9.6304455371	9.8047355598	4.1920850573			
O	1.5630230220	3.9203030787	3.5715787779			
O	1.5590990763	9.4149344943	3.5520484698			
O	6.9496807312	3.9110317024	3.5180809385			
O	6.9448872020	9.4084331169	3.5255221382			
O	1.5704400000	3.9005160000	0.3184570000	0	0	0
O	1.4819650726	3.7952672120	8.1111044862			
O	1.5704400000	9.3979740000	0.3184570000	0	0	0
O	1.5639156680	9.3903533280	7.9223550317			
O	6.9647070000	3.9005160000	0.3184570000	0	0	0
O	6.9410975876	3.8677580473	8.0426168088			
O	6.9647070000	9.3979740000	0.3184570000	0	0	0
O	6.9926570339	9.3949062515	8.0907164999			
O	3.8409635499	1.5835924226	4.1865418997			
O	3.8273069475	7.0765121373	4.2320586587			
O	9.2417741984	1.5559779423	4.2005925334			
O	9.2320073401	7.0584038260	4.1884074176			
O	3.8787386993	1.5487970898	7.4163912655			
O	4.0728905009	7.4709834446	8.6972412280			
O	9.2816450742	1.4444660232	7.3490197439			
O	9.1993555606	6.9308033201	7.3859658878			
O	4.9675650000	2.8603790000	1.9196440000	0	0	0
O	4.9675650000	8.3578380000	1.9196440000	0	0	0
O	4.3190894489	4.4857338020	7.6278589201			
O	4.2266801352	9.7190376349	7.3016431927			
O	9.5912991804	4.1641591659	7.3454544943			
O	9.5723174453	9.7091368032	7.3811790042			
O	2.3268856487	5.3819797894	5.9475717428			
O	7.6219738432	5.3430529479	5.7462117580			
O	3.8237770000	1.5969400000	-0.3184580000	0	0	0
O	3.8237770000	7.0943990000	-0.3184580000	0	0	0
O	9.2180450000	1.5969400000	-0.3184580000	0	0	0
O	9.2180450000	7.0943990000	-0.3184580000	0	0	0
O	7.5986505452	-0.1412685233	5.7841786485			
O	-0.4267020000	2.8603790000	1.9196440000	0	0	0
O	-0.4267020000	8.3578380000	1.9196440000	0	0	0
O	4.2675730000	4.3456700000	-0.3184590000	0	0	0
O	4.2675730000	9.8431280000	-0.3184590000	0	0	0
O	9.6618410000	4.3456700000	-0.3184590000	0	0	0
O	9.6618410000	9.8431280000	-0.3184590000	0	0	0
O	2.2403350552	-0.0361901911	5.7605923574			
C	3.8100582504	6.8316280448	9.7675149349			
O	3.0709517711	5.8107268496	9.7843437679			
H	4.2446956223	7.1869235971	10.7188698625			
H	4.9736416261	5.0621342594	8.0957051394			

CHO-c1.2

108
CHO_c1.2

Ca	-0.0216942645	5.7586469853	5.7564541659			
Ca	2.8495418778	2.9893992583	5.7647720981			
Ca	2.7852250924	8.5677850182	5.7222522434			
Ca	8.1777575723	2.9545780701	5.7217541058			
Ca	8.2204317461	8.4628779634	5.8058279799			
Ca	2.6474700000	2.5105950000	1.9196440000	0	0	0
Ca	2.6474700000	8.0080540000	1.9196440000	0	0	0
Ca	8.0417380000	2.5105950000	1.9196440000	0	0	0
Ca	8.0417380000	8.0080540000	1.9196440000	0	0	0
Ca	5.2631652324	0.3138912848	5.7875918840			
Ca	5.3385665685	5.6325278065	5.6687845514			
Ca	10.6703584590	0.1607816236	5.7609377101			
Ca	0.0496100000	5.2593300000	1.9196450000	0	0	0
Ca	0.0496100000	10.7567880000	1.9196450000	0	0	0
Ca	5.4438780000	5.2593300000	1.9196450000	0	0	0
Ca	5.4438780000	10.7567880000	1.9196450000	0	0	0
Ti	0.0060231635	2.7205280766	3.7799451524			
Ti	-0.0050353284	8.2301983593	3.7861798696			
Ti	5.4019465842	2.7378440018	3.7715653243			
Ti	5.3905522904	8.2228292504	3.8011363644			
Ti	0.0000000000	2.7487280000	0.0000000000	0	0	0
Ti	0.0287645867	2.6602449174	7.5207854593			
Ti	0.0000000000	8.2461860000	0.0000000000	0	0	0
Ti	0.1645846453	8.3851768793	7.5550499653			
Ti	5.3942680000	2.7487280000	0.0000000000	0	0	0
Ti	5.5055266885	2.9135351680	7.5883332694			
Ti	5.3942680000	8.2461860000	0.0000000000	0	0	0
Ti	5.5671851873	8.4938697852	7.6583566304			
Ti	2.7079010973	-0.0005003694	3.7773258601			
Ti	2.6941453619	5.4835512161	3.8365361340			
Ti	8.0929278311	-0.0250625849	3.7880423162			
Ti	8.1289107397	5.4792991039	3.7900058973			
Ti	2.6971090000	0.0000000000	0.0000000000	0	0	0
Ti	2.5829522193	0.1391421724	7.5546328729			
Ti	2.6971090000	5.4974580000	0.0000000000	0	0	0
Ti	2.3754021515	5.2983284326	7.7985428938			
Ti	8.0913760000	0.0000000000	0.0000000000	0	0	0
Ti	7.9474515490	0.1134018996	7.5522581264			
Ti	8.0913760000	5.4974580000	0.0000000000	0	0	0
Ti	8.1852542178	5.4858076735	7.5172661088			
O	0.4932160597	2.5981880302	5.7803787592			
O	0.4877969016	8.1795999673	5.7554564685			
O	5.8357320719	2.7404849687	5.7827614889			
O	5.8262816738	8.0113987754	5.9008351442			
O	3.1238100000	0.1116490000	1.9196440000	0	0	0
O	3.1238100000	5.6091080000	1.9196440000	0	0	0
O	8.5180770000	0.1116490000	1.9196440000	0	0	0
O	8.5180770000	5.6091080000	1.9196440000	0	0	0
O	1.1526603112	1.1744196040	3.4915426268			
O	1.1353361189	6.6577828620	3.5462727936			
O	6.5424060835	1.1792653286	3.5301880704			
O	6.5281540850	6.6595902373	3.5497931700			
O	1.1266440000	1.1517870000	0.3184580000	0	0	0
O	1.1114099503	1.1336944643	8.0077676945			
O	1.1266440000	6.6492460000	0.3184580000	0	0	0

O	1.1534865024	6.6414918150	7.9193131389			
O	6.5209110000	1.1517870000	0.3184580000	0	0	0
O	6.5553354940	1.1941299172	7.9473561212			
O	6.5209110000	6.6492460000	0.3184580000	0	0	0
O	6.5835295030	6.5931198101	8.2095659709			
O	4.2541446587	4.3205142429	4.1448039036			
O	4.2587426957	9.8195961592	4.2177523637			
O	9.6553358417	4.3169660204	4.2001196666			
O	9.6297833387	9.8049841151	4.1805056267			
O	1.5590692883	3.9180465594	3.5538805520			
O	1.5699677850	9.4195128804	3.5328065736			
O	6.9654971834	3.9206151855	3.5265186586			
O	6.9423319002	9.4016067277	3.5536686845			
O	1.5704400000	3.9005160000	0.3184570000	0	0	0
O	1.4456789284	3.8183365288	8.1020519255			
O	1.5704400000	9.3979740000	0.3184570000	0	0	0
O	1.6186027946	9.3992593230	7.9496188344			
O	6.9647070000	3.9005160000	0.3184570000	0	0	0
O	6.9007336729	3.9619830553	8.0543065556			
O	6.9647070000	9.3979740000	0.3184570000	0	0	0
O	7.0193522814	9.4309697615	8.0728783504			
O	3.8562177447	1.5791972762	4.1861136916			
O	3.8404002788	7.0759067252	4.2305257306			
O	9.2520631614	1.5540665135	4.1937025863			
O	9.2459648356	7.0670640922	4.1969742405			
O	3.8671299714	1.5059427439	7.4042276967			
O	4.0874842552	7.3563468197	8.5600471408			
O	9.2888032032	1.4737934033	7.3448436422			
O	9.2695378014	6.9498638455	7.3648880799			
O	4.9675650000	2.8603790000	1.9196440000	0	0	0
O	4.9675650000	8.3578380000	1.9196440000	0	0	0
O	4.2135700376	4.3319223068	7.4249838841			
O	4.2587291725	9.7425926198	7.3234968578			
O	9.5760052491	4.1936364190	7.3454985046			
O	9.6296284320	9.7043826352	7.3707599357			
O	2.3145397541	5.3747975439	5.8583260011			
O	7.6440503778	5.3783378837	5.7802569831			
O	3.8237770000	1.5969400000	-0.3184580000	0	0	0
O	3.8237770000	7.0943990000	-0.3184580000	0	0	0
O	9.2180450000	1.5969400000	-0.3184580000	0	0	0
O	9.2180450000	7.0943990000	-0.3184580000	0	0	0
O	7.6009754474	-0.1077968153	5.7753920931			
O	-0.4267020000	2.8603790000	1.9196440000	0	0	0
O	-0.4267020000	8.3578380000	1.9196440000	0	0	0
O	4.2675730000	4.3456700000	-0.3184590000	0	0	0
O	4.2675730000	9.8431280000	-0.3184590000	0	0	0
O	9.6618410000	4.3456700000	-0.3184590000	0	0	0
O	9.6618410000	9.8431280000	-0.3184590000	0	0	0
O	2.2308179025	-0.0546777457	5.7675462663			
C	3.8497227868	6.7658157085	9.6732567121			
O	3.1381980724	5.7346449520	9.7457208850			
H	4.2963008455	7.1802613431	10.5949564516			
H	5.9945938474	5.8874182512	8.5472735041			

*CHO-c2.1

109						
CHO_c2.1						
Ca	-0.0849147753	5.7014042278	5.9071652475			
Ca	2.8130917523	2.9447372554	5.7814465851			
Ca	2.8174451742	8.5173542748	5.7482336811			
Ca	8.2070481433	3.0002863470	5.7817322017			
Ca	8.2102906863	8.4726316492	5.7985541526			
Ca	2.6474700000	2.5105950000	1.9196440000	0	0	0
Ca	2.6474700000	8.0080540000	1.9196440000	0	0	0
Ca	8.0417380000	2.5105950000	1.9196440000	0	0	0
Ca	8.0417380000	8.0080540000	1.9196440000	0	0	0
Ca	5.2445701531	0.2319520616	5.8058819669			
Ca	5.2541100157	5.7481012578	5.8106016391			
Ca	10.6843496622	0.2286252335	5.7786539017			
Ca	0.0496100000	5.2593300000	1.9196450000	0	0	0
Ca	0.0496100000	10.7567880000	1.9196450000	0	0	0
Ca	5.4438780000	5.2593300000	1.9196450000	0	0	0
Ca	5.4438780000	10.7567880000	1.9196450000	0	0	0
Ti	-0.0000575607	2.7340524992	3.7924871134			
Ti	-0.0047499234	8.1994765401	3.7941897071			
Ti	5.3985978788	2.7341005871	3.7932463205			
Ti	5.3994445206	8.2231453159	3.7922368816			
Ti	0.0000000000	2.7487280000	0.0000000000	0	0	0
Ti	0.0147698514	2.7728281141	7.5359113874			
Ti	0.0000000000	8.2461860000	0.0000000000	0	0	0
Ti	0.1088835252	8.4011852307	7.5346311440			
Ti	5.3942680000	2.7487280000	0.0000000000	0	0	0
Ti	5.5843295409	2.7813532800	7.5756957977			
Ti	5.3942680000	8.2461860000	0.0000000000	0	0	0
Ti	5.5669901299	8.4194814449	7.5814167035			
Ti	2.6950830009	-0.0190919265	3.7918713874			
Ti	2.6730155974	5.4781518441	3.9060113756			
Ti	8.0923385371	-0.0191866148	3.7952043897			
Ti	8.1010536865	5.4774526997	3.8053441751			
Ti	2.6971090000	0.0000000000	0.0000000000	0	0	0
Ti	2.5799744080	0.1228637497	7.5620913837			
Ti	2.6971090000	5.4974580000	0.0000000000	0	0	0
Ti	2.6716324257	5.2343888189	7.7965434041			
Ti	8.0913760000	0.0000000000	0.0000000000	0	0	0
Ti	7.9720615376	0.0949382118	7.5434130194			
Ti	8.0913760000	5.4974580000	0.0000000000	0	0	0
Ti	7.9205049614	5.6364518723	7.5475156121			
O	0.4637786884	2.6349578447	5.7667967707			
O	0.4957534152	8.1091754564	5.7747694929			
O	5.8815214202	2.6317729261	5.7749947814			
O	5.8454719417	8.1656442056	5.7764790497			
O	3.1238100000	0.1116490000	1.9196440000	0	0	0
O	3.1238100000	5.6091080000	1.9196440000	0	0	0
O	8.5180770000	0.1116490000	1.9196440000	0	0	0
O	8.5180770000	5.6091080000	1.9196440000	0	0	0
O	1.1409169778	1.1829223519	3.5019288811			
O	1.1393308448	6.6838523293	3.5009493773			
O	6.5424678889	1.1842753541	3.4947190058			
O	6.5400723109	6.6862110042	3.5050426582			
O	1.1266440000	1.1517870000	0.3184580000	0	0	0
O	1.1240458827	1.1042435744	7.9662319391			
O	1.1266440000	6.6492460000	0.3184580000	0	0	0

O	1.0108847802	6.5907235955	8.0740521923			
O	6.5209110000	1.1517870000	0.3184580000	0	0	0
O	6.5477084328	1.1537654660	8.0015728788			
O	6.5209110000	6.6492460000	0.3184580000	0	0	0
O	6.4515227125	6.6453678682	7.9192436644			
O	4.2552434656	4.3116785564	4.2079017012			
O	4.2402997415	9.8222011006	4.1799203444			
O	9.6415321940	4.3271840380	4.2077259854			
O	9.6305316794	9.8124279304	4.1989466751			
O	1.5530844601	3.9195338615	3.5250186294			
O	1.5506918736	9.4247533609	3.5187504698			
O	6.9547253252	3.9270766284	3.5014826705			
O	6.9479017381	9.4315530459	3.5039925063			
O	1.5704400000	3.9005160000	0.3184570000	0	0	0
O	1.5398835757	3.8179919345	8.0168940705			
O	1.5704400000	9.3979740000	0.3184570000	0	0	0
O	1.6102242762	9.3307519862	7.9588900304			
O	6.9647070000	3.9005160000	0.3184570000	0	0	0
O	6.9253409706	3.9031835905	7.9838294924			
O	6.9647070000	9.3979740000	0.3184570000	0	0	0
O	7.0081035044	9.4179606597	8.0075922415			
O	3.8514351010	1.5725695059	4.1851034902			
O	3.8620724068	7.0746019584	4.2037078101			
O	9.2399371880	1.5717188038	4.1898731376			
O	9.2395703433	7.0715648462	4.1949000373			
O	3.8757810030	1.4802245122	7.3837864328			
O	3.7537449310	7.1247997045	7.6706171256			
O	9.2881342486	1.4617244203	7.3662331051			
O	9.2182950273	6.9563486187	7.3723331275			
O	4.9675650000	2.8603790000	1.9196440000	0	0	0
O	4.9675650000	8.3578380000	1.9196440000	0	0	0
O	4.2108811732	4.2498360238	7.3946478699			
O	4.2410272339	9.7299114848	7.3884028728			
O	9.5983150934	4.1767485775	7.3765927358			
O	9.6218153750	9.7286855864	7.3471200664			
O	2.2601924260	5.4358454124	5.7925178875			
O	7.5781479223	5.3935712639	5.7502972337			
O	3.8237770000	1.5969400000	-0.3184580000	0	0	0
O	3.8237770000	7.0943990000	-0.3184580000	0	0	0
O	9.2180450000	1.5969400000	-0.3184580000	0	0	0
O	9.2180450000	7.0943990000	-0.3184580000	0	0	0
O	7.5969448055	-0.0880520458	5.7645799428			
O	-0.4267020000	2.8603790000	1.9196440000	0	0	0
O	-0.4267020000	8.3578380000	1.9196440000	0	0	0
O	4.2675730000	4.3456700000	-0.3184590000	0	0	0
O	4.2675730000	9.8431280000	-0.3184590000	0	0	0
O	9.6618410000	4.3456700000	-0.3184590000	0	0	0
O	9.6618410000	9.8431280000	-0.3184590000	0	0	0
O	2.2345125968	-0.0880608402	5.7546712498			
C	2.7210959102	5.6309329091	9.9832116082			
O	1.7995682621	6.1736970031	10.5976401735			
H	3.6146016229	5.3126905562	10.6051477900			
H	0.8979364886	6.4388280998	9.0545691270			
H	3.0948505989	7.7126945993	8.1131405188			

*CHO-c2.2

```
108
CHO_c2.2
Ca             -0.0693459481         5.7171927371         5.8675522389
Ca              2.7964724210         2.9165828348         5.9141320352
Ca              2.8186930827         8.4296398689         5.9560826747
Ca              8.1496299016         2.9787709934         5.8426664330
Ca              8.2655876701         8.4814666288         5.8375078372
Ca              2.6474700000         2.5105950000         1.9196440000    0    0    0
Ca              2.6474700000         8.0080540000         1.9196440000    0    0    0
Ca              8.0417380000         2.5105950000         1.9196440000    0    0    0
Ca              8.0417380000         8.0080540000         1.9196440000    0    0    0
Ca              5.2713209653         0.2305251527         5.8586274361
Ca              5.1983031133         5.6667350214         5.9617484718
Ca             10.7094167839         0.1923175563         5.8498750153
Ca              0.0496100000         5.2593300000         1.9196450000    0    0    0
Ca              0.0496100000        10.7567880000         1.9196450000    0    0    0
Ca              5.4438780000         5.2593300000         1.9196450000    0    0    0
Ca              5.4438780000        10.7567880000         1.9196450000    0    0    0
Ti             -0.0209255902         2.6471681558         3.7961163516
Ti             -0.0035829828         8.1449466786         3.8011661298
Ti              5.3596706306         2.6608585652         3.8070744060
Ti              5.3836648660         8.1302419703         3.8070661153
Ti              0.0000000000         2.7487280000         0.0000000000    0    0    0
Ti             -0.0233995718         2.7953657764         7.5225203666
Ti              0.0000000000         8.2461860000         0.0000000000    0    0    0
Ti              0.1308062049         8.3630481211         7.5622988723
Ti              5.3942680000         2.7487280000         0.0000000000    0    0    0
Ti              5.5016125245         2.8513607255         7.5715826887
Ti              5.3942680000         8.2461860000         0.0000000000    0    0    0
Ti              5.6335051832         8.4245428223         7.5469157795
Ti              2.7225430349        -0.1182139902         3.8088243546
Ti              2.6957935551         5.3806672994         3.8946470177
Ti              8.0944982544        -0.0916700991         3.7960789534
Ti              8.0988774995         5.3998027207         3.8081119048
Ti              2.6971090000         0.0000000000         0.0000000000    0    0    0
Ti              2.5784340738         0.0987610333         7.5732611380
Ti              2.6971090000         5.4974580000         0.0000000000    0    0    0
Ti              2.3799530258         5.3800335668         7.8304543505
Ti              8.0913760000         0.0000000000         0.0000000000    0    0    0
Ti              7.9696896953         0.1088967785         7.5559533528
Ti              8.0913760000         5.4974580000         0.0000000000    0    0    0
Ti              7.9519395038         5.6376799476         7.5340533829
O               0.4488956030         2.6686769277         5.7516803746
O               0.4830967582         8.1695740340         5.7459721006
O               5.8411465893         2.6430865755         5.7459747672
O               5.9161054844         8.1692305784         5.7489789279
O               3.1238100000         0.1116490000         1.9196440000    0    0    0
O               3.1238100000         5.6091080000         1.9196440000    0    0    0
O               8.5180770000         0.1116490000         1.9196440000    0    0    0
O               8.5180770000         5.6091080000         1.9196440000    0    0    0
O               1.1221328460         1.1989782527         3.5070334586
O               1.1411337797         6.6961475865         3.5115006181
O               6.5275966527         1.2072682577         3.4877135259
O               6.5405074841         6.7161786674         3.4553892832
O               1.1266440000         1.1517870000         0.3184580000    0    0    0
O               1.1536435488         1.1286077066         7.9056147323
O               1.1266440000         6.6492460000         0.3184580000    0    0    0
```

O	1.0884312534	6.6337167032	7.9565047859			
O	6.5209110000	1.1517870000	0.3184580000	0	0	0
O	6.5734648804	1.1576591960	7.9683180091			
O	6.5209110000	6.6492460000	0.3184580000	0	0	0
O	6.5342085645	6.6797387403	8.0015281225			
O	4.2881476371	4.3791360783	4.1612355055			
O	4.2725240479	9.8832223316	4.1629379571			
O	9.6467461786	4.3973984782	4.1878959071			
O	9.6517573698	9.8866149458	4.1761567091			
O	1.5539439922	3.9453058224	3.5013993253			
O	1.5580048954	9.4535928070	3.4727631414			
O	6.9698600530	3.9460276531	3.4935650716			
O	6.9620615839	9.4580272834	3.4872945273			
O	1.5704400000	3.9005160000	0.3184570000	0	0	0
O	1.4648858907	3.8142123942	8.0694346645			
O	1.5704400000	9.3979740000	0.3184570000	0	0	0
O	1.5998448921	9.3632331242	7.9524062752			
O	6.9647070000	3.9005160000	0.3184570000	0	0	0
O	6.9227711428	3.9235005688	7.9190267198			
O	6.9647070000	9.3979740000	0.3184570000	0	0	0
O	7.0610934029	9.4231832104	7.9930439337			
O	3.8198616610	1.6351175086	4.1750815797			
O	3.8519722383	7.1380725484	4.2258657962			
O	9.2144068593	1.6501052979	4.1729367593			
O	9.2383893782	7.1360233862	4.1688212439			
O	3.9043905213	1.4147042086	7.3989244401			
O	3.9317479540	7.0063309958	7.4802957639			
O	9.3093554875	1.4724755557	7.3785831458			
O	9.2676153531	6.9497510576	7.3391836981			
O	4.9675650000	2.8603790000	1.9196440000	0	0	0
O	4.9675650000	8.3578380000	1.9196440000	0	0	0
O	4.1712713164	4.1313288110	7.3940135242			
O	4.2833745196	9.6911901806	7.3901643085			
O	9.5716311911	4.2042268735	7.3400334432			
O	9.6435492648	9.7156205005	7.3676715134			
O	2.3072627930	5.3664633598	5.7830409527			
O	7.5618708770	5.4358565293	5.7322263034			
O	3.8237770000	1.5969400000	-0.3184580000	0	0	0
O	3.8237770000	7.0943990000	-0.3184580000	0	0	0
O	9.2180450000	1.5969400000	-0.3184580000	0	0	0
O	9.2180450000	7.0943990000	-0.3184580000	0	0	0
O	7.6242977406	-0.0746975787	5.7508508316			
O	-0.4267020000	2.8603790000	1.9196440000	0	0	0
O	-0.4267020000	8.3578380000	1.9196440000	0	0	0
O	4.2675730000	4.3456700000	-0.3184590000	0	0	0
O	4.2675730000	9.8431280000	-0.3184590000	0	0	0
O	9.6618410000	4.3456700000	-0.3184590000	0	0	0
O	9.6618410000	9.8431280000	-0.3184590000	0	0	0
O	2.2579083176	-0.1207979259	5.7463700367			
C	3.4314802741	5.4206281327	9.8333759453			
O	2.8306438954	5.7578226641	10.8409994883			
H	4.4561339934	4.9378776082	9.9533821059			
H	3.9251180611	6.8271191147	8.4538004802			

*CHO-c3

109
CHO_c3

Ca	-0.0538530322	5.6856583494	5.8580999417			
Ca	2.8081705010	3.0499496216	5.6744225425			
Ca	2.8206463697	8.4518106212	5.8046768446			
Ca	8.1356664380	2.9862941742	5.8027364125			
Ca	8.2238097782	8.4660607910	5.8077483179			
Ca	2.6474700000	2.5105950000	1.9196440000	0	0	0
Ca	2.6474700000	8.0080540000	1.9196440000	0	0	0
Ca	8.0417380000	2.5105950000	1.9196440000	0	0	0
Ca	8.0417380000	8.0080540000	1.9196440000	0	0	0
Ca	5.2247079283	0.2976040707	5.8474612435			
Ca	5.3036809292	5.7678683645	5.7167573277			
Ca	10.6405338658	0.1710599131	5.7467317970			
Ca	0.0496100000	5.2593300000	1.9196450000	0	0	0
Ca	0.0496100000	10.7567880000	1.9196450000	0	0	0
Ca	5.4438780000	5.2593300000	1.9196450000	0	0	0
Ca	5.4438780000	10.7567880000	1.9196450000	0	0	0
Ti	-0.0090735452	2.7208744057	3.7934360053			
Ti	-0.0033698282	8.2134885559	3.7981586146			
Ti	5.4136088349	2.7443710900	3.8556123292			
Ti	5.4018828725	8.2457695258	3.7885715823			
Ti	0.0000000000	2.7487280000	0.0000000000	0	0	0
Ti	-0.0575071393	2.6864876121	7.5096741117			
Ti	0.0000000000	8.2461860000	0.0000000000	0	0	0
Ti	0.0599373228	8.2832577473	7.5424031626			
Ti	5.3942680000	2.7487280000	0.0000000000	0	0	0
Ti	5.5061526202	3.0127226941	7.6847641509			
Ti	5.3942680000	8.2461860000	0.0000000000	0	0	0
Ti	5.5090329139	8.4025058564	7.5444877410			
Ti	2.6985178048	-0.0279731797	3.8045794383			
Ti	2.6835217818	5.4965287459	3.8530028956			
Ti	8.0606987977	-0.0125189474	3.7938656863			
Ti	8.1179484222	5.4793205033	3.8085078338			
Ti	2.6971090000	0.0000000000	0.0000000000	0	0	0
Ti	2.4261885530	-0.0980534798	7.6411224787			
Ti	2.6971090000	5.4974580000	0.0000000000	0	0	0
Ti	2.7386705017	5.6725466663	7.6784947822			
Ti	8.0913760000	0.0000000000	0.0000000000	0	0	0
Ti	7.8736286405	0.0746726921	7.5585657299			
Ti	8.0913760000	5.4974580000	0.0000000000	0	0	0
Ti	7.9644671416	5.6489657751	7.5673871864			
O	0.4830370364	2.5972464202	5.7804738929			
O	0.4885912409	8.1256665278	5.7631509667			
O	5.8339472150	2.6147889165	5.7637769547			
O	5.8842280866	8.2140034405	5.7643226776			
O	3.1238100000	0.1116490000	1.9196440000	0	0	0
O	3.1238100000	5.6091080000	1.9196440000	0	0	0
O	8.5180770000	0.1116490000	1.9196440000	0	0	0
O	8.5180770000	5.6091080000	1.9196440000	0	0	0
O	1.1339097462	1.1664306667	3.5163318316			
O	1.1408447481	6.6775995168	3.4892561296			
O	6.5426541682	1.1757108467	3.4802711802			
O	6.5380784567	6.6833684632	3.5245742129			
O	1.1266440000	1.1517870000	0.3184580000	0	0	0
O	1.1407139829	1.0935803238	8.0862928853			
O	1.1266440000	6.6492460000	0.3184580000	0	0	0

O	1.2238654746	6.6027250173	7.9856958369			
O	6.5209110000	1.1517870000	0.3184580000	0	0	0
O	6.5270350879	1.2010301772	7.9087517632			
O	6.5209110000	6.6492460000	0.3184580000	0	0	0
O	6.5972521921	6.7250413445	7.9765246086			
O	4.2573222564	4.3276761597	4.1226972429			
O	4.2418849595	9.8311060821	4.2021867074			
O	9.6503223078	4.3174429784	4.2053716921			
O	9.6183847386	9.7961711470	4.1809681735			
O	1.5527462938	3.9070889035	3.5340425330			
O	1.5514978052	9.4247726478	3.5139227132			
O	6.9707548227	3.9215806627	3.5416932537			
O	6.9388974156	9.4300339312	3.4716103177			
O	1.5704400000	3.9005160000	0.3184570000	0	0	0
O	1.6177231231	3.7366029635	8.1282006423			
O	1.5704400000	9.3979740000	0.3184570000	0	0	0
O	1.5580295260	9.3189519179	8.0608452049			
O	6.9647070000	3.9005160000	0.3184570000	0	0	0
O	7.0047664986	3.9623896089	7.9603415384			
O	6.9647070000	9.3979740000	0.3184570000	0	0	0
O	6.9603529958	9.4456932029	8.0221958495			
O	3.8361833173	1.5579074450	4.2092096513			
O	3.8490666829	7.0838937029	4.1962379898			
O	9.2294664001	1.5681192168	4.2034103085			
O	9.2509753584	7.0654053540	4.1893378072			
O	3.7760657481	1.5689757175	7.6714459017			
O	3.9639178321	7.0216211786	7.3783002883			
O	9.3025385773	1.4391366010	7.3646328208			
O	9.3527267428	6.9425665891	7.4026217542			
O	4.9675650000	2.8603790000	1.9196440000	0	0	0
O	4.9675650000	8.3578380000	1.9196440000	0	0	0
O	4.2607597797	4.2246670300	7.0517423804			
O	4.2130267235	9.7688504392	7.3986688646			
O	9.6663612756	4.1696301313	7.3806267155			
O	9.6339284303	9.6682293795	7.3416229308			
O	2.2451657913	5.3674783796	5.7663955340			
O	7.6430780958	5.4400664707	5.7677371532			
O	3.8237770000	1.5969400000	-0.3184580000	0	0	0
O	3.8237770000	7.0943990000	-0.3184580000	0	0	0
O	9.2180450000	1.5969400000	-0.3184580000	0	0	0
O	9.2180450000	7.0943990000	-0.3184580000	0	0	0
O	7.5770123374	-0.1189217259	5.7553420782			
O	-0.4267020000	2.8603790000	1.9196440000	0	0	0
O	-0.4267020000	8.3578380000	1.9196440000	0	0	0
O	4.2675730000	4.3456700000	-0.3184590000	0	0	0
O	4.2675730000	9.8431280000	-0.3184590000	0	0	0
O	9.6618410000	4.3456700000	-0.3184590000	0	0	0
O	9.6618410000	9.8431280000	-0.3184590000	0	0	0
O	2.2007479518	-0.1372041965	5.8156406142			
C	3.7979405673	4.7890749767	9.5592730162			
O	4.6003867720	3.8250773152	9.5532498038			
H	3.5982690272	5.1500092642	10.6097860703			
H	3.0954995264	2.1852244865	8.0234396184			
H	1.9385663307	3.3889115889	8.9797323976			

*CHO-c4

```
109
CHO_c4
Ca          -0.0598399242     5.7317860609     5.7659527307
Ca           2.8631335460     3.0521786860     5.8827592463
Ca           2.8050599167     8.4646092753     5.8358929321
Ca           8.2648261642     3.0069461476     5.7983612874
Ca           8.1700534703     8.4716788779     5.7750873543
Ca           2.6474700000     2.5105950000     1.9196440000    0   0   0
Ca           2.6474700000     8.0080540000     1.9196440000    0   0   0
Ca           8.0417380000     2.5105950000     1.9196440000    0   0   0
Ca           8.0417380000     8.0080540000     1.9196440000    0   0   0
Ca           5.2443482734     0.1761358549     5.8018354850
Ca           5.2454890798     5.8070492027     5.7110890821
Ca          10.7007816000     0.2606208917     5.7840109356
Ca           0.0496100000     5.2593300000     1.9196450000    0   0   0
Ca           0.0496100000    10.7567880000     1.9196450000    0   0   0
Ca           5.4438780000     5.2593300000     1.9196450000    0   0   0
Ca           5.4438780000    10.7567880000     1.9196450000    0   0   0
Ti           0.0286197735     2.7398018528     3.7899032364
Ti          -0.0161415687     8.2304844682     3.7992535077
Ti           5.3871757403     2.7206212246     3.8074196229
Ti           5.3877856190     8.2409258558     3.7859615511
Ti           0.0000000000     2.7487280000     0.0000000000    0   0   0
Ti           0.1616946750     2.8699617536     7.5524509316
Ti           0.0000000000     8.2461860000     0.0000000000    0   0   0
Ti           0.0592340146     8.4045547842     7.5402986125
Ti           5.3942680000     2.7487280000     0.0000000000    0   0   0
Ti           5.6994708905     2.6228635033     7.6109192069
Ti           5.3942680000     8.2461860000     0.0000000000    0   0   0
Ti           5.5219355548     8.3621137979     7.5763246485
Ti           2.6950074585    -0.0213149101     3.7989204613
Ti           2.6592262413     5.4904486007     3.9309803621
Ti           8.0931185798    -0.0019973991     3.7965752456
Ti           8.0888970485     5.4676583513     3.7961440890
Ti           2.6971090000     0.0000000000     0.0000000000    0   0   0
Ti           2.5983297079     0.1224609771     7.5654600947
Ti           2.6971090000     5.4974580000     0.0000000000    0   0   0
Ti           2.7072836056     5.5970129978     7.9824181911
Ti           8.0913760000     0.0000000000     0.0000000000    0   0   0
Ti           8.0473024269     0.0741022514     7.5207419894
Ti           8.0913760000     5.4974580000     0.0000000000    0   0   0
Ti           7.9119159066     5.5998462022     7.5414363771
O            0.5069120470     2.6660620129     5.7612143325
O            0.4597205858     8.1442040949     5.7720301683
O            5.9314032665     2.5437488003     5.7999021426
O            5.8333926371     8.1620639267     5.7629460483
O            3.1238100000     0.1116490000     1.9196440000    0   0   0
O            3.1238100000     5.6091080000     1.9196440000    0   0   0
O            8.5180770000     0.1116490000     1.9196440000    0   0   0
O            8.5180770000     5.6091080000     1.9196440000    0   0   0
O            1.1403313952     1.1762404246     3.5046051863
O            1.1182322045     6.6720867326     3.5376137384
O            6.5408519655     1.1746208069     3.4819038893
O            6.5290871668     6.6764855864     3.5213293889
O            1.1266440000     1.1517870000     0.3184580000    0   0   0
O            1.1589286002     1.1558135381     7.9744033273
O            1.1266440000     6.6492460000     0.3184580000    0   0   0
```

O	0.8194164076	6.5409890762	8.0095879391			
O	6.5209110000	1.1517870000	0.3184580000	0	0	0
O	6.5619643153	1.0568337952	8.1043212823			
O	6.5209110000	6.6492460000	0.3184580000	0	0	0
O	6.3753379372	6.5328818331	7.9502319712			
O	4.2675512572	4.2942474482	4.2245402636			
O	4.2421914206	9.8256933328	4.1744172334			
O	9.6412604978	4.3272471448	4.1952496781			
O	9.6355799022	9.8222053861	4.1989294523			
O	1.5452030781	3.9245261761	3.4990714710			
O	1.5451364327	9.4314024075	3.5127448863			
O	6.9526986985	3.9198435821	3.5069542255			
O	6.9517831516	9.4266541081	3.5052011211			
O	1.5704400000	3.9005160000	0.3184570000	0	0	0
O	1.6127921409	3.9134458024	7.9710026228			
O	1.5704400000	9.3979740000	0.3184570000	0	0	0
O	1.5091847102	9.3570390911	7.9795866516			
O	6.9647070000	3.9005160000	0.3184570000	0	0	0
O	7.0085135472	3.8152935217	8.0008400074			
O	6.9647070000	9.3979740000	0.3184570000	0	0	0
O	6.9596638325	9.3407597892	7.9635713904			
O	3.8458096137	1.5783968358	4.2145326747			
O	3.8423025058	7.0886195597	4.1744168159			
O	9.2469459854	1.5692744259	4.1972635208			
O	9.2289379910	7.0738143454	4.1914182479			
O	3.9022853670	1.4918952381	7.3955232954			
O	3.7685591020	6.9700271368	7.4231199300			
O	9.2730447559	1.4548636007	7.3418640742			
O	9.1451405586	6.9733094475	7.4045615435			
O	4.9675650000	2.8603790000	1.9196440000	0	0	0
O	4.9675650000	8.3578380000	1.9196440000	0	0	0
O	4.4211782548	4.3258695660	7.5435114658			
O	4.1911078154	9.6963203675	7.4178370867			
O	9.6639948185	4.2364824628	7.4197591712			
O	9.5628003899	9.7366240290	7.3478936533			
O	2.2883601512	5.4043174700	5.7762972631			
O	7.5791234850	5.3703152326	5.7536270212			
O	3.8237770000	1.5969400000	-0.3184580000	0	0	0
O	3.8237770000	7.0943990000	-0.3184580000	0	0	0
O	9.2180450000	1.5969400000	-0.3184580000	0	0	0
O	9.2180450000	7.0943990000	-0.3184580000	0	0	0
O	7.5890742386	-0.0996618707	5.7609709917			
O	-0.4267020000	2.8603790000	1.9196440000	0	0	0
O	-0.4267020000	8.3578380000	1.9196440000	0	0	0
O	4.2675730000	4.3456700000	-0.3184590000	0	0	0
O	4.2675730000	9.8431280000	-0.3184590000	0	0	0
O	9.6618410000	4.3456700000	-0.3184590000	0	0	0
O	9.6618410000	9.8431280000	-0.3184590000	0	0	0
O	2.2436826158	-0.1045005330	5.7757998959			
C	2.7741991510	6.2045974764	9.9635158397			
O	3.5848039185	5.2680962975	9.9416737019			
H	2.6024632818	6.7674696846	10.9098792923			
H	5.0739947154	4.9135401731	8.0094610995			
H	0.1432467710	5.9096230451	8.3363809663			

CHOH-c1

110
CHOH_c1

Ca	-0.0939181967	5.7460978208	5.6736115788			
Ca	2.8528725357	2.9693619786	5.7633555353			
Ca	2.8297906918	8.4875529590	5.8349373819			
Ca	8.1700548807	2.9312277011	5.7162067485			
Ca	8.2052631922	8.4918359745	5.7503662374			
Ca	2.6474700000	2.5105950000	1.9196440000	0	0	0
Ca	2.6474700000	8.0080540000	1.9196440000	0	0	0
Ca	8.0417380000	2.5105950000	1.9196440000	0	0	0
Ca	8.0417380000	8.0080540000	1.9196440000	0	0	0
Ca	5.2640750486	0.2889316240	5.8241289747			
Ca	5.2478802325	5.7011941995	5.7934413677			
Ca	10.6553661959	0.1974441548	5.7655612346			
Ca	0.0496100000	5.2593300000	1.9196450000	0	0	0
Ca	0.0496100000	10.7567880000	1.9196450000	0	0	0
Ca	5.4438780000	5.2593300000	1.9196450000	0	0	0
Ca	5.4438780000	10.7567880000	1.9196450000	0	0	0
Ti	0.0032457469	2.7195335711	3.7777460316			
Ti	0.0009933641	8.2340501795	3.7805773224			
Ti	5.3909193809	2.7337711100	3.7812685158			
Ti	5.3937500522	8.2235471825	3.8382726844			
Ti	0.0000000000	2.7487280000	0.0000000000	0	0	0
Ti	0.0779141838	2.7493269435	7.5328266417			
Ti	0.0000000000	8.2461860000	0.0000000000	0	0	0
Ti	0.0969287362	8.3771958361	7.5204689078			
Ti	5.3942680000	2.7487280000	0.0000000000	0	0	0
Ti	5.5105114399	2.8877040854	7.5873498469			
Ti	5.3942680000	8.2461860000	0.0000000000	0	0	0
Ti	5.7026866649	8.3155218345	7.7688017247			
Ti	2.7029306469	-0.0222747393	3.7904395601			
Ti	2.6993275764	5.4832858769	3.7816383611			
Ti	8.0862883714	-0.0218253872	3.7835346960			
Ti	8.0894324292	5.4775350689	3.7792147235			
Ti	2.6971090000	0.0000000000	0.0000000000	0	0	0
Ti	2.5082392326	0.1937672880	7.5731705687			
Ti	2.6971090000	5.4974580000	0.0000000000	0	0	0
Ti	2.4227118537	5.4211902216	7.6377442307			
Ti	8.0913760000	0.0000000000	0.0000000000	0	0	0
Ti	7.9600731198	0.1342290070	7.5522229836			
Ti	8.0913760000	5.4974580000	0.0000000000	0	0	0
Ti	8.1918691041	5.4664951901	7.4889626461			
O	0.5037860851	2.6295390917	5.7796672307			
O	0.5147351558	8.1161373736	5.7524516398			
O	5.8349662359	2.7071780226	5.7826326934			
O	5.8612526554	8.0869071660	5.8893858365			
O	3.1238100000	0.1116490000	1.9196440000	0	0	0
O	3.1238100000	5.6091080000	1.9196440000	0	0	0
O	8.5180770000	0.1116490000	1.9196440000	0	0	0
O	8.5180770000	5.6091080000	1.9196440000	0	0	0
O	1.1533571827	1.1755588574	3.5092767473			
O	1.1377520537	6.6606060625	3.5141299451			
O	6.5436984967	1.1858776326	3.5251978324			
O	6.5259930278	6.6696940312	3.5432002945			
O	1.1266440000	1.1517870000	0.3184580000	0	0	0
O	1.0826070481	1.1834737491	8.0504931043			
O	1.1266440000	6.6492460000	0.3184580000	0	0	0

O	1.2089872275	6.7102714266	8.1086325930			
O	6.5209110000	1.1517870000	0.3184580000	0	0	0
O	6.5328548598	1.1893715054	7.9479539468			
O	6.5209110000	6.6492460000	0.3184580000	0	0	0
O	6.6910206764	6.6008395567	8.2982224725			
O	4.2337927793	4.3256934061	4.1968854486			
O	4.2461013606	9.8071370916	4.2681854991			
O	9.6253157405	4.3034951352	4.1932890950			
O	9.6320949713	9.8055447497	4.1886933714			
O	1.5503831308	3.9290459246	3.5320753783			
O	1.5586573928	9.4255630178	3.5149406767			
O	6.9480433354	3.9232726748	3.5113205122			
O	6.9433238583	9.4100351260	3.5420893014			
O	1.5704400000	3.9005160000	0.3184570000	0	0	0
O	1.4466300888	3.8919286964	8.0809368975			
O	1.5704400000	9.3979740000	0.3184570000	0	0	0
O	1.5060315180	9.4599262516	7.9186960921			
O	6.9647070000	3.9005160000	0.3184570000	0	0	0
O	6.8819048557	3.9856742200	8.0406390731			
O	6.9647070000	9.3979740000	0.3184570000	0	0	0
O	7.0390361627	9.4491337567	8.0932234603			
O	3.8621792298	1.5641965396	4.1900727195			
O	3.8326745537	7.0639499744	4.2439538269			
O	9.2572737726	1.5535259176	4.1926400673			
O	9.2440742471	7.0610768717	4.1850968420			
O	3.8582131644	1.4555178387	7.3825053766			
O	3.9099727746	6.9082914132	7.5883944060			
O	9.2886349324	1.4846117168	7.3581561150			
O	9.2886925376	6.9426045884	7.3222025803			
O	4.9675650000	2.8603790000	1.9196440000	0	0	0
O	4.9675650000	8.3578380000	1.9196440000	0	0	0
O	4.1446777132	4.2051507121	7.4411822574			
O	4.1522333062	9.6706185887	7.6766449127			
O	9.5587842790	4.1977232557	7.3107937352			
O	9.5957746155	9.6994016473	7.3104829581			
O	2.2144233305	5.3974776003	5.8304908990			
O	7.5986851464	5.3878584834	5.7769351723			
O	3.8237770000	1.5969400000	-0.3184580000	0	0	0
O	3.8237770000	7.0943990000	-0.3184580000	0	0	0
O	9.2180450000	1.5969400000	-0.3184580000	0	0	0
O	9.2180450000	7.0943990000	-0.3184580000	0	0	0
O	7.5896300666	-0.0813651981	5.7716105220			
O	-0.4267020000	2.8603790000	1.9196440000	0	0	0
O	-0.4267020000	8.3578380000	1.9196440000	0	0	0
O	4.2675730000	4.3456700000	-0.3184590000	0	0	0
O	4.2675730000	9.8431280000	-0.3184590000	0	0	0
O	9.6618410000	4.3456700000	-0.3184590000	0	0	0
O	9.6618410000	9.8431280000	-0.3184590000	0	0	0
O	2.2291688379	-0.0654499995	5.7831380624			
C	4.2113523527	7.2935661705	8.9492501358			
O	3.2114647345	8.1115253396	9.5291542261			
H	2.3209223087	7.7800685633	9.2286530928			
H	4.3841270496	6.3929026902	9.5770653664			
H	3.6322923187	9.2326076202	8.4406220729			
H	6.1273006524	5.9473362727	8.7581307625			

*CHOH-c2

```
110
CHOH_c2
Ca            -0.0764813046        5.7321210954        5.8435960706
Ca             2.8330034783        3.0031570064        5.8036718797
Ca             2.8278236411        8.4678789373        5.8710252233
Ca             8.1579436152        3.0124084466        5.7673115429
Ca             8.2732268554        8.4677947666        5.8033708943
Ca             2.6474700000        2.5105950000        1.9196440000    0   0   0
Ca             2.6474700000        8.0080540000        1.9196440000    0   0   0
Ca             8.0417380000        2.5105950000        1.9196440000    0   0   0
Ca             8.0417380000        8.0080540000        1.9196440000    0   0   0
Ca             5.2307046234        0.2810689051        5.8123641952
Ca             5.2755649011        5.7040639147        5.7201228838
Ca            10.6829010119        0.1793981132        5.7470462298
Ca             0.0496100000        5.2593300000        1.9196450000    0   0   0
Ca             0.0496100000       10.7567880000        1.9196450000    0   0   0
Ca             5.4438780000        5.2593300000        1.9196450000    0   0   0
Ca             5.4438780000       10.7567880000        1.9196450000    0   0   0
Ti             0.0101596048        2.7139493550        3.7917861653
Ti             0.0110475265        8.2171166383        3.7925632268
Ti             5.3873228172        2.7341037230        3.7940189165
Ti             5.4047036494        8.2228897172        3.7986298383
Ti             0.0000000000        2.7487280000        0.0000000000    0   0   0
Ti            -0.0685530371        2.7019239365        7.4978412923
Ti             0.0000000000        8.2461860000        0.0000000000    0   0   0
Ti             0.1224085018        8.3812835733        7.5583972109
Ti             5.3942680000        2.7487280000        0.0000000000    0   0   0
Ti             5.5231556585        2.8899403663        7.5738880645
Ti             5.3942680000        8.2461860000        0.0000000000    0   0   0
Ti             5.6142726833        8.4555928259        7.5471754346
Ti             2.7105487570       -0.0326758386        3.8054156417
Ti             2.6585298297        5.4773318422        3.9173080648
Ti             8.0757388513       -0.0193053216        3.7957221111
Ti             8.1188140501        5.4784946836        3.8052027764
Ti             2.6971090000        0.0000000000        0.0000000000    0   0   0
Ti             2.5585070711        0.1176369087        7.5803451760
Ti             2.6971090000        5.4974580000        0.0000000000    0   0   0
Ti             2.5478585221        5.5221057650        7.9028323482
Ti             8.0913760000        0.0000000000        0.0000000000    0   0   0
Ti             7.9256490905        0.0849357394        7.5467597975
Ti             8.0913760000        5.4974580000        0.0000000000    0   0   0
Ti             7.9553025439        5.6353155270        7.5702048304
O              0.4781246464        2.6076729046        5.7636486626
O              0.4870925377        8.1441102757        5.7654292286
O              5.8495075622        2.6493530901        5.7669911445
O              5.9363708664        8.1786252001        5.7662561640
O              3.1238100000        0.1116490000        1.9196440000    0   0   0
O              3.1238100000        5.6091080000        1.9196440000    0   0   0
O              8.5180770000        0.1116490000        1.9196440000    0   0   0
O              8.5180770000        5.6091080000        1.9196440000    0   0   0
O              1.1297291480        1.1707331034        3.4998647691
O              1.1385516366        6.6772489558        3.5097411152
O              6.5331374734        1.1813084286        3.4961854789
O              6.5299674583        6.6781367399        3.5098930945
O              1.1266440000        1.1517870000        0.3184580000    0   0   0
O              1.1425971696        1.1338828314        8.0424238294
O              1.1266440000        6.6492460000        0.3184580000    0   0   0
```

O	1.1766389435	6.6374252241	8.0118298832			
O	6.5209110000	1.1517870000	0.3184580000	0	0	0
O	6.5675584302	1.1655650008	7.9810454040			
O	6.5209110000	6.6492460000	0.3184580000	0	0	0
O	6.5282423171	6.6716677133	8.0425507604			
O	4.2506194789	4.3138167868	4.1660842888			
O	4.2407371234	9.8214317475	4.1961444266			
O	9.6398556483	4.3291699841	4.2068975536			
O	9.6229069059	9.8087604883	4.1852065440			
O	1.5408537910	3.9160020193	3.5151829819			
O	1.5470963186	9.4314880576	3.5093754394			
O	6.9582002769	3.9262790665	3.5328438626			
O	6.9410706477	9.4334806417	3.4757669106			
O	1.5704400000	3.9005160000	0.3184570000	0	0	0
O	1.5993170976	3.7042660377	8.1350569587			
O	1.5704400000	9.3979740000	0.3184570000	0	0	0
O	1.5980994153	9.3728352963	7.9575536093			
O	6.9647070000	3.9005160000	0.3184570000	0	0	0
O	6.9852817877	3.9159005881	7.9470097480			
O	6.9647070000	9.3979740000	0.3184570000	0	0	0
O	7.0537493787	9.4244355534	8.0446182441			
O	3.8388667151	1.5778558118	4.1909748924			
O	3.8583169931	7.0813469468	4.2416487882			
O	9.2276959269	1.5720999160	4.1965464344			
O	9.2486462487	7.0707004197	4.1854832569			
O	3.8677219300	1.4680234280	7.4371861170			
O	4.0503000076	6.9292225607	7.5356020028			
O	9.3021986356	1.4622243688	7.3309689206			
O	9.2930592753	6.9440806773	7.4034418323			
O	4.9675650000	2.8603790000	1.9196440000	0	0	0
O	4.9675650000	8.3578380000	1.9196440000	0	0	0
O	4.2294908416	4.2044605353	7.3632023875			
O	4.2662067353	9.6982961583	7.3704463163			
O	9.6688379695	4.1966536319	7.3786903677			
O	9.6422843457	9.7178650904	7.3514156294			
O	2.2522708592	5.3550831331	5.7866274431			
O	7.6125547200	5.4370332411	5.7693213144			
O	3.8237770000	1.5969400000	-0.3184580000	0	0	0
O	3.8237770000	7.0943990000	-0.3184580000	0	0	0
O	9.2180450000	1.5969400000	-0.3184580000	0	0	0
O	9.2180450000	7.0943990000	-0.3184580000	0	0	0
O	7.5777830640	-0.1109019456	5.7593326712			
O	-0.4267020000	2.8603790000	1.9196440000	0	0	0
O	-0.4267020000	8.3578380000	1.9196440000	0	0	0
O	4.2675730000	4.3456700000	-0.3184590000	0	0	0
O	4.2675730000	9.8431280000	-0.3184590000	0	0	0
O	9.6618410000	4.3456700000	-0.3184590000	0	0	0
O	9.6618410000	9.8431280000	-0.3184590000	0	0	0
O	2.2343573951	-0.0903041946	5.7705973828			
C	3.2155040313	5.3672595071	9.8929490905			
O	4.0735790666	4.3933103060	10.3198988861			
H	2.9220463963	6.0488678509	10.7134657852			
H	4.3032539981	4.4940656877	11.2655305688			
H	2.1952993068	3.0568361793	8.5661592584			
H	4.7361058922	6.4117010570	8.0451475994			

CH$_2$O-c1

109
CaTiO3

Ca	-0.1174887611	5.7531270256	5.7944789319			
Ca	2.8419467929	2.9591593547	5.9479729393			
Ca	2.8103916722	8.4861423416	5.8878947458			
Ca	8.1938880394	3.0021732483	5.7549191373			
Ca	8.2104936874	8.4707080993	5.7863532710			
Ca	2.6474700000	2.5105950000	1.9196440000	0	0	0
Ca	2.6474700000	8.0080540000	1.9196440000	0	0	0
Ca	8.0417380000	2.5105950000	1.9196440000	0	0	0
Ca	8.0417380000	8.0080540000	1.9196440000	0	0	0
Ca	5.2539291948	0.1922836502	5.8175671009			
Ca	5.2046008485	5.7515167368	5.9322330260			
Ca	10.7403618610	0.2032878148	5.8034119003			
Ca	0.0496100000	5.2593300000	1.9196450000	0	0	0
Ca	0.0496100000	10.7567880000	1.9196450000	0	0	0
Ca	5.4438780000	5.2593300000	1.9196450000	0	0	0
Ca	5.4438780000	10.7567880000	1.9196450000	0	0	0
Ti	-0.0030090770	2.6666211889	3.7797310140			
Ti	0.0042166613	8.1728299306	3.7785068784			
Ti	5.3836976347	2.6674915058	3.7869208115			
Ti	5.3790269568	8.1549230173	3.7877847099			
Ti	0.0000000000	2.7487280000	0.0000000000	0	0	0
Ti	-0.0081528663	2.7928669396	7.4993662777			
Ti	0.0000000000	8.2461860000	0.0000000000	0	0	0
Ti	0.1031587404	8.3756100263	7.5461039764			
Ti	5.3942680000	2.7487280000	0.0000000000	0	0	0
Ti	5.7009321589	2.6596196823	7.6168238771			
Ti	5.3942680000	8.2461860000	0.0000000000	0	0	0
Ti	5.6059520140	8.4188136382	7.5751285699			
Ti	2.7288243878	-0.0734249508	3.7903096624			
Ti	2.7131987822	5.4025052706	3.8669715208			
Ti	8.1109304206	-0.0762903021	3.7807091587			
Ti	8.1001412846	5.4286998313	3.7858154009			
Ti	2.6971090000	0.0000000000	0.0000000000	0	0	0
Ti	2.5954555636	0.1130490112	7.5761691852			
Ti	2.6971090000	5.4974580000	0.0000000000	0	0	0
Ti	2.2534853663	5.4491727330	7.8169019112			
Ti	8.0913760000	0.0000000000	0.0000000000	0	0	0
Ti	8.0454048433	0.0539242064	7.5253253647			
Ti	8.0913760000	5.4974580000	0.0000000000	0	0	0
Ti	7.9427797130	5.6177619760	7.5130200644			
O	0.4896240987	2.6483015967	5.7455212414			
O	0.4649406161	8.1832959250	5.7567515964			
O	5.8833097689	2.5788669671	5.7997945693			
O	5.8591343350	8.1657629225	5.7649859239			
O	3.1238100000	0.1116490000	1.9196440000	0	0	0
O	3.1238100000	5.6091080000	1.9196440000	0	0	0
O	8.5180770000	0.1116490000	1.9196440000	0	0	0
O	8.5180770000	5.6091080000	1.9196440000	0	0	0
O	1.1312272022	1.1902570076	3.4999505198			
O	1.1340633836	6.6843556833	3.5403000284			
O	6.5259768702	1.1927602857	3.4978875850			
O	6.5283268732	6.7019380306	3.4778512283			
O	1.1266440000	1.1517870000	0.3184580000	0	0	0
O	1.1569241583	1.1296859639	7.9177504732			
O	1.1266440000	6.6492460000	0.3184580000	0	0	0

O	0.9222795330	6.6228056540	7.9818272441			
O	6.5209110000	1.1517870000	0.3184580000	0	0	0
O	6.6014971024	1.0821501383	8.0878814388			
O	6.5209110000	6.6492460000	0.3184580000	0	0	0
O	6.4692007853	6.6098616283	7.9267721398			
O	4.2665487157	4.3639057212	4.2325888636			
O	4.2626761277	9.8672976692	4.1560777157			
O	9.6342523147	4.3678961132	4.1806302518			
O	9.6428653779	9.8697234755	4.1805340721			
O	1.5543129751	3.9536963277	3.5149945365			
O	1.5500801441	9.4478003407	3.5030904779			
O	6.9486865442	3.9295284613	3.5038642864			
O	6.9586860122	9.4405690661	3.5127220385			
O	1.5704400000	3.9005160000	0.3184570000	0	0	0
O	1.4888793407	3.8147114157	8.0702864635			
O	1.5704400000	9.3979740000	0.3184570000	0	0	0
O	1.5673103273	9.3626688194	7.9557646086			
O	6.9647070000	3.9005160000	0.3184570000	0	0	0
O	7.0012913824	3.8478246019	7.9833362427			
O	6.9647070000	9.3979740000	0.3184570000	0	0	0
O	7.0547242684	9.3867450071	7.9805886861			
O	3.8235614066	1.6297673084	4.1988487115			
O	3.8347774088	7.1212265690	4.2245973437			
O	9.2334511264	1.6203715906	4.1715814521			
O	9.2268471843	7.1130090994	4.1728201904			
O	3.9230857353	1.4422158547	7.4143229051			
O	3.8080866460	7.0773721776	7.5420634051			
O	9.3069087477	1.4754827938	7.3380726187			
O	9.1945546260	6.9709667537	7.3100620433			
O	4.9675650000	2.8603790000	1.9196440000	0	0	0
O	4.9675650000	8.3578380000	1.9196440000	0	0	0
O	4.3079931453	4.1839503800	7.5476001500			
O	4.2686679076	9.7130354743	7.4190917613			
O	9.6009628415	4.2069725503	7.2991153463			
O	9.6054506605	9.7482582784	7.3623308269			
O	2.2781414375	5.3717317423	5.8547523682			
O	7.5435549378	5.3856451678	5.7292086473			
O	3.8237770000	1.5969400000	-0.3184580000	0	0	0
O	3.8237770000	7.0943990000	-0.3184580000	0	0	0
O	9.2180450000	1.5969400000	-0.3184580000	0	0	0
O	9.2180450000	7.0943990000	-0.3184580000	0	0	0
O	7.6103908920	-0.0785905537	5.7604915760			
O	-0.4267020000	2.8603790000	1.9196440000	0	0	0
O	-0.4267020000	8.3578380000	1.9196440000	0	0	0
O	4.2675730000	4.3456700000	-0.3184590000	0	0	0
O	4.2675730000	9.8431280000	-0.3184590000	0	0	0
O	9.6618410000	4.3456700000	-0.3184590000	0	0	0
O	9.6618410000	9.8431280000	-0.3184590000	0	0	0
O	2.2794254132	-0.1106643483	5.7606687549			
C	3.7277559249	7.1139677517	8.9930800524			
O	3.3169681750	5.8508509245	9.4017038353			
H	3.0162474071	7.9052539559	9.2840227204			
H	4.7397022523	7.3384680990	9.4326293438			
H	4.3811136113	4.6867140893	8.4025629082			

*CH_2O-c2

110						
CH2O_c2						
Ca	-0.0780479301	5.7452851285	5.8316188550			
Ca	2.8404031243	2.9954846909	5.8071599960			
Ca	2.8291844661	8.4760508839	5.8598087197			
Ca	8.1683464582	3.0150668842	5.7575768941			
Ca	8.2590396173	8.4767062512	5.7923125691			
Ca	2.6474700000	2.5105950000	1.9196440000	0	0	0
Ca	2.6474700000	8.0080540000	1.9196440000	0	0	0
Ca	8.0417380000	2.5105950000	1.9196440000	0	0	0
Ca	8.0417380000	8.0080540000	1.9196440000	0	0	0
Ca	5.2309786719	0.2661985621	5.8068585852			
Ca	5.2634050642	5.7044660918	5.7281447482			
Ca	10.6802887063	0.1906968292	5.7495333109			
Ca	0.0496100000	5.2593300000	1.9196450000	0	0	0
Ca	0.0496100000	10.7567880000	1.9196450000	0	0	0
Ca	5.4438780000	5.2593300000	1.9196450000	0	0	0
Ca	5.4438780000	10.7567880000	1.9196450000	0	0	0
Ti	-0.0024918616	2.7278732150	3.7970573831			
Ti	0.0057213926	8.2215409120	3.7953195143			
Ti	5.3884659959	2.7286167613	3.7907356168			
Ti	5.3924612720	8.2325166640	3.8014390926			
Ti	0.0000000000	2.7487280000	0.0000000000	0	0	0
Ti	-0.0748751252	2.7174629131	7.5056231294			
Ti	0.0000000000	8.2461860000	0.0000000000	0	0	0
Ti	0.1152769753	8.3886650197	7.5511848866			
Ti	5.3942680000	2.7487280000	0.0000000000	0	0	0
Ti	5.5372668692	2.8674027437	7.5617559593			
Ti	5.3942680000	8.2461860000	0.0000000000	0	0	0
Ti	5.6094879377	8.4559578293	7.5524555663			
Ti	2.6934900545	-0.0265438657	3.8012916127			
Ti	2.6650333390	5.4747616894	3.9002644722			
Ti	8.0756712070	-0.0210423524	3.7926824470			
Ti	8.1005457668	5.4851042119	3.8003312400			
Ti	2.6971090000	0.0000000000	0.0000000000	0	0	0
Ti	2.5539238187	0.1165243706	7.5770809432			
Ti	2.6971090000	5.4974580000	0.0000000000	0	0	0
Ti	2.5153687311	5.5407085669	7.8464700476			
Ti	8.0913760000	0.0000000000	0.0000000000	0	0	0
Ti	7.9312911906	0.0849611444	7.5445009911			
Ti	8.0913760000	5.4974580000	0.0000000000	0	0	0
Ti	7.9495651126	5.6363143067	7.5593438063			
O	0.4782424139	2.6262108253	5.7731670750			
O	0.4856988183	8.1681012053	5.7553671643			
O	5.8620373842	2.6479070608	5.7574477791			
O	5.9231714648	8.1814670280	5.7678295262			
O	3.1238100000	0.1116490000	1.9196440000	0	0	0
O	3.1238100000	5.6091080000	1.9196440000	0	0	0
O	8.5180770000	0.1116490000	1.9196440000	0	0	0
O	8.5180770000	5.6091080000	1.9196440000	0	0	0
O	1.1341684787	1.1743573396	3.5083509159			
O	1.1377025621	6.6713696237	3.5257743039			
O	6.5358724502	1.1823288084	3.4912566822			
O	6.5324250340	6.6792897558	3.5108644950			
O	1.1266440000	1.1517870000	0.3184580000	0	0	0
O	1.1461925426	1.1505016691	8.0297567447			
O	1.1266440000	6.6492460000	0.3184580000	0	0	0

O	1.1120655566	6.6232132622	7.9432614842			
O	6.5209110000	1.1517870000	0.3184580000	0	0	0
O	6.5626087259	1.1571992911	7.9839864006			
O	6.5209110000	6.6492460000	0.3184580000	0	0	0
O	6.5009182440	6.6606756772	8.0294222720			
O	4.2488993549	4.3092954269	4.1733209532			
O	4.2371028240	9.8158958645	4.1998652596			
O	9.6375064632	4.3264118975	4.2082807452			
O	9.6241372370	9.8121463177	4.1848444055			
O	1.5450014943	3.9209280525	3.5278707892			
O	1.5454287738	9.4317932361	3.4954465532			
O	6.9565101494	3.9269782640	3.5187619743			
O	6.9423413551	9.4350853757	3.4778554314			
O	1.5704400000	3.9005160000	0.3184570000	0	0	0
O	1.5679019030	3.7374613233	8.1847398990			
O	1.5704400000	9.3979740000	0.3184570000	0	0	0
O	1.5840978821	9.3746493654	7.9689798679			
O	6.9647070000	3.9005160000	0.3184570000	0	0	0
O	6.9752952188	3.9146536693	7.9675705003			
O	6.9647070000	9.3979740000	0.3184570000	0	0	0
O	7.0487924130	9.4236987050	8.0376043041			
O	3.8446724826	1.5729927477	4.1935040812			
O	3.8578834421	7.0771364885	4.2358515074			
O	9.2323558251	1.5725078518	4.1948459256			
O	9.2466649531	7.0707132996	4.1881540003			
O	3.8702619306	1.4547154671	7.4213572480			
O	4.0267242773	6.9143468007	7.5218143619			
O	9.2979077581	1.4721743375	7.3328439365			
O	9.2609214924	6.9573006113	7.3818848677			
O	4.9675650000	2.8603790000	1.9196440000	0	0	0
O	4.9675650000	8.3578380000	1.9196440000	0	0	0
O	4.2256398235	4.1752573379	7.4077134701			
O	4.2555806942	9.6922992603	7.3723285096			
O	9.6537737658	4.2080044901	7.3732493338			
O	9.6316583608	9.7265759549	7.3450897770			
O	2.2816497570	5.3680336501	5.8237262581			
O	7.5960530551	5.4232621624	5.7649244786			
O	3.8237770000	1.5969400000	-0.3184580000	0	0	0
O	3.8237770000	7.0943990000	-0.3184580000	0	0	0
O	9.2180450000	1.5969400000	-0.3184580000	0	0	0
O	9.2180450000	7.0943990000	-0.3184580000	0	0	0
O	7.5793064390	-0.1035600141	5.7590618310			
O	-0.4267020000	2.8603790000	1.9196440000	0	0	0
O	-0.4267020000	8.3578380000	1.9196440000	0	0	0
O	4.2675730000	4.3456700000	-0.3184590000	0	0	0
O	4.2675730000	9.8431280000	-0.3184590000	0	0	0
O	9.6618410000	4.3456700000	-0.3184590000	0	0	0
O	9.6618410000	9.8431280000	-0.3184590000	0	0	0
O	2.2272377710	-0.0981485201	5.7687816035			
C	4.2350097368	5.2987715710	10.3507289455			
O	3.1544325215	5.5442358399	9.7093736391			
H	4.2761460201	5.5230773695	11.4232851263			
H	5.1135093382	4.8921582849	9.8284006440			
H	2.1738420035	3.0892855036	8.5991238259			
H	4.7414594586	6.4318175560	8.0300278167			

*CH_2O-c3

111						
CH2O_c3						
Ca	-0.1743197213	5.7618584625	5.7907545200			
Ca	2.7489044186	2.9696172381	5.6378592683			
Ca	2.8374151258	8.5714921625	5.8292733386			
Ca	8.1541136708	2.9850483467	5.8157251659			
Ca	8.2202916691	8.4549390939	5.7338235258			
Ca	2.6474700000	2.5105950000	1.9196440000	0	0	0
Ca	2.6474700000	8.0080540000	1.9196440000	0	0	0
Ca	8.0417380000	2.5105950000	1.9196440000	0	0	0
Ca	8.0417380000	8.0080540000	1.9196440000	0	0	0
Ca	5.2583623683	0.2672122765	5.7201151913			
Ca	5.2486257674	5.7429820746	5.8369322047			
Ca	10.7907952409	0.2377068011	5.7809708311			
Ca	0.0496100000	5.2593300000	1.9196450000	0	0	0
Ca	0.0496100000	10.7567880000	1.9196450000	0	0	0
Ca	5.4438780000	5.2593300000	1.9196450000	0	0	0
Ca	5.4438780000	10.7567880000	1.9196450000	0	0	0
Ti	-0.0338418819	2.7319352507	3.8002404991			
Ti	0.0322192994	8.2446154187	3.7891031057			
Ti	5.4079555207	2.7373306173	3.8649069823			
Ti	5.3823684229	8.2373335978	3.7986546446			
Ti	0.0000000000	2.7487280000	0.0000000000	0	0	0
Ti	0.0688914400	2.9272463054	7.5764868414			
Ti	0.0000000000	8.2461860000	0.0000000000	0	0	0
Ti	0.1740452253	8.3632788253	7.5726090026			
Ti	5.3942680000	2.7487280000	0.0000000000	0	0	0
Ti	5.5084405913	2.7743990414	7.7899506730			
Ti	5.3942680000	8.2461860000	0.0000000000	0	0	0
Ti	5.5784047470	8.3203642849	7.5847429556			
Ti	2.6694069363	-0.0051003394	3.8931482767			
Ti	2.6946757545	5.5003763668	3.7986639098			
Ti	8.1036978959	-0.0186398843	3.7919306310			
Ti	8.0771028007	5.4833291952	3.7943430427			
Ti	2.6971090000	0.0000000000	0.0000000000	0	0	0
Ti	2.9410404847	0.1162137721	7.8733971910			
Ti	2.6971090000	5.4974580000	0.0000000000	0	0	0
Ti	2.4861201989	5.7151160789	7.5620707627			
Ti	8.0913760000	0.0000000000	0.0000000000	0	0	0
Ti	8.1323563017	-0.0237826051	7.5144422648			
Ti	8.0913760000	5.4974580000	0.0000000000	0	0	0
Ti	7.9549647614	5.6290772150	7.5568585208			
O	0.4404651961	2.6434289276	5.8028272062			
O	0.4852927367	8.1641707033	5.7712585194			
O	5.8345129954	2.6315528700	5.8186386454			
O	5.8842011201	8.1408793154	5.7847356673			
O	3.1238100000	0.1116490000	1.9196440000	0	0	0
O	3.1238100000	5.6091080000	1.9196440000	0	0	0
O	8.5180770000	0.1116490000	1.9196440000	0	0	0
O	8.5180770000	5.6091080000	1.9196440000	0	0	0
O	1.1095043272	1.1859227794	3.5412465815			
O	1.1438074792	6.6793369126	3.4977766519			
O	6.5410344494	1.1716490715	3.5430538918			
O	6.5377771290	6.6755706131	3.5080542264			
O	1.1266440000	1.1517870000	0.3184580000	0	0	0
O	1.0253358128	1.1305818730	8.1217059730			
O	1.1266440000	6.6492460000	0.3184580000	0	0	0

O	1.0435498555	6.6950092445	8.0421111828			
O	6.5209110000	1.1517870000	0.3184580000	0	0	0
O	6.4500453364	0.9932102510	8.1413016595			
O	6.5209110000	6.6492460000	0.3184580000	0	0	0
O	6.4704830203	6.6321906803	7.9902391314			
O	4.2399115099	4.3401347197	4.2360962385			
O	4.2656126609	9.8062338044	4.1718915301			
O	9.6258370800	4.3232299222	4.2057509649			
O	9.6530584313	9.8326643662	4.2047038980			
O	1.5538574756	3.9269897925	3.5375853248			
O	1.5391231773	9.4120002732	3.4726640550			
O	6.9538436848	3.9273115607	3.5081485068			
O	6.9647176510	9.4179842048	3.5216730894			
O	1.5704400000	3.9005160000	0.3184570000	0	0	0
O	1.5253806011	3.9114208299	8.0187046386			
O	1.5704400000	9.3979740000	0.3184570000	0	0	0
O	1.4967606428	9.4661920732	7.9905347244			
O	6.9647070000	3.9005160000	0.3184570000	0	0	0
O	6.8757229549	3.8837262121	8.0397264432			
O	6.9647070000	9.3979740000	0.3184570000	0	0	0
O	6.9373436103	9.3987730591	8.0450425722			
O	3.8432066309	1.5868881405	4.1126923751			
O	3.8434659152	7.0789597319	4.2115744620			
O	9.2229246509	1.5880192063	4.2015701476			
O	9.2444887397	7.0593190074	4.1881348184			
O	3.7819573432	1.5256631484	7.0674674149			
O	3.8215597617	6.9685098778	7.3750674814			
O	9.1995639099	1.4729295241	7.4518214169			
O	9.2031463006	6.9703049526	7.3485351259			
O	4.9675650000	2.8603790000	1.9196440000	0	0	0
O	4.9675650000	8.3578380000	1.9196440000	0	0	0
O	4.0455714136	4.1970360200	7.6572999346			
O	4.2334395165	9.7376932747	7.3957049468			
O	9.5540227421	4.2272271967	7.3971463374			
O	9.5723236770	9.7451390206	7.3888075059			
O	2.1752670232	5.4286942460	5.7821459846			
O	7.5736848472	5.3905153920	5.7727994109			
O	3.8237770000	1.5969400000	-0.3184580000	0	0	0
O	3.8237770000	7.0943990000	-0.3184580000	0	0	0
O	9.2180450000	1.5969400000	-0.3184580000	0	0	0
O	9.2180450000	7.0943990000	-0.3184580000	0	0	0
O	7.6207467739	-0.1100087334	5.7802755313			
O	-0.4267020000	2.8603790000	1.9196440000	0	0	0
O	-0.4267020000	8.3578380000	1.9196440000	0	0	0
O	4.2675730000	4.3456700000	-0.3184590000	0	0	0
O	4.2675730000	9.8431280000	-0.3184590000	0	0	0
O	9.6618410000	4.3456700000	-0.3184590000	0	0	0
O	9.6618410000	9.8431280000	-0.3184590000	0	0	0
O	2.2620582214	-0.1250441105	5.7090790249			
C	4.3252556855	2.0061849370	9.6379192683			
O	3.7452885269	0.7220664866	9.4102577631			
H	3.5197768256	2.7153410779	9.9073873160			
H	5.0165705759	1.9390856695	10.4983213564			
H	0.4490504719	0.4585763995	8.5374573142			
H	3.3431999804	3.6231389865	8.0729594914			
H	5.8351094302	0.3971423758	8.6226784821			

CH_3O-c1

110
CH3O_c1

Ca	-0.0605331384	5.7048181289	5.9106376005			
Ca	2.8395658093	3.0159856068	6.0067736849			
Ca	2.7949564872	8.4384513669	5.8722141094			
Ca	8.2586650750	2.9869015348	5.8011457575			
Ca	8.1680460773	8.4493535081	5.8599092487			
Ca	2.6474700000	2.5105950000	1.9196440000	0	0	0
Ca	2.6474700000	8.0080540000	1.9196440000	0	0	0
Ca	8.0417380000	2.5105950000	1.9196440000	0	0	0
Ca	8.0417380000	8.0080540000	1.9196440000	0	0	0
Ca	5.2722715619	0.1433369049	5.8644065416			
Ca	5.1999404018	5.7253427441	5.9438868630			
Ca	10.7113829005	0.2180652298	5.8585369962			
Ca	0.0496100000	5.2593300000	1.9196450000	0	0	0
Ca	0.0496100000	10.7567880000	1.9196450000	0	0	0
Ca	5.4438780000	5.2593300000	1.9196450000	0	0	0
Ca	5.4438780000	10.7567880000	1.9196450000	0	0	0
Ti	0.0208661051	2.6557206786	3.7944614459			
Ti	-0.0270267122	8.1387003956	3.8069418003			
Ti	5.3657405080	2.6418125255	3.8103363761			
Ti	5.3793444045	8.1339783382	3.7992077304			
Ti	0.0000000000	2.7487280000	0.0000000000	0	0	0
Ti	0.1166604338	2.8742433565	7.5293737657			
Ti	0.0000000000	8.2461860000	0.0000000000	0	0	0
Ti	0.0979350102	8.3975059846	7.5661488828			
Ti	5.3942680000	2.7487280000	0.0000000000	0	0	0
Ti	5.7337712863	2.6557198677	7.6053877706			
Ti	5.3942680000	8.2461860000	0.0000000000	0	0	0
Ti	5.4910116868	8.3482632410	7.5651155412			
Ti	2.7126837922	-0.1039522835	3.8040507265			
Ti	2.6980853265	5.3944737977	3.9186616211			
Ti	8.1130059828	-0.0897062635	3.8001686941			
Ti	8.0980914891	5.3971842904	3.8072842408			
Ti	2.6971090000	0.0000000000	0.0000000000	0	0	0
Ti	2.6018904591	0.1250679991	7.5544547307			
Ti	2.6971090000	5.4974580000	0.0000000000	0	0	0
Ti	2.4854921266	5.7460266263	7.8982667739			
Ti	8.0913760000	0.0000000000	0.0000000000	0	0	0
Ti	8.0507157990	0.0698652816	7.5351537385			
Ti	8.0913760000	5.4974580000	0.0000000000	0	0	0
Ti	7.9493455435	5.6069725262	7.5305341746			
O	0.4808823804	2.6429045655	5.7257291044			
O	0.4475126271	8.1857989456	5.7614061275			
O	5.9283321076	2.5730471326	5.7883149557			
O	5.8333668646	8.1509517445	5.7431132219			
O	3.1238100000	0.1116490000	1.9196440000	0	0	0
O	3.1238100000	5.6091080000	1.9196440000	0	0	0
O	8.5180770000	0.1116490000	1.9196440000	0	0	0
O	8.5180770000	5.6091080000	1.9196440000	0	0	0
O	1.1423254699	1.2003803418	3.4723133953			
O	1.1188720407	6.7045798248	3.5168857436			
O	6.5294233407	1.2068340235	3.4782068539			
O	6.5360956975	6.7113292084	3.4681256552			
O	1.1266440000	1.1517870000	0.3184580000	0	0	0
O	1.1865649728	1.2080174974	7.9356312044			
O	1.1266440000	6.6492460000	0.3184580000	0	0	0

O	1.0114220045	6.7475496683	8.0667665204			
O	6.5209110000	1.1517870000	0.3184580000	0	0	0
O	6.5888001729	1.0744618560	8.1149002987			
O	6.5209110000	6.6492460000	0.3184580000	0	0	0
O	6.4834323996	6.6066869479	7.9463139254			
O	4.2755965483	4.3587109294	4.2289838784			
O	4.2627814839	9.8860375036	4.1598258886			
O	9.6456610848	4.3979415224	4.2009216467			
O	9.6545313208	9.8944109975	4.1802087727			
O	1.5434241702	3.9553857466	3.4834148523			
O	1.5609319320	9.4548059684	3.4935003750			
O	6.9612573180	3.9478326494	3.4890157014			
O	6.9618897697	9.4558529462	3.4969445971			
O	1.5704400000	3.9005160000	0.3184570000	0	0	0
O	1.5265087585	3.9706421883	7.8488782227			
O	1.5704400000	9.3979740000	0.3184570000	0	0	0
O	1.4994829488	9.4525132898	7.9529866149			
O	6.9647070000	3.9005160000	0.3184570000	0	0	0
O	7.0307627472	3.8358155377	7.9915733931			
O	6.9647070000	9.3979740000	0.3184570000	0	0	0
O	6.9224101244	9.3738691749	7.9211808246			
O	3.8334897681	1.6445225881	4.2036623949			
O	3.8304861437	7.1390419490	4.1545861416			
O	9.2375260955	1.6350641028	4.1545198712			
O	9.2099016915	7.1453724237	4.1731294941			
O	3.9426440292	1.4183227926	7.3955939176			
O	3.8154563039	6.9817045069	7.3652139010			
O	9.2891946447	1.4480433372	7.3424204937			
O	9.2253199824	6.9472180331	7.3434992648			
O	4.9675650000	2.8603790000	1.9196440000	0	0	0
O	4.9675650000	8.3578380000	1.9196440000	0	0	0
O	4.3399942835	4.1329845478	7.5369894949			
O	4.1736600000	9.6830166958	7.3956773015			
O	9.5981438882	4.1985954424	7.3396309447			
O	9.5416081376	9.7273455250	7.3996600462			
O	2.2896352446	5.4376242572	5.7640512750			
O	7.5553054457	5.3933820091	5.7357018561			
O	3.8237770000	1.5969400000	-0.3184580000	0	0	0
O	3.8237770000	7.0943990000	-0.3184580000	0	0	0
O	9.2180450000	1.5969400000	-0.3184580000	0	0	0
O	9.2180450000	7.0943990000	-0.3184580000	0	0	0
O	7.6139785384	-0.0775705624	5.7518210419			
O	-0.4267020000	2.8603790000	1.9196440000	0	0	0
O	-0.4267020000	8.3578380000	1.9196440000	0	0	0
O	4.2675730000	4.3456700000	-0.3184590000	0	0	0
O	4.2675730000	9.8431280000	-0.3184590000	0	0	0
O	9.6618410000	4.3456700000	-0.3184590000	0	0	0
O	9.6618410000	9.8431280000	-0.3184590000	0	0	0
O	2.2473178198	-0.0859351042	5.7438987664			
C	4.0685502294	6.2758005564	10.4026847558			
O	3.2587143851	5.4494566371	9.5849600154			
H	3.8328576682	7.3445703543	10.2515305233			
H	3.8907296747	6.0305074446	11.4618133247			
H	5.1357726970	6.1182974825	10.1730870808			
H	4.2202777072	4.4652529698	8.4729040402			

*CH_2OH-c1

110						
CH2OH_c1						
Ca	-0.1917533750	5.7169880685	5.8583758472			
Ca	2.7878654058	2.9451632858	5.7171555968			
Ca	2.8258350507	8.5249914697	5.8832115339			
Ca	8.1222022030	2.9308674217	5.9301657311			
Ca	8.1848161807	8.4263733698	5.8395736105			
Ca	2.6474700000	2.5105950000	1.9196440000	0	0	0
Ca	2.6474700000	8.0080540000	1.9196440000	0	0	0
Ca	8.0417380000	2.5105950000	1.9196440000	0	0	0
Ca	8.0417380000	8.0080540000	1.9196440000	0	0	0
Ca	5.3069105110	0.1951554834	5.8335596066			
Ca	5.2678869244	5.5552274285	6.0652837559			
Ca	10.7689668201	0.2127984689	5.8866621926			
Ca	0.0496100000	5.2593300000	1.9196450000	0	0	0
Ca	0.0496100000	10.7567880000	1.9196450000	0	0	0
Ca	5.4438780000	5.2593300000	1.9196450000	0	0	0
Ca	5.4438780000	10.7567880000	1.9196450000	0	0	0
Ti	-0.0470797377	2.6518474990	3.8108088134			
Ti	0.0044091436	8.1605969378	3.8001110732			
Ti	5.4097370918	2.6510516938	3.8824590660			
Ti	5.3616615135	8.1160784721	3.8183903144			
Ti	0.0000000000	2.7487280000	0.0000000000	0	0	0
Ti	0.1004230058	2.8779080995	7.5651796292			
Ti	0.0000000000	8.2461860000	0.0000000000	0	0	0
Ti	0.1465294557	8.3754921456	7.5605142604			
Ti	5.3942680000	2.7487280000	0.0000000000	0	0	0
Ti	5.5628064406	2.6154715408	7.8395297345			
Ti	5.3942680000	8.2461860000	0.0000000000	0	0	0
Ti	5.5256874808	8.3572432574	7.5645297882			
Ti	2.7195512693	-0.0897535353	3.8479985615			
Ti	2.7045373541	5.4166056835	3.8061857171			
Ti	8.1183329196	-0.1104600779	3.7945761780			
Ti	8.0883238129	5.3993268450	3.8034922566			
Ti	2.6971090000	0.0000000000	0.0000000000	0	0	0
Ti	2.6754069379	0.2022084737	7.6632377166			
Ti	2.6971090000	5.4974580000	0.0000000000	0	0	0
Ti	2.4553114070	5.6912805918	7.5647607100			
Ti	8.0913760000	0.0000000000	0.0000000000	0	0	0
Ti	8.0880884222	0.0226035285	7.5169908649			
Ti	8.0913760000	5.4974580000	0.0000000000	0	0	0
Ti	7.9544596413	5.6110240664	7.5713255516			
O	0.4563169820	2.7093539135	5.7592014356			
O	0.4732590880	8.1595406125	5.7537489894			
O	5.7896074356	2.6991231431	5.7497691273			
O	5.8445660327	8.1196485395	5.7498575931			
O	3.1238100000	0.1116490000	1.9196440000	0	0	0
O	3.1238100000	5.6091080000	1.9196440000	0	0	0
O	8.5180770000	0.1116490000	1.9196440000	0	0	0
O	8.5180770000	5.6091080000	1.9196440000	0	0	0
O	1.1066779782	1.2095080247	3.5255356408			
O	1.1273510394	6.7067937493	3.4751354311			
O	6.5325822849	1.1913695894	3.5194744333			
O	6.5380660011	6.7079617264	3.4668235330			
O	1.1266440000	1.1517870000	0.3184580000	0	0	0
O	1.1805888569	1.1969668241	7.9214812290			
O	1.1266440000	6.6492460000	0.3184580000	0	0	0

O	1.0351801025	6.7000435910	8.0352561436			
O	6.5209110000	1.1517870000	0.3184580000	0	0	0
O	6.5827682409	1.0910470625	7.9286784345			
O	6.5209110000	6.6492460000	0.3184580000	0	0	0
O	6.4891847526	6.6265380723	7.9704655876			
O	4.2305920836	4.4119508675	4.2306609500			
O	4.2716328176	9.8722926293	4.1669912807			
O	9.6408262778	4.3967973972	4.1667124354			
O	9.6755425679	9.8999259055	4.1787109890			
O	1.5662673672	3.9576505491	3.5038598250			
O	1.5519477677	9.4491673375	3.4817715797			
O	6.9468877876	3.9583903863	3.4795459375			
O	6.9739503036	9.4436624606	3.5093374817			
O	1.5704400000	3.9005160000	0.3184570000	0	0	0
O	1.4852628196	3.9477957918	8.0654367585			
O	1.5704400000	9.3979740000	0.3184570000	0	0	0
O	1.5234204561	9.4474674155	7.9369221868			
O	6.9647070000	3.9005160000	0.3184570000	0	0	0
O	6.8261545497	3.9026907339	7.9854621590			
O	6.9647070000	9.3979740000	0.3184570000	0	0	0
O	6.9548192749	9.3776587197	7.9451680176			
O	3.8139930483	1.6330431697	4.1088183551			
O	3.7990423943	7.1493817710	4.1627158151			
O	9.2027216515	1.6600140392	4.1886833505			
O	9.2286358476	7.1342215920	4.1824063243			
O	3.8871893494	1.4788120397	7.1162712057			
O	3.8202194389	6.9233039677	7.3926367339			
O	9.3031440042	1.4326648178	7.3998074481			
O	9.2216319918	6.9441004943	7.3671253585			
O	4.9675650000	2.8603790000	1.9196440000	0	0	0
O	4.9675650000	8.3578380000	1.9196440000	0	0	0
O	3.9945421211	4.1547003434	7.5994037855			
O	4.2198267366	9.6917711384	7.3890027108			
O	9.5362224304	4.1821181350	7.4054888516			
O	9.5692989829	9.7045599709	7.3781022420			
O	2.1478808252	5.4300678677	5.7682998774			
O	7.5941601767	5.3932619059	5.7584489292			
O	3.8237770000	1.5969400000	-0.3184580000	0	0	0
O	3.8237770000	7.0943990000	-0.3184580000	0	0	0
O	9.2180450000	1.5969400000	-0.3184580000	0	0	0
O	9.2180450000	7.0943990000	-0.3184580000	0	0	0
O	7.6560615897	-0.1074224095	5.7456652752			
O	-0.4267020000	2.8603790000	1.9196440000	0	0	0
O	-0.4267020000	8.3578380000	1.9196440000	0	0	0
O	4.2675730000	4.3456700000	-0.3184590000	0	0	0
O	4.2675730000	9.8431280000	-0.3184590000	0	0	0
O	9.6618410000	4.3456700000	-0.3184590000	0	0	0
O	9.6618410000	9.8431280000	-0.3184590000	0	0	0
O	2.2689606818	-0.1024538280	5.7414435235			
C	4.4523747307	2.0669371440	9.7191926817			
O	3.6237751466	0.8313446045	9.5669245386			
H	3.7852692693	2.8625374045	10.0952492169			
H	5.2016567051	1.8490944362	10.4990550681			
H	3.1260205572	0.6905939192	10.3938798567			
H	3.3504643371	3.6084062179	8.1149374014			

CH$_2$OH-c2

110						
CH2OH_c2						
Ca	-0.0724136344	5.7495318061	5.7414064781			
Ca	2.8426339742	2.9607293272	5.7917344723			
Ca	2.8279792589	8.4997291170	5.8385526240			
Ca	8.1804328565	2.9424231101	5.7175833657			
Ca	8.2432981201	8.4753962835	5.7881052989			
Ca	2.6474700000	2.5105950000	1.9196440000	0	0	0
Ca	2.6474700000	8.0080540000	1.9196440000	0	0	0
Ca	8.0417380000	2.5105950000	1.9196440000	0	0	0
Ca	8.0417380000	8.0080540000	1.9196440000	0	0	0
Ca	5.2653763807	0.2956745183	5.7882294887			
Ca	5.2495481072	5.6590538555	5.7733496951			
Ca	10.6748209348	0.1744734661	5.7566335905			
Ca	0.0496100000	5.2593300000	1.9196450000	0	0	0
Ca	0.0496100000	10.7567880000	1.9196450000	0	0	0
Ca	5.4438780000	5.2593300000	1.9196450000	0	0	0
Ca	5.4438780000	10.7567880000	1.9196450000	0	0	0
Ti	0.0021085770	2.7154171620	3.7796067209			
Ti	0.0041721623	8.2283420786	3.7840986472			
Ti	5.3923737728	2.7338766327	3.7795564079			
Ti	5.3904643220	8.2146187059	3.7902948492			
Ti	0.0000000000	2.7487280000	0.0000000000	0	0	0
Ti	0.0353506493	2.6737475372	7.5309777855			
Ti	0.0000000000	8.2461860000	0.0000000000	0	0	0
Ti	0.1475381240	8.3762896904	7.5532720362			
Ti	5.3942680000	2.7487280000	0.0000000000	0	0	0
Ti	5.5134190209	2.8714855617	7.5946150409			
Ti	5.3942680000	8.2461860000	0.0000000000	0	0	0
Ti	5.5848259604	8.4618413864	7.5851217827			
Ti	2.7094421781	-0.0281308003	3.7872798463			
Ti	2.6951499974	5.4742656547	3.8104387304			
Ti	8.0879452397	-0.0291570849	3.7848199239			
Ti	8.1021588509	5.4748877877	3.7889376070			
Ti	2.6971090000	0.0000000000	0.0000000000	0	0	0
Ti	2.5805513625	0.1196448140	7.5618277153			
Ti	2.6971090000	5.4974580000	0.0000000000	0	0	0
Ti	2.3443048524	5.3501908818	7.7209867141			
Ti	8.0913760000	0.0000000000	0.0000000000	0	0	0
Ti	7.9509897972	0.0970171174	7.5553156399			
Ti	8.0913760000	5.4974580000	0.0000000000	0	0	0
Ti	8.1556322732	5.4778894700	7.5152883678			
O	0.4815470217	2.6124568107	5.7841301604			
O	0.5021823542	8.1542666371	5.7660312210			
O	5.8463673551	2.7159608498	5.7923445896			
O	5.8833662293	8.0554304526	5.8439912430			
O	3.1238100000	0.1116490000	1.9196440000	0	0	0
O	3.1238100000	5.6091080000	1.9196440000	0	0	0
O	8.5180770000	0.1116490000	1.9196440000	0	0	0
O	8.5180770000	5.6091080000	1.9196440000	0	0	0
O	1.1498723151	1.1704858358	3.5110576167			
O	1.1428157400	6.6580010806	3.5340243933			
O	6.5384066311	1.1789046232	3.5310415639			
O	6.5321700927	6.6658058503	3.5246483966			
O	1.1266440000	1.1517870000	0.3184580000	0	0	0
O	1.1123717124	1.1234903537	7.9942307939			
O	1.1266440000	6.6492460000	0.3184580000	0	0	0

O	1.0686305114	6.6099270768	7.9756118956			
O	6.5209110000	1.1517870000	0.3184580000	0	0	0
O	6.5542173452	1.1718204514	7.9694130616			
O	6.5209110000	6.6492460000	0.3184580000	0	0	0
O	6.5483229321	6.6119423430	8.2547988254			
O	4.2470709192	4.3176550850	4.1846829991			
O	4.2501707190	9.8035004058	4.2218847743			
O	9.6341582867	4.3060797740	4.2066071596			
O	9.6292883869	9.7972716722	4.1873162304			
O	1.5546308287	3.9218920389	3.5395845806			
O	1.5565351493	9.4211369428	3.5134255979			
O	6.9545923670	3.9171938048	3.5128917472			
O	6.9400224862	9.4082312245	3.5326354832			
O	1.5704400000	3.9005160000	0.3184570000	0	0	0
O	1.4388845172	3.8300753781	8.0947987696			
O	1.5704400000	9.3979740000	0.3184570000	0	0	0
O	1.6261018696	9.3618088649	7.9829242783			
O	6.9647070000	3.9005160000	0.3184570000	0	0	0
O	6.8695076911	3.9732059446	8.0906057947			
O	6.9647070000	9.3979740000	0.3184570000	0	0	0
O	7.0473998275	9.3991173972	8.0386616909			
O	3.8549704351	1.5677364617	4.1983487595			
O	3.8489019266	7.0588837489	4.2546287512			
O	9.2509562902	1.5513483076	4.1898586751			
O	9.2499252618	7.0520057307	4.2034307714			
O	3.8673324141	1.4691901489	7.4045549714			
O	3.9052384013	7.1019545121	7.7898928416			
O	9.2904578432	1.4683248478	7.3578968545			
O	9.2307635546	6.9503315348	7.3476473397			
O	4.9675650000	2.8603790000	1.9196440000	0	0	0
O	4.9675650000	8.3578380000	1.9196440000	0	0	0
O	4.1807713997	4.2342077207	7.4703125125			
O	4.2913146205	9.7482815167	7.3732563501			
O	9.5448017525	4.1949342655	7.3416172420			
O	9.6443879238	9.7069828450	7.3645922253			
O	2.2692384910	5.3628369149	5.8516565455			
O	7.5847861359	5.3657277939	5.7874967961			
O	3.8237770000	1.5969400000	-0.3184580000	0	0	0
O	3.8237770000	7.0943990000	-0.3184580000	0	0	0
O	9.2180450000	1.5969400000	-0.3184580000	0	0	0
O	9.2180450000	7.0943990000	-0.3184580000	0	0	0
O	7.6049880369	-0.1024247331	5.7739537867			
O	-0.4267020000	2.8603790000	1.9196440000	0	0	0
O	-0.4267020000	8.3578380000	1.9196440000	0	0	0
O	4.2675730000	4.3456700000	-0.3184590000	0	0	0
O	4.2675730000	9.8431280000	-0.3184590000	0	0	0
O	9.6618410000	4.3456700000	-0.3184590000	0	0	0
O	9.6618410000	9.8431280000	-0.3184590000	0	0	0
O	2.2335322526	-0.0967977138	5.7781117959			
C	3.6704673176	7.2029270079	9.1679404503			
O	3.2955473995	5.8841689877	9.6701265917			
H	2.8122016014	7.8526186225	9.3817820034			
H	4.5695841188	7.5349341039	9.7316175370			
H	4.0855389568	5.3008529930	9.6382676940			
H	5.9681545536	5.8683289815	8.5168876312			

CH$_2$OH-c3

110
CH2OH_c3

Ca	-0.0962270598	5.7104812561	5.8520859810			
Ca	2.8167861176	2.9393209076	5.8783488794			
Ca	2.8054716449	8.5316980518	5.7425945415			
Ca	8.1748921774	2.9644745272	5.8364810066			
Ca	8.2523139902	8.4723361661	5.8593048701			
Ca	2.6474700000	2.5105950000	1.9196440000	0	0	0
Ca	2.6474700000	8.0080540000	1.9196440000	0	0	0
Ca	8.0417380000	2.5105950000	1.9196440000	0	0	0
Ca	8.0417380000	8.0080540000	1.9196440000	0	0	0
Ca	5.2840848972	0.2479060895	5.8512668063			
Ca	5.2244631726	5.7354405476	5.8875705553			
Ca	10.6958509236	0.1945190347	5.8382500504			
Ca	0.0496100000	5.2593300000	1.9196450000	0	0	0
Ca	0.0496100000	10.7567880000	1.9196450000	0	0	0
Ca	5.4438780000	5.2593300000	1.9196450000	0	0	0
Ca	5.4438780000	10.7567880000	1.9196450000	0	0	0
Ti	-0.0163665647	2.6597886070	3.7859591909			
Ti	-0.0147639821	8.1596135884	3.7880335804			
Ti	5.3792953090	2.6805381074	3.7891616096			
Ti	5.4017309379	8.1616079155	3.7868160492			
Ti	0.0000000000	2.7487280000	0.0000000000	0	0	0
Ti	0.0238574786	2.7781093542	7.5237677499			
Ti	0.0000000000	8.2461860000	0.0000000000	0	0	0
Ti	0.1534908787	8.3358776088	7.5555613151			
Ti	5.3942680000	2.7487280000	0.0000000000	0	0	0
Ti	5.5135657746	2.8712575998	7.5736229993			
Ti	5.3942680000	8.2461860000	0.0000000000	0	0	0
Ti	5.6005742248	8.4108854401	7.5663396227			
Ti	2.7260411494	-0.0642033798	3.7869080766			
Ti	2.7098338644	5.4064973373	3.8224136358			
Ti	8.1067894287	-0.0783564686	3.7913301272			
Ti	8.1101527369	5.4122520530	3.7904329671			
Ti	2.6971090000	0.0000000000	0.0000000000	0	0	0
Ti	2.5961687467	0.0996221309	7.5680330185			
Ti	2.6971090000	5.4974580000	0.0000000000	0	0	0
Ti	2.4460116719	5.3977324047	7.7053206407			
Ti	8.0913760000	0.0000000000	0.0000000000	0	0	0
Ti	7.9701962248	0.1079025413	7.5655070631			
Ti	8.0913760000	5.4974580000	0.0000000000	0	0	0
Ti	7.9480964153	5.6081245614	7.5313677918			
O	0.4734011652	2.6261506340	5.7552596114			
O	0.4877499323	8.1248592728	5.7583339306			
O	5.8444746077	2.6733966186	5.7704115790			
O	5.8857994205	8.1579921980	5.7674931127			
O	3.1238100000	0.1116490000	1.9196440000	0	0	0
O	3.1238100000	5.6091080000	1.9196440000	0	0	0
O	8.5180770000	0.1116490000	1.9196440000	0	0	0
O	8.5180770000	5.6091080000	1.9196440000	0	0	0
O	1.1359897822	1.1957323119	3.4901101606			
O	1.1467792029	6.6892767544	3.5156893553			
O	6.5332220873	1.2008968971	3.5146714831			
O	6.5415415836	6.7031967957	3.4820414219			
O	1.1266440000	1.1517870000	0.3184580000	0	0	0
O	1.1542797518	1.0952960356	7.9661122431			
O	1.1266440000	6.6492460000	0.3184580000	0	0	0

O	1.1041140666	6.5766442478	8.0209619869			
O	6.5209110000	1.1517870000	0.3184580000	0	0	0
O	6.5777211432	1.1711962095	7.9691274826			
O	6.5209110000	6.6492460000	0.3184580000	0	0	0
O	6.4900990180	6.6473867379	7.9369342239			
O	4.2764829734	4.3811087919	4.1799729078			
O	4.2626534243	9.8760094643	4.1792584955			
O	9.6512282032	4.3728938844	4.1874772091			
O	9.6549987077	9.8619111508	4.1826775590			
O	1.5643226604	3.9427968883	3.5160660060			
O	1.5733680899	9.4387940552	3.5215119706			
O	6.9635605334	3.9385471538	3.4940855826			
O	6.9533976838	9.4472482731	3.5013889122			
O	1.5704400000	3.9005160000	0.3184570000	0	0	0
O	1.5222655306	3.8187413834	8.0148036364			
O	1.5704400000	9.3979740000	0.3184570000	0	0	0
O	1.6704676073	9.2899598286	7.9593608643			
O	6.9647070000	3.9005160000	0.3184570000	0	0	0
O	6.9451613874	3.9139624283	7.9889822963			
O	6.9647070000	9.3979740000	0.3184570000	0	0	0
O	7.0395629593	9.4107718732	8.0019910139			
O	3.8294790964	1.6306010062	4.1794592881			
O	3.8548337546	7.1184233984	4.2086632897			
O	9.2280761850	1.6230222058	4.1831975575			
O	9.2339254538	7.1158897168	4.1837374236			
O	3.9019161670	1.4571023727	7.4088149706			
O	3.7696377480	7.1248964550	7.6039822202			
O	9.3195295932	1.4525928205	7.3843567730			
O	9.2690072465	6.9265368651	7.3724442954			
O	4.9675650000	2.8603790000	1.9196440000	0	0	0
O	4.9675650000	8.3578380000	1.9196440000	0	0	0
O	4.2114325352	4.2411780290	7.4177568369			
O	4.2772797458	9.7206823781	7.3864988503			
O	9.5971541317	4.1773190021	7.3470025234			
O	9.6756817760	9.6863204941	7.3928170079			
O	2.2711304895	5.3696851731	5.8207701880			
O	7.5766854705	5.3992567029	5.7423913598			
O	3.8237770000	1.5969400000	-0.3184580000	0	0	0
O	3.8237770000	7.0943990000	-0.3184580000	0	0	0
O	9.2180450000	1.5969400000	-0.3184580000	0	0	0
O	9.2180450000	7.0943990000	-0.3184580000	0	0	0
O	7.6392191706	-0.0889053112	5.7661830281			
O	-0.4267020000	2.8603790000	1.9196440000	0	0	0
O	-0.4267020000	8.3578380000	1.9196440000	0	0	0
O	4.2675730000	4.3456700000	-0.3184590000	0	0	0
O	4.2675730000	9.8431280000	-0.3184590000	0	0	0
O	9.6618410000	4.3456700000	-0.3184590000	0	0	0
O	9.6618410000	9.8431280000	-0.3184590000	0	0	0
O	2.2484447449	-0.0843850169	5.7562194021			
C	4.3504981161	5.8142865658	10.3686343584			
O	3.0874426464	5.5116956724	9.8895253299			
H	4.4653375200	5.9317603983	11.4425069376			
H	5.1496041279	5.6215337733	9.6591411789			
H	2.3828633477	5.7956741031	10.5010327854			
H	3.1552496389	7.7609895943	8.0484471760			

CH$_3$OH

110
CH3OH

Ca	-0.0517574814	5.6580358812	5.9539657645			
Ca	2.7975856251	2.9819532612	5.9206759434			
Ca	2.8104914397	8.4360916246	5.9010427676			
Ca	8.2080078310	2.9423448287	5.8924931749			
Ca	8.2197955365	8.4337723597	5.9061991481			
Ca	2.6474700000	2.5105950000	1.9196440000	0	0	0
Ca	2.6474700000	8.0080540000	1.9196440000	0	0	0
Ca	8.0417380000	2.5105950000	1.9196440000	0	0	0
Ca	8.0417380000	8.0080540000	1.9196440000	0	0	0
Ca	5.2756536335	0.2142821475	5.8878635563			
Ca	5.2753078972	5.6999290630	5.8931245540			
Ca	10.6683708833	0.1920552903	5.9041459457			
Ca	0.0496100000	5.2593300000	1.9196450000	0	0	0
Ca	0.0496100000	10.7567880000	1.9196450000	0	0	0
Ca	5.4438780000	5.2593300000	1.9196450000	0	0	0
Ca	5.4438780000	10.7567880000	1.9196450000	0	0	0
Ti	-0.0117884326	2.6364952018	3.8087530084			
Ti	-0.0186781686	8.1292026517	3.8124339163			
Ti	5.3708574070	2.6474082293	3.8118133815			
Ti	5.3853166577	8.1399388574	3.8049583616			
Ti	0.0000000000	2.7487280000	0.0000000000	0	0	0
Ti	0.1103361985	2.8345682720	7.5594397825			
Ti	0.0000000000	8.2461860000	0.0000000000	0	0	0
Ti	0.1235372760	8.3520049968	7.5638439191			
Ti	5.3942680000	2.7487280000	0.0000000000	0	0	0
Ti	5.5200758313	2.8555816357	7.5641192359			
Ti	5.3942680000	8.2461860000	0.0000000000	0	0	0
Ti	5.5259307746	8.3636021729	7.5485024218			
Ti	2.7116083669	-0.1097417019	3.8117540302			
Ti	2.7095404740	5.3951511599	3.8483016572			
Ti	8.1042693038	-0.1070307242	3.8115705498			
Ti	8.1176282933	5.3862716258	3.8147910598			
Ti	2.6971090000	0.0000000000	0.0000000000	0	0	0
Ti	2.5785665415	0.1088153916	7.5584085770			
Ti	2.6971090000	5.4974580000	0.0000000000	0	0	0
Ti	2.6301980867	5.6270218465	7.6669379579			
Ti	8.0913760000	0.0000000000	0.0000000000	0	0	0
Ti	7.9557249075	0.1002088581	7.5639420998			
Ti	8.0913760000	5.4974580000	0.0000000000	0	0	0
Ti	7.9801644443	5.5950717437	7.5586054324			
O	0.4505208029	2.6332604942	5.7429059851			
O	0.4729351785	8.1409531592	5.7544413968			
O	5.8712338745	2.6692214884	5.7512414064			
O	5.8825051835	8.1725788551	5.7428387736			
O	3.1238100000	0.1116490000	1.9196440000	0	0	0
O	3.1238100000	5.6091080000	1.9196440000	0	0	0
O	8.5180770000	0.1116490000	1.9196440000	0	0	0
O	8.5180770000	5.6091080000	1.9196440000	0	0	0
O	1.1347870628	1.2057562316	3.4758872112			
O	1.1344635056	6.7038445810	3.4860105258			
O	6.5249246011	1.2088661576	3.4905373827			
O	6.5296795431	6.7062748200	3.4887565768			
O	1.1266440000	1.1517870000	0.3184580000	0	0	0
O	1.1510724300	1.1531008837	7.9685552494			
O	1.1266440000	6.6492460000	0.3184580000	0	0	0

O	1.1716812263	6.6584830874	7.9964602791			
O	6.5209110000	1.1517870000	0.3184580000	0	0	0
O	6.5355045303	1.1516999914	7.9606467571			
O	6.5209110000	6.6492460000	0.3184580000	0	0	0
O	6.5620975412	6.6589906345	7.9635238182			
O	4.2752225779	4.3921201762	4.1690368070			
O	4.2633737067	9.8943569732	4.1770692583			
O	9.6647228486	4.3962346121	4.1917327203			
O	9.6517736358	9.8839897658	4.1861558439			
O	1.5619267820	3.9549084110	3.5036573211			
O	1.5609965246	9.4538241992	3.4888089411			
O	6.9658835915	3.9563381955	3.4860191328			
O	6.9541306475	9.4591034789	3.4706131236			
O	1.5704400000	3.9005160000	0.3184570000	0	0	0
O	1.5154362836	3.9116105551	7.8855085609			
O	1.5704400000	9.3979740000	0.3184570000	0	0	0
O	1.5510560618	9.3973710261	7.9678559561			
O	6.9647070000	3.9005160000	0.3184570000	0	0	0
O	6.9313786670	3.9098556089	7.9897173763			
O	6.9647070000	9.3979740000	0.3184570000	0	0	0
O	6.9515851001	9.3993069715	7.9833530005			
O	3.8256731084	1.6481073984	4.1881397790			
O	3.8352341154	7.1488317953	4.1740678948			
O	9.2170630090	1.6398849884	4.1685102126			
O	9.2171093204	7.1442435305	4.1705192643			
O	3.8857419993	1.4432987033	7.3854492447			
O	3.9223797383	6.9590372535	7.3783097196			
O	9.2793535763	1.4192563975	7.3911037618			
O	9.3096449005	6.9149441141	7.4012411016			
O	4.9675650000	2.8603790000	1.9196440000	0	0	0
O	4.9675650000	8.3578380000	1.9196440000	0	0	0
O	4.2123172247	4.1893555051	7.4033734814			
O	4.2057213142	9.6902162498	7.3906270440			
O	9.5726962791	4.1670212016	7.3975289442			
O	9.5966412789	9.6700965829	7.3918581898			
O	2.2877675447	5.4329827863	5.7631609407			
O	7.6183884895	5.4063279299	5.7511589626			
O	3.8237770000	1.5969400000	-0.3184580000	0	0	0
O	3.8237770000	7.0943990000	-0.3184580000	0	0	0
O	9.2180450000	1.5969400000	-0.3184580000	0	0	0
O	9.2180450000	7.0943990000	-0.3184580000	0	0	0
O	7.6144299464	-0.0992203917	5.7496736090			
O	-0.4267020000	2.8603790000	1.9196440000	0	0	0
O	-0.4267020000	8.3578380000	1.9196440000	0	0	0
O	4.2675730000	4.3456700000	-0.3184590000	0	0	0
O	4.2675730000	9.8431280000	-0.3184590000	0	0	0
O	9.6618410000	4.3456700000	-0.3184590000	0	0	0
O	9.6618410000	9.8431280000	-0.3184590000	0	0	0
O	2.2217821444	-0.0939312967	5.7495889583			
C	4.3365986313	6.0235538310	10.3744224820			
O	3.0603855237	5.6034463213	9.8349754149			
H	4.4242287070	5.7027293252	11.4214309397			
H	5.1101453192	5.5552594487	9.7587336985			
H	2.3377555281	6.1223786299	10.2357945027			
H	4.4449054117	7.1150157826	10.3035949329			

CH-c1

107
CH_c1

Ca	-0.1066421586	5.7532467158	5.7689734975			
Ca	2.8427158824	2.9347381520	5.7753696192			
Ca	2.7354032406	8.5049674000	5.6535335802			
Ca	8.1880218441	2.9435893738	5.7301513475			
Ca	8.2604869565	8.4883328527	5.7612472204			
Ca	2.6474700000	2.5105950000	1.9196440000	0	0	0
Ca	2.6474700000	8.0080540000	1.9196440000	0	0	0
Ca	8.0417380000	2.5105950000	1.9196440000	0	0	0
Ca	8.0417380000	8.0080540000	1.9196440000	0	0	0
Ca	5.3287202526	0.2980739244	5.6841701246			
Ca	5.2208706412	5.6974918230	5.8433337974			
Ca	10.6549148761	0.2126932413	5.7747155771			
Ca	0.0496100000	5.2593300000	1.9196450000	0	0	0
Ca	0.0496100000	10.7567880000	1.9196450000	0	0	0
Ca	5.4438780000	5.2593300000	1.9196450000	0	0	0
Ca	5.4438780000	10.7567880000	1.9196450000	0	0	0
Ti	-0.0047164377	2.7326390508	3.7833344043			
Ti	-0.0062909293	8.2251446790	3.7797633777			
Ti	5.4011748896	2.7573018042	3.7825996887			
Ti	5.4102929451	8.2261534520	3.8399285615			
Ti	0.0000000000	2.7487280000	0.0000000000	0	0	0
Ti	0.0647877162	2.7668664055	7.5498365073			
Ti	0.0000000000	8.2461860000	0.0000000000	0	0	0
Ti	0.1150584963	8.3516506460	7.5695960675			
Ti	5.3942680000	2.7487280000	0.0000000000	0	0	0
Ti	5.5146990133	2.8297013788	7.5681123868			
Ti	5.3942680000	8.2461860000	0.0000000000	0	0	0
Ti	5.7682131045	8.4814701654	7.7394324022			
Ti	2.7033961472	-0.0011987104	3.7817373494			
Ti	2.6976507192	5.4672329758	3.7939848705			
Ti	8.0967614564	-0.0248004004	3.7831551144			
Ti	8.0935020308	5.4790882928	3.7868916851			
Ti	2.6971090000	0.0000000000	0.0000000000	0	0	0
Ti	2.5192853762	0.1389158913	7.5693507244			
Ti	2.6971090000	5.4974580000	0.0000000000	0	0	0
Ti	2.3983834144	5.4083963144	7.6653271497			
Ti	8.0913760000	0.0000000000	0.0000000000	0	0	0
Ti	7.9937448837	0.1337555965	7.5766239618			
Ti	8.0913760000	5.4974580000	0.0000000000	0	0	0
Ti	8.0811307243	5.4438819342	7.5154439247			
O	0.4821256738	2.6614506415	5.7907193213			
O	0.4574203996	8.1247959969	5.7707562261			
O	5.8735930693	2.6603358803	5.7589977798			
O	5.8560045054	8.2266276130	5.8664978262			
O	3.1238100000	0.1116490000	1.9196440000	0	0	0
O	3.1238100000	5.6091080000	1.9196440000	0	0	0
O	8.5180770000	0.1116490000	1.9196440000	0	0	0
O	8.5180770000	5.6091080000	1.9196440000	0	0	0
O	1.1484634661	1.1759223426	3.5200378102			
O	1.1537415785	6.6649873175	3.5336882192			
O	6.5380121224	1.1684645542	3.5372206623			
O	6.5382655072	6.6818813221	3.5434132564			
O	1.1266440000	1.1517870000	0.3184580000	0	0	0
O	1.1007041576	1.1616534581	7.9947211675			
O	1.1266440000	6.6492460000	0.3184580000	0	0	0

O	1.0736565426	6.6137418103	7.9602349360			
O	6.5209110000	1.1517870000	0.3184580000	0	0	0
O	6.5462950002	1.1439106549	7.9569045891			
O	6.5209110000	6.6492460000	0.3184580000	0	0	0
O	6.2791006565	6.5683347982	8.0146855929			
O	4.2517234430	4.3201170066	4.1952688179			
O	4.2361368393	9.8181289727	4.2192067674			
O	9.6335884561	4.3145697798	4.2021713931			
O	9.6448303353	9.8081346839	4.1798558621			
O	1.5601926956	3.9229185967	3.5265335022			
O	1.5699083198	9.4027581602	3.5344127147			
O	6.9520857922	3.9213487407	3.4930726731			
O	6.9501675421	9.4204300129	3.5303125205			
O	1.5704400000	3.9005160000	0.3184570000	0	0	0
O	1.4439196863	3.8686334054	8.1405718693			
O	1.5704400000	9.3979740000	0.3184570000	0	0	0
O	1.5832437298	9.3480484113	8.0175867248			
O	6.9647070000	3.9005160000	0.3184570000	0	0	0
O	6.8858440345	3.9365903022	8.0355052627			
O	6.9647070000	9.3979740000	0.3184570000	0	0	0
O	7.1458582453	9.4714199493	8.2397217667			
O	3.8514305939	1.5646310864	4.1838596611			
O	3.8570466601	7.0568277469	4.2562064897			
O	9.2557450042	1.5594606910	4.1932569276			
O	9.2453194309	7.0576828050	4.1930127674			
O	3.8995478895	1.3314533793	7.3328760946			
O	3.9495535170	6.6149260543	7.8632270258			
O	9.2985974808	1.5047753333	7.3891913273			
O	9.1509911709	6.9585166413	7.4103622741			
O	4.9675650000	2.8603790000	1.9196440000	0	0	0
O	4.9675650000	8.3578380000	1.9196440000	0	0	0
O	4.1327920629	4.0489595901	7.3902442202			
O	4.0726764739	9.5435609113	7.7811352384			
O	9.5297684192	4.2320520151	7.3279484761			
O	9.6544740903	9.7148091901	7.3459099676			
O	2.2458063913	5.3719255218	5.8337380265			
O	7.5748811985	5.3669148307	5.7714785392			
O	3.8237770000	1.5969400000	-0.3184580000	0	0	0
O	3.8237770000	7.0943990000	-0.3184580000	0	0	0
O	9.2180450000	1.5969400000	-0.3184580000	0	0	0
O	9.2180450000	7.0943990000	-0.3184580000	0	0	0
O	7.6360649038	-0.1141544699	5.7891224048			
O	-0.4267020000	2.8603790000	1.9196440000	0	0	0
O	-0.4267020000	8.3578380000	1.9196440000	0	0	0
O	4.2675730000	4.3456700000	-0.3184590000	0	0	0
O	4.2675730000	9.8431280000	-0.3184590000	0	0	0
O	9.6618410000	4.3456700000	-0.3184590000	0	0	0
O	9.6618410000	9.8431280000	-0.3184590000	0	0	0
O	2.2044043877	-0.1378946072	5.7825557805			
C	5.0525071501	6.8195276488	8.7085454936			
H	4.9888637244	6.3278610938	9.6855114580			
H	3.3638972340	8.9926426734	8.2118231041			

*CH-c2

108
CaTiO3

Ca	-0.1063796559	5.7699072995	5.7277867578			
Ca	2.8050603374	2.9619312581	5.7364376211			
Ca	2.8698022610	8.4762180442	5.8571868840			
Ca	8.1953457854	2.9622030029	5.7170282759			
Ca	8.2009131180	8.5175167508	5.8110113694			
Ca	2.6474700000	2.5105950000	1.9196440000	0	0	0
Ca	2.6474700000	8.0080540000	1.9196440000	0	0	0
Ca	8.0417380000	2.5105950000	1.9196440000	0	0	0
Ca	8.0417380000	8.0080540000	1.9196440000	0	0	0
Ca	5.2818773621	0.2528925480	5.7295543057			
Ca	5.2457748559	5.7230177600	5.7783304152			
Ca	10.6631282078	0.2314207340	5.7558944362			
Ca	0.0496100000	5.2593300000	1.9196450000	0	0	0
Ca	0.0496100000	10.7567880000	1.9196450000	0	0	0
Ca	5.4438780000	5.2593300000	1.9196450000	0	0	0
Ca	5.4438780000	10.7567880000	1.9196450000	0	0	0
Ti	0.0007723267	2.7345659489	3.7739811288			
Ti	-0.0033248499	8.2379133881	3.7881873291			
Ti	5.3872005638	2.7353011540	3.7811384918			
Ti	5.4032135694	8.2216521940	3.8404247204			
Ti	0.0000000000	2.7487280000	0.0000000000	0	0	0
Ti	0.1102912698	2.7591813692	7.5592173947			
Ti	0.0000000000	8.2461860000	0.0000000000	0	0	0
Ti	0.1335789570	8.3868760714	7.5305210890			
Ti	5.3942680000	2.7487280000	0.0000000000	0	0	0
Ti	5.3400191327	2.7546339541	7.5468794193			
Ti	5.3942680000	8.2461860000	0.0000000000	0	0	0
Ti	5.5661604274	8.5752491012	7.6757684040			
Ti	2.6929453918	-0.0344936023	3.7809570498			
Ti	2.6945645966	5.4851401712	3.7836229136			
Ti	8.0933871678	-0.0152241935	3.7877210308			
Ti	8.0904704432	5.4881202034	3.7935864825			
Ti	2.6971090000	0.0000000000	0.0000000000	0	0	0
Ti	2.5378116327	0.0607843723	7.5719584275			
Ti	2.6971090000	5.4974580000	0.0000000000	0	0	0
Ti	2.3973139277	5.4574337669	7.6056562714			
Ti	8.0913760000	0.0000000000	0.0000000000	0	0	0
Ti	7.9855334330	0.1625424868	7.5673080814			
Ti	8.0913760000	5.4974580000	0.0000000000	0	0	0
Ti	8.1839016250	5.5309653474	7.5666605365			
O	0.4776882703	2.6598467455	5.7898941767			
O	0.5297290848	8.1632151054	5.7591818035			
O	5.8547988862	2.6623596317	5.7968840123			
O	5.8743770382	8.0859087099	5.8511060325			
O	3.1238100000	0.1116490000	1.9196440000	0	0	0
O	3.1238100000	5.6091080000	1.9196440000	0	0	0
O	8.5180770000	0.1116490000	1.9196440000	0	0	0
O	8.5180770000	5.6091080000	1.9196440000	0	0	0
O	1.1480126933	1.1810604515	3.5254328701			
O	1.1338096837	6.6632520905	3.5228358586			
O	6.5349996055	1.1799299272	3.5173635238			
O	6.5334906325	6.6607555946	3.5321500868			
O	1.1266440000	1.1517870000	0.3184580000	0	0	0
O	1.1619284985	1.1681601525	8.0055235355			
O	1.1266440000	6.6492460000	0.3184580000	0	0	0

O	1.1061980620	6.6356415566	7.9880178464			
O	6.5209110000	1.1517870000	0.3184580000	0	0	0
O	6.6068128040	1.2141917871	8.0545847107			
O	6.5209110000	6.6492460000	0.3184580000	0	0	0
O	6.7283746625	6.5375680007	8.3865760150			
O	4.2377211598	4.3162083915	4.1978608679			
O	4.2326321496	9.8019417671	4.2155698746			
O	9.6320710001	4.3201621789	4.2062902590			
O	9.6346996702	9.8159727129	4.1942882540			
O	1.5555242421	3.9199050973	3.5128035054			
O	1.5451261634	9.4308509284	3.4972138956			
O	6.9545298881	3.9205403164	3.5376618880			
O	6.9449147284	9.4194108849	3.5557434133			
O	1.5704400000	3.9005160000	0.3184570000	0	0	0
O	1.5320353281	3.8665769726	8.0753021678			
O	1.5704400000	9.3979740000	0.3184570000	0	0	0
O	1.6248492775	9.3971981702	7.9808491286			
O	6.9647070000	3.9005160000	0.3184570000	0	0	0
O	6.9894321989	3.8178174373	8.2574444619			
O	6.9647070000	9.3979740000	0.3184570000	0	0	0
O	7.0879438389	9.4757916543	7.9721138360			
O	3.8580548628	1.5646746869	4.1937715243			
O	3.8357952272	7.0559647358	4.2298009373			
O	9.2547914925	1.5732795497	4.1789613438			
O	9.2338535109	7.0688863319	4.2180640571			
O	3.9225672149	1.4932832846	7.3788405582			
O	3.8594868614	6.8911924178	7.4474135074			
O	9.3241452299	1.5296769155	7.4089548054			
O	9.2920937543	6.9910959536	7.3292690726			
O	4.9675650000	2.8603790000	1.9196440000	0	0	0
O	4.9675650000	8.3578380000	1.9196440000	0	0	0
O	4.2491295714	4.2219173393	7.4299678319			
O	4.2471293026	9.8081947106	7.4173186562			
O	9.5737385200	4.2355092764	7.4262470021			
O	9.6758940116	9.7416326462	7.3524970121			
O	2.1964678831	5.3443703594	5.7993099055			
O	7.5808257724	5.3551230695	5.8505272174			
O	3.8237770000	1.5969400000	-0.3184580000	0	0	0
O	3.8237770000	7.0943990000	-0.3184580000	0	0	0
O	9.2180450000	1.5969400000	-0.3184580000	0	0	0
O	9.2180450000	7.0943990000	-0.3184580000	0	0	0
O	7.6362331301	-0.0110617740	5.7732760278			
O	-0.4267020000	2.8603790000	1.9196440000	0	0	0
O	-0.4267020000	8.3578380000	1.9196440000	0	0	0
O	4.2675730000	4.3456700000	-0.3184590000	0	0	0
O	4.2675730000	9.8431280000	-0.3184590000	0	0	0
O	9.6618410000	4.3456700000	-0.3184590000	0	0	0
O	9.6618410000	9.8431280000	-0.3184590000	0	0	0
O	2.2231701270	-0.0846680313	5.7848413457			
C	5.8727395372	7.1450857246	9.2134722329			
H	6.0044453168	6.9273967735	10.2857943636			
H	7.5786905196	3.1019519043	8.5716156202			
H	4.3534786954	6.8932395075	8.3485250088			

CH-c3

107
CH_c3

Ca	-0.1428996603	5.7635351584	5.7792186722			
Ca	2.8697802999	2.9764648467	5.9077154191			
Ca	2.8347250866	8.5081754472	5.8379929330			
Ca	8.2308385138	3.0066082004	5.7552903742			
Ca	8.2286828825	8.4947926083	5.7662503885			
Ca	2.6474700000	2.5105950000	1.9196440000	0	0	0
Ca	2.6474700000	8.0080540000	1.9196440000	0	0	0
Ca	8.0417380000	2.5105950000	1.9196440000	0	0	0
Ca	8.0417380000	8.0080540000	1.9196440000	0	0	0
Ca	5.2612162878	0.1951766273	5.7953067707			
Ca	5.2592148668	5.7468959645	5.8055475684			
Ca	10.7346635161	0.2371185393	5.7783214777			
Ca	0.0496100000	5.2593300000	1.9196450000	0	0	0
Ca	0.0496100000	10.7567880000	1.9196450000	0	0	0
Ca	5.4438780000	5.2593300000	1.9196450000	0	0	0
Ca	5.4438780000	10.7567880000	1.9196450000	0	0	0
Ti	0.0159671270	2.7383060010	3.7966301884			
Ti	0.0095740207	8.2321394265	3.7963647413			
Ti	5.3934429238	2.7213879649	3.8036118025			
Ti	5.3939736111	8.2231823280	3.7995332112			
Ti	0.0000000000	2.7487280000	0.0000000000	0	0	0
Ti	0.0354754717	2.8255129957	7.5241060581			
Ti	0.0000000000	8.2461860000	0.0000000000	0	0	0
Ti	0.1292215734	8.3903194564	7.5420634533			
Ti	5.3942680000	2.7487280000	0.0000000000	0	0	0
Ti	5.7103552854	2.6648038621	7.6098429197			
Ti	5.3942680000	8.2461860000	0.0000000000	0	0	0
Ti	5.6296728743	8.4129300771	7.5802901631			
Ti	2.7017841673	-0.0171169719	3.8009752512			
Ti	2.6707711358	5.4676051139	3.8883086475			
Ti	8.0996994552	-0.0116925423	3.7945130993			
Ti	8.0819115147	5.4789664396	3.7964607421			
Ti	2.6971090000	0.0000000000	0.0000000000	0	0	0
Ti	2.6045502963	0.1163009276	7.5738912919			
Ti	2.6971090000	5.4974580000	0.0000000000	0	0	0
Ti	2.2605288611	5.4961172114	7.7842441744			
Ti	8.0913760000	0.0000000000	0.0000000000	0	0	0
Ti	8.0690935453	0.0713767247	7.5287695616			
Ti	8.0913760000	5.4974580000	0.0000000000	0	0	0
Ti	7.9511288401	5.6301467853	7.5280343514			
O	0.5126351967	2.6699122562	5.7566349555			
O	0.4909839438	8.1708302790	5.7494396907			
O	5.9162909427	2.5784386810	5.7918922804			
O	5.8748927839	8.1523919108	5.7679591107			
O	3.1238100000	0.1116490000	1.9196440000	0	0	0
O	3.1238100000	5.6091080000	1.9196440000	0	0	0
O	8.5180770000	0.1116490000	1.9196440000	0	0	0
O	8.5180770000	5.6091080000	1.9196440000	0	0	0
O	1.1466287245	1.1784496018	3.5073846659			
O	1.1414988967	6.6741633600	3.5274360735			
O	6.5419318912	1.1789103056	3.4921694454			
O	6.5346290212	6.6801094402	3.4925608693			
O	1.1266440000	1.1517870000	0.3184580000	0	0	0
O	1.1783334112	1.1391288756	7.9290412974			
O	1.1266440000	6.6492460000	0.3184580000	0	0	0

O	0.9023719072	6.6376012790	7.9898358483			
O	6.5209110000	1.1517870000	0.3184580000	0	0	0
O	6.6171063865	1.0927956856	8.0885703449			
O	6.5209110000	6.6492460000	0.3184580000	0	0	0
O	6.4736222360	6.6158597919	7.9587116408			
O	4.2528777727	4.3004366454	4.2389131243			
O	4.2497613895	9.8131256849	4.1746984288			
O	9.6358408567	4.3222100635	4.1832139017			
O	9.6450973754	9.8254609713	4.1869555523			
O	1.5512410029	3.9359057801	3.4882523385			
O	1.5535943298	9.4327011928	3.4954352745			
O	6.9456381576	3.9198394034	3.4996139697			
O	6.9574736736	9.4283882710	3.5135965912			
O	1.5704400000	3.9005160000	0.3184570000	0	0	0
O	1.5024762193	3.8407799173	8.1285132642			
O	1.5704400000	9.3979740000	0.3184570000	0	0	0
O	1.5915265173	9.3660273670	7.9530235673			
O	6.9647070000	3.9005160000	0.3184570000	0	0	0
O	6.9936758887	3.8585385684	7.9973353047			
O	6.9647070000	9.3979740000	0.3184570000	0	0	0
O	7.0675685706	9.3935645840	7.9782882934			
O	3.8523173838	1.5739005288	4.2072180836			
O	3.8545051252	7.0693489405	4.2275368386			
O	9.2554896051	1.5736676378	4.1859742248			
O	9.2397143770	7.0701456325	4.1811976611			
O	3.9389897765	1.4421453853	7.3950196417			
O	3.8684528566	7.0065365401	7.5325440803			
O	9.3256305078	1.4837670399	7.3530171237			
O	9.1878094961	6.9795106667	7.3029262301			
O	4.9675650000	2.8603790000	1.9196440000	0	0	0
O	4.9675650000	8.3578380000	1.9196440000	0	0	0
O	4.3179617173	4.1995713719	7.4990258859			
O	4.2754656509	9.6878606043	7.4100309913			
O	9.6119983619	4.2143112979	7.3237800961			
O	9.6240663001	9.7570090307	7.3539717160			
O	2.2396145819	5.3768746141	5.8184840391			
O	7.5689726963	5.3753896083	5.7364150688			
O	3.8237770000	1.5969400000	-0.3184580000	0	0	0
O	3.8237770000	7.0943990000	-0.3184580000	0	0	0
O	9.2180450000	1.5969400000	-0.3184580000	0	0	0
O	9.2180450000	7.0943990000	-0.3184580000	0	0	0
O	7.6227013446	-0.0737674700	5.7636204440			
O	-0.4267020000	2.8603790000	1.9196440000	0	0	0
O	-0.4267020000	8.3578380000	1.9196440000	0	0	0
O	4.2675730000	4.3456700000	-0.3184590000	0	0	0
O	4.2675730000	9.8431280000	-0.3184590000	0	0	0
O	9.6618410000	4.3456700000	-0.3184590000	0	0	0
O	9.6618410000	9.8431280000	-0.3184590000	0	0	0
O	2.2738016262	-0.1124906993	5.7595213335			
C	3.6566362827	6.5653178747	8.8119651325			
H	4.0356603908	7.2010027378	9.6290340205			
H	4.5040061073	4.8059600144	8.2665834636			

*CH-c4

108
CH_c4

Ca	-0.1249903275	5.7527566766	5.7505874911			
Ca	2.8536298225	2.9633358279	5.7764594552			
Ca	2.8467572691	8.5385105739	5.7569766748			
Ca	8.1988944490	2.9193823780	5.7497803314			
Ca	8.1647027415	8.4886322500	5.8651587509			
Ca	2.6474700000	2.5105950000	1.9196440000	0	0	0
Ca	2.6474700000	8.0080540000	1.9196440000	0	0	0
Ca	8.0417380000	2.5105950000	1.9196440000	0	0	0
Ca	8.0417380000	8.0080540000	1.9196440000	0	0	0
Ca	5.2921385636	0.2686271762	5.8105899715			
Ca	5.2640285058	5.7739191363	5.8512083732			
Ca	10.6727939987	0.1905164505	5.7972996222			
Ca	0.0496100000	5.2593300000	1.9196450000	0	0	0
Ca	0.0496100000	10.7567880000	1.9196450000	0	0	0
Ca	5.4438780000	5.2593300000	1.9196450000	0	0	0
Ca	5.4438780000	10.7567880000	1.9196450000	0	0	0
Ti	0.0124211128	2.7302958747	3.7891901170			
Ti	-0.0124647030	8.2215583396	3.8077217272			
Ti	5.3961115499	2.7388753527	3.7962885138			
Ti	5.4303994862	8.2317877826	3.9240307122			
Ti	0.0000000000	2.7487280000	0.0000000000	0	0	0
Ti	0.1079902877	2.7145970433	7.5483676849			
Ti	0.0000000000	8.2461860000	0.0000000000	0	0	0
Ti	0.1625033571	8.3675088131	7.5468857580			
Ti	5.3942680000	2.7487280000	0.0000000000	0	0	0
Ti	5.4966761228	2.9020757840	7.5766732011			
Ti	5.3942680000	8.2461860000	0.0000000000	0	0	0
Ti	5.5063307895	8.5358349670	7.8828974941			
Ti	2.7005810749	-0.0199711140	3.7863794593			
Ti	2.7056729938	5.4848530365	3.7982441179			
Ti	8.0992658703	-0.0283372162	3.7994063905			
Ti	8.0745195418	5.4747262724	3.7913821957			
Ti	2.6971090000	0.0000000000	0.0000000000	0	0	0
Ti	2.6154003903	0.1217414809	7.5439904063			
Ti	2.6971090000	5.4974580000	0.0000000000	0	0	0
Ti	2.3854026147	5.4149331795	7.6093042104			
Ti	8.0913760000	0.0000000000	0.0000000000	0	0	0
Ti	7.9663926409	0.1451061912	7.5647006801			
Ti	8.0913760000	5.4974580000	0.0000000000	0	0	0
Ti	8.1308798933	5.4562776378	7.5010651886			
O	0.5023597762	2.6270922210	5.7753584972			
O	0.5089465292	8.1349505893	5.7469641531			
O	5.8520423365	2.7136695870	5.7744926347			
O	5.8139428946	8.0982644645	5.7956369545			
O	3.1238100000	0.1116490000	1.9196440000	0	0	0
O	3.1238100000	5.6091080000	1.9196440000	0	0	0
O	8.5180770000	0.1116490000	1.9196440000	0	0	0
O	8.5180770000	5.6091080000	1.9196440000	0	0	0
O	1.1552637685	1.1849376923	3.4863747718			
O	1.1336528613	6.6633027855	3.5118871777			
O	6.5483388402	1.1900333305	3.5211848644			
O	6.5396333629	6.6597590575	3.5160158220			
O	1.1266440000	1.1517870000	0.3184580000	0	0	0
O	1.1309318695	1.1179395122	7.9851219074			
O	1.1266440000	6.6492460000	0.3184580000	0	0	0

O	1.0436084718	6.5814858280	7.9787822023			
O	6.5209110000	1.1517870000	0.3184580000	0	0	0
O	6.5535290982	1.1954871831	7.9436735338			
O	6.5209110000	6.6492460000	0.3184580000	0	0	0
O	6.4406172861	6.5642181816	8.0341830037			
O	4.2516612786	4.3371963000	4.1996057501			
O	4.2476747957	9.8297471063	4.1944045397			
O	9.6430561972	4.3083105547	4.1895139786			
O	9.6417392877	9.8122535780	4.1864522186			
O	1.5596664128	3.9201305943	3.5034137418			
O	1.5604349234	9.4292880263	3.5121690633			
O	6.9561284304	3.9206554481	3.4813353263			
O	6.9586721562	9.4212474470	3.5367650703			
O	1.5704400000	3.9005160000	0.3184570000	0	0	0
O	1.4859913885	3.8413185035	8.0955581316			
O	1.5704400000	9.3979740000	0.3184570000	0	0	0
O	1.6513704342	9.3250476926	7.9522439128			
O	6.9647070000	3.9005160000	0.3184570000	0	0	0
O	6.8830069597	3.9458824927	8.0771597252			
O	6.9647070000	9.3979740000	0.3184570000	0	0	0
O	7.0548638483	9.4461587523	7.9682639787			
O	3.8614432735	1.5789242272	4.1802597574			
O	3.8228638389	7.0526890082	4.2201293593			
O	9.2696617499	1.5598801605	4.1804580270			
O	9.2334562085	7.0696707979	4.2039885885			
O	3.8725107685	1.4947264709	7.3835362589			
O	3.6743359587	7.0526622765	7.6168179149			
O	9.3160188794	1.4783006744	7.3833042522			
O	9.1966220963	6.9358346057	7.3492544509			
O	4.9675650000	2.8603790000	1.9196440000	0	0	0
O	4.9675650000	8.3578380000	1.9196440000	0	0	0
O	4.1712289724	4.2774134099	7.4150940296			
O	4.2361617350	9.7904046792	7.3370691973			
O	9.5522510827	4.1929187450	7.3411402509			
O	9.6667092503	9.6939935244	7.3659143678			
O	2.1815282558	5.3245811722	5.7964434948			
O	7.6018915769	5.3336316307	5.7564466301			
O	3.8237770000	1.5969400000	-0.3184580000	0	0	0
O	3.8237770000	7.0943990000	-0.3184580000	0	0	0
O	9.2180450000	1.5969400000	-0.3184580000	0	0	0
O	9.2180450000	7.0943990000	-0.3184580000	0	0	0
O	7.6437891333	-0.0503738229	5.7613997268			
O	-0.4267020000	2.8603790000	1.9196440000	0	0	0
O	-0.4267020000	8.3578380000	1.9196440000	0	0	0
O	4.2675730000	4.3456700000	-0.3184590000	0	0	0
O	4.2675730000	9.8431280000	-0.3184590000	0	0	0
O	9.6618410000	4.3456700000	-0.3184590000	0	0	0
O	9.6618410000	9.8431280000	-0.3184590000	0	0	0
O	2.2237756619	-0.0652963644	5.7566395552			
C	4.9818167232	8.3775343927	9.8498614724			
H	4.6458639531	8.1569744935	10.8738022375			
H	3.1403619839	7.6882241614	8.1540120720			
H	5.8800225201	6.0092029103	8.6111264348			

*CH_2-c2

109
CH2_c2

Ca	-0.0593148615	5.7150992590	5.6810166426			
Ca	2.8229520473	2.9958458765	5.7826166190			
Ca	2.7978566905	8.5010675524	5.7636021468			
Ca	8.2224547240	2.9358806579	5.7001600322			
Ca	8.1759427494	8.5153830173	5.7083968231			
Ca	2.6474700000	2.5105950000	1.9196440000	0	0	0
Ca	2.6474700000	8.0080540000	1.9196440000	0	0	0
Ca	8.0417380000	2.5105950000	1.9196440000	0	0	0
Ca	8.0417380000	8.0080540000	1.9196440000	0	0	0
Ca	5.2896388513	0.2160184921	5.7185434139			
Ca	5.2247933344	5.7437366514	5.7193941590			
Ca	10.6730175091	0.2426357957	5.7586034259			
Ca	0.0496100000	5.2593300000	1.9196450000	0	0	0
Ca	0.0496100000	10.7567880000	1.9196450000	0	0	0
Ca	5.4438780000	5.2593300000	1.9196450000	0	0	0
Ca	5.4438780000	10.7567880000	1.9196450000	0	0	0
Ti	0.0093795262	2.7267560018	3.7745365000			
Ti	-0.0074303482	8.2427780278	3.7868838424			
Ti	5.3861838390	2.7255760207	3.7826663833			
Ti	5.3806667642	8.2245094912	3.7786899282			
Ti	0.0000000000	2.7487280000	0.0000000000	0	0	0
Ti	0.1775412144	2.7989427326	7.5814685898			
Ti	0.0000000000	8.2461860000	0.0000000000	0	0	0
Ti	0.1668991242	8.4113813232	7.6058788250			
Ti	5.3942680000	2.7487280000	0.0000000000	0	0	0
Ti	5.5802203567	2.5830445560	7.6343926572			
Ti	5.3942680000	8.2461860000	0.0000000000	0	0	0
Ti	5.4427488032	8.4066493096	7.5731132170			
Ti	2.6984168567	-0.0094987605	3.7792719023			
Ti	2.6956113619	5.4846585956	3.7750579814			
Ti	8.0988761752	-0.0117786186	3.7757505904			
Ti	8.0915488815	5.4845870835	3.7856108553			
Ti	2.6971090000	0.0000000000	0.0000000000	0	0	0
Ti	2.5855169254	0.1229729449	7.5600816146			
Ti	2.6971090000	5.4974580000	0.0000000000	0	0	0
Ti	2.4750270554	5.6693134595	7.5997421980			
Ti	8.0913760000	0.0000000000	0.0000000000	0	0	0
Ti	8.0738349120	0.0520451534	7.5523904034			
Ti	8.0913760000	5.4974580000	0.0000000000	0	0	0
Ti	8.0255897165	5.2928015410	7.6366921933			
O	0.4786386426	2.6698411415	5.7930454929			
O	0.4141212762	8.1588381151	5.8188795395			
O	5.8767449140	2.6132078598	5.8493816958			
O	5.8719827373	8.0622913923	5.8363084901			
O	3.1238100000	0.1116490000	1.9196440000	0	0	0
O	3.1238100000	5.6091080000	1.9196440000	0	0	0
O	8.5180770000	0.1116490000	1.9196440000	0	0	0
O	8.5180770000	5.6091080000	1.9196440000	0	0	0
O	1.1486557899	1.1728446182	3.5140216751			
O	1.1268275226	6.6565533695	3.5505344840			
O	6.5332014834	1.1708470654	3.5313784419			
O	6.5244706714	6.6543800168	3.5431490298			
O	1.1266440000	1.1517870000	0.3184580000	0	0	0
O	1.1515945639	1.1947748044	7.9965117632			
O	1.1266440000	6.6492460000	0.3184580000	0	0	0

O	1.0041606883	6.6757103592	7.9635693723			
O	6.5209110000	1.1517870000	0.3184580000	0	0	0
O	6.5488518016	1.0918470934	8.0778930523			
O	6.5209110000	6.6492460000	0.3184580000	0	0	0
O	6.3918446655	6.5901639132	8.4733167537			
O	4.2418926616	4.3074020023	4.2155991846			
O	4.2487978551	9.8041137535	4.2002116767			
O	9.6230370677	4.3140773947	4.2062966841			
O	9.6401595534	9.8166213170	4.1829304192			
O	1.5536435566	3.9257303020	3.5397201030			
O	1.5607905522	9.4071219878	3.5588042669			
O	6.9515984315	3.9139242464	3.5740856305			
O	6.9543862275	9.4001534320	3.5656070108			
O	1.5704400000	3.9005160000	0.3184570000	0	0	0
O	1.5268024199	3.9473737553	7.9870711977			
O	1.5704400000	9.3979740000	0.3184570000	0	0	0
O	1.5858239065	9.4450002698	8.0019284434			
O	6.9647070000	3.9005160000	0.3184570000	0	0	0
O	6.9180421376	3.8942994040	8.1290307021			
O	6.9647070000	9.3979740000	0.3184570000	0	0	0
O	6.9859413508	9.2961078147	7.9956582346			
O	3.8492377016	1.5715112759	4.2238047981			
O	3.8344854448	7.0694721388	4.2085704636			
O	9.2565649000	1.5562820533	4.1960704628			
O	9.2310815310	7.0543386947	4.2398992494			
O	3.9020714981	1.5338934452	7.4136627664			
O	3.8444255078	6.9832528325	7.4412817237			
O	9.2625898587	1.4959615145	7.3798991081			
O	9.0035931665	7.1320444362	7.8075537647			
O	4.9675650000	2.8603790000	1.9196440000	0	0	0
O	4.9675650000	8.3578380000	1.9196440000	0	0	0
O	4.2778312833	4.3108145977	7.6568403415			
O	4.2301618553	9.7684352529	7.3553888676			
O	9.5698815401	4.3099837096	7.3854954125			
O	9.5885427914	9.7509710013	7.4103746865			
O	2.2606413993	5.3998683672	5.8104521241			
O	7.5812465579	5.4159497956	5.8930692779			
O	3.8237770000	1.5969400000	-0.3184580000	0	0	0
O	3.8237770000	7.0943990000	-0.3184580000	0	0	0
O	9.2180450000	1.5969400000	-0.3184580000	0	0	0
O	9.2180450000	7.0943990000	-0.3184580000	0	0	0
O	7.6327910926	-0.0926498366	5.7927703072			
O	-0.4267020000	2.8603790000	1.9196440000	0	0	0
O	-0.4267020000	8.3578380000	1.9196440000	0	0	0
O	4.2675730000	4.3456700000	-0.3184590000	0	0	0
O	4.2675730000	9.8431280000	-0.3184590000	0	0	0
O	9.6618410000	4.3456700000	-0.3184590000	0	0	0
O	9.6618410000	9.8431280000	-0.3184590000	0	0	0
O	2.2288953526	-0.0591943261	5.7935724809			
C	5.7772658600	6.1469644225	9.5906132566			
H	5.1151604547	6.8320585293	10.1290280169			
H	6.2025101444	5.2868356561	10.1121302882			
H	8.3748305930	7.7945065494	8.1933260345			
H	4.7811901183	4.8854937598	8.2924088062			

CH$_2$-c3

```
108
CH2_c3
Ca            -0.0705536223       5.7503443672       5.7748525470
Ca             2.8420182641       2.9840742129       5.8436012736
Ca             2.8071840104       8.5176554015       5.8180864434
Ca             8.1775592292       2.9089894695       5.8047522996
Ca             8.1790766705       8.4845343408       5.8621170787
Ca             2.6474700000       2.5105950000       1.9196440000    0    0    0
Ca             2.6474700000       8.0080540000       1.9196440000    0    0    0
Ca             8.0417380000       2.5105950000       1.9196440000    0    0    0
Ca             8.0417380000       8.0080540000       1.9196440000    0    0    0
Ca             5.2928919520       0.2901451242       5.8292474161
Ca             5.2660534886       5.7255426334       5.8680913876
Ca            10.6532611907       0.1857360678       5.8365550780
Ca             0.0496100000       5.2593300000       1.9196450000    0    0    0
Ca             0.0496100000      10.7567880000       1.9196450000    0    0    0
Ca             5.4438780000       5.2593300000       1.9196450000    0    0    0
Ca             5.4438780000      10.7567880000       1.9196450000    0    0    0
Ti            -0.0062214585       2.6631655476       3.7811418055
Ti            -0.0446755305       8.1901697217       3.7963200981
Ti             5.3589890012       2.7071554332       3.7930625612
Ti             5.3918797804       8.1556488441       3.8566224549
Ti             0.0000000000       2.7487280000       0.0000000000    0    0    0
Ti             0.0962636939       2.7358306539       7.5400927975
Ti             0.0000000000       8.2461860000       0.0000000000    0    0    0
Ti             0.1401653745       8.3870698617       7.5507159888
Ti             5.3942680000       2.7487280000       0.0000000000    0    0    0
Ti             5.5099680462       2.8994224904       7.5924535237
Ti             5.3942680000       8.2461860000       0.0000000000    0    0    0
Ti             5.4708541229       8.4806443535       7.6725301475
Ti             2.7147559840      -0.0788444637       3.7838563657
Ti             2.6943155877       5.4377119566       3.7911132531
Ti             8.0801560616      -0.0725947026       3.7836928593
Ti             8.1034046249       5.4077825269       3.7847621011
Ti             2.6971090000       0.0000000000       0.0000000000    0    0    0
Ti             2.5903785198       0.1415849944       7.5372510391
Ti             2.6971090000       5.4974580000       0.0000000000    0    0    0
Ti             2.4302403718       5.4741472059       7.6486269083
Ti             8.0913760000       0.0000000000       0.0000000000    0    0    0
Ti             7.9486196006       0.1104763387       7.5769235296
Ti             8.0913760000       5.4974580000       0.0000000000    0    0    0
Ti             8.1538450576       5.4823932134       7.4915207210
O              0.4883519746       2.6351757756       5.7652813113
O              0.4843817260       8.1707499799       5.7508582282
O              5.8366653590       2.7328803716       5.7767932043
O              5.8555792532       8.0867781401       5.7927456099
O              3.1238100000       0.1116490000       1.9196440000    0    0    0
O              3.1238100000       5.6091080000       1.9196440000    0    0    0
O              8.5180770000       0.1116490000       1.9196440000    0    0    0
O              8.5180770000       5.6091080000       1.9196440000    0    0    0
O              1.1475997653       1.2055034987       3.4795350092
O              1.1358638074       6.6853647979       3.5296118900
O              6.5434627212       1.2084610368       3.5346211693
O              6.5367110993       6.6892648498       3.5089392506
O              1.1266440000       1.1517870000       0.3184580000    0    0    0
O              1.1277370093       1.1575621431       7.9910347635
O              1.1266440000       6.6492460000       0.3184580000    0    0    0
```

O	1.0876214991	6.6388212158	7.9736072881			
O	6.5209110000	1.1517870000	0.3184580000	0	0	0
O	6.5481477266	1.1959879816	7.9455774444			
O	6.5209110000	6.6492460000	0.3184580000	0	0	0
O	6.5529846874	6.5808581594	8.1165254766			
O	4.2701892913	4.3846998421	4.1809307633			
O	4.2601435156	9.8794640550	4.2037670353			
O	9.6560048979	4.3696259992	4.1837483146			
O	9.6545634414	9.8616262038	4.1719912045			
O	1.5632415811	3.9478782817	3.5128745722			
O	1.5737707940	9.4478800696	3.5054225656			
O	6.9655739118	3.9419648969	3.4846865727			
O	6.9566857238	9.4408779452	3.5198744434			
O	1.5704400000	3.9005160000	0.3184570000	0	0	0
O	1.4929139006	3.8814854569	8.0261225920			
O	1.5704400000	9.3979740000	0.3184570000	0	0	0
O	1.5889141237	9.4032209642	7.9418909616			
O	6.9647070000	3.9005160000	0.3184570000	0	0	0
O	6.9116285506	3.9516555028	8.0449257618			
O	6.9647070000	9.3979740000	0.3184570000	0	0	0
O	6.9850621683	9.4408168786	8.0149504497			
O	3.8421936457	1.6339508578	4.1774001115			
O	3.8241086914	7.1229810333	4.1977589465			
O	9.2520965631	1.6152737891	4.1815317721			
O	9.2300014216	7.1183268977	4.1899576831			
O	3.8731603734	1.4895849773	7.3878210543			
O	3.8398240333	6.9951163176	7.4134310998			
O	9.3054117535	1.4753785175	7.3897090890			
O	9.2427094561	6.9481518872	7.3352451449			
O	4.9675650000	2.8603790000	1.9196440000	0	0	0
O	4.9675650000	8.3578380000	1.9196440000	0	0	0
O	4.1865523277	4.2508207209	7.4743113736			
O	4.2141311582	9.7847428440	7.3290007375			
O	9.5630623222	4.2034825198	7.3385793218			
O	9.6143050784	9.6992014088	7.3850336369			
O	2.2493360572	5.3799648783	5.8073340901			
O	7.6096412722	5.3656688761	5.7525599062			
O	3.8237770000	1.5969400000	-0.3184580000	0	0	0
O	3.8237770000	7.0943990000	-0.3184580000	0	0	0
O	9.2180450000	1.5969400000	-0.3184580000	0	0	0
O	9.2180450000	7.0943990000	-0.3184580000	0	0	0
O	7.6374680773	-0.0676180077	5.7693377524			
O	-0.4267020000	2.8603790000	1.9196440000	0	0	0
O	-0.4267020000	8.3578380000	1.9196440000	0	0	0
O	4.2675730000	4.3456700000	-0.3184590000	0	0	0
O	4.2675730000	9.8431280000	-0.3184590000	0	0	0
O	9.6618410000	4.3456700000	-0.3184590000	0	0	0
O	9.6618410000	9.8431280000	-0.3184590000	0	0	0
O	2.2134961843	-0.0453087699	5.7480744189			
C	3.9811659123	7.3620435272	8.8081588935			
H	3.1049524356	7.9108394448	9.1709278480			
H	4.2804476840	6.5076327453	9.4424441633			
H	6.0028910417	5.9766276211	8.6508599530			

*CH_2-c4

109
CH2_c4

Ca	-0.0928659362	5.7273050447	5.8341872627			
Ca	2.8268022503	2.9928429176	5.8619789101			
Ca	2.9337155290	8.4752447618	5.9155189422			
Ca	8.1923752004	2.9730316568	5.8287222925			
Ca	8.0577595982	8.3995896112	5.9665706771			
Ca	2.6474700000	2.5105950000	1.9196440000	0	0	0
Ca	2.6474700000	8.0080540000	1.9196440000	0	0	0
Ca	8.0417380000	2.5105950000	1.9196440000	0	0	0
Ca	8.0417380000	8.0080540000	1.9196440000	0	0	0
Ca	5.2953848491	0.2304541469	5.7868632998			
Ca	5.2924005560	5.7913639946	5.9425305078			
Ca	10.6816744746	0.2127256407	5.8485096660			
Ca	0.0496100000	5.2593300000	1.9196450000	0	0	0
Ca	0.0496100000	10.7567880000	1.9196450000	0	0	0
Ca	5.4438780000	5.2593300000	1.9196450000	0	0	0
Ca	5.4438780000	10.7567880000	1.9196450000	0	0	0
Ti	-0.0028254649	2.6787945563	3.8028687635			
Ti	-0.0239641453	8.1412163989	3.8086640460			
Ti	5.3721968136	2.6557424955	3.8037106662			
Ti	5.4108306947	8.1600537666	3.9117676753			
Ti	0.0000000000	2.7487280000	0.0000000000	0	0	0
Ti	0.1267213048	2.8652085437	7.5583350752			
Ti	0.0000000000	8.2461860000	0.0000000000	0	0	0
Ti	0.1780326590	8.3660055686	7.5272336278			
Ti	5.3942680000	2.7487280000	0.0000000000	0	0	0
Ti	5.5031609434	2.8542791717	7.5563304658			
Ti	5.3942680000	8.2461860000	0.0000000000	0	0	0
Ti	5.3508546914	8.0168235675	8.2992325175			
Ti	2.6953201461	-0.1041928360	3.7914438831			
Ti	2.7453676755	5.4314678382	3.8194330759			
Ti	8.1250841745	-0.1167199223	3.8064546410			
Ti	8.0825343688	5.4129830074	3.7903822982			
Ti	2.6971090000	0.0000000000	0.0000000000	0	0	0
Ti	2.5248399580	0.1542457110	7.6006494943			
Ti	2.6971090000	5.4974580000	0.0000000000	0	0	0
Ti	2.5470847678	5.5980677426	7.5939593580			
Ti	8.0913760000	0.0000000000	0.0000000000	0	0	0
Ti	7.9937977467	0.1278806568	7.5619940283			
Ti	8.0913760000	5.4974580000	0.0000000000	0	0	0
Ti	8.0541035041	5.5440256499	7.5412470092			
O	0.4792721884	2.6617205036	5.7613463080			
O	0.5495193496	8.1644669772	5.7370229016			
O	5.8616221955	2.6340208354	5.7491522042			
O	5.7689597133	8.1916916945	5.6984415866			
O	3.1238100000	0.1116490000	1.9196440000	0	0	0
O	3.1238100000	5.6091080000	1.9196440000	0	0	0
O	8.5180770000	0.1116490000	1.9196440000	0	0	0
O	8.5180770000	5.6091080000	1.9196440000	0	0	0
O	1.1365701735	1.2063077815	3.5096530541			
O	1.1045708226	6.6904990760	3.4944414463			
O	6.5180560630	1.1959593102	3.5225401145			
O	6.5512097749	6.6803115331	3.4960966992			
O	1.1266440000	1.1517870000	0.3184580000	0	0	0
O	1.0850690958	1.1515794049	7.9861554852			
O	1.1266440000	6.6492460000	0.3184580000	0	0	0

O	1.1285947481	6.6472193272	7.9621616689			
O	6.5209110000	1.1517870000	0.3184580000	0	0	0
O	6.4676069666	1.0386549652	7.9453606646			
O	6.5209110000	6.6492460000	0.3184580000	0	0	0
O	6.5988105917	6.6140639552	7.9698611761			
O	4.2781405910	4.3993270587	4.1970369255			
O	4.2483129755	9.9025262890	4.1663129253			
O	9.6559425913	4.3890932754	4.1777876766			
O	9.6766212629	9.8983711009	4.1641758148			
O	1.5631659973	3.9542902819	3.5011369420			
O	1.5547446044	9.4642482495	3.4757955484			
O	6.9700147479	3.9421186221	3.4917054105			
O	6.9687999644	9.4513395417	3.4850150310			
O	1.5704400000	3.9005160000	0.3184570000	0	0	0
O	1.5416745558	3.8955144506	7.9868297767			
O	1.5704400000	9.3979740000	0.3184570000	0	0	0
O	1.6477599175	9.3856192097	7.9235297917			
O	6.9647070000	3.9005160000	0.3184570000	0	0	0
O	6.9584303718	3.8537197658	7.9443892212			
O	6.9647070000	9.3979740000	0.3184570000	0	0	0
O	7.1329252518	9.3344979194	8.2306203531			
O	3.8265376293	1.6522338490	4.1759016321			
O	3.7829879938	7.1282030299	4.1672203029			
O	9.2301901194	1.6330689598	4.1900183935			
O	9.2223793768	7.1547437710	4.2178316691			
O	3.8586268668	1.4597638495	7.3943108627			
O	3.8513373132	6.9589745957	7.4416613793			
O	9.2455505605	1.4611794379	7.3665224177			
O	9.2909078793	6.9384465412	7.3822026418			
O	4.9675650000	2.8603790000	1.9196440000	0	0	0
O	4.9675650000	8.3578380000	1.9196440000	0	0	0
O	4.2220997484	4.1992642032	7.3892251112			
O	4.3990153858	9.8517692041	7.7866733214			
O	9.5915258900	4.2054190583	7.3728807128			
O	9.6464275944	9.6838504491	7.4011186981			
O	2.2345640320	5.3982354217	5.7707888177			
O	7.6329523088	5.3979093680	5.7550350447			
O	3.8237770000	1.5969400000	-0.3184580000	0	0	0
O	3.8237770000	7.0943990000	-0.3184580000	0	0	0
O	9.2180450000	1.5969400000	-0.3184580000	0	0	0
O	9.2180450000	7.0943990000	-0.3184580000	0	0	0
O	7.6105132579	-0.1715269805	5.7779753880			
O	-0.4267020000	2.8603790000	1.9196440000	0	0	0
O	-0.4267020000	8.3578380000	1.9196440000	0	0	0
O	4.2675730000	4.3456700000	-0.3184590000	0	0	0
O	4.2675730000	9.8431280000	-0.3184590000	0	0	0
O	9.6618410000	4.3456700000	-0.3184590000	0	0	0
O	9.6618410000	9.8431280000	-0.3184590000	0	0	0
O	2.2746962914	-0.1076166226	5.7839359129			
C	5.0331844159	7.8183809089	10.1570419282			
H	4.4118744680	8.5159500269	10.7398091804			
H	5.4284801849	6.9907349700	10.7624090017			
H	7.7622511064	8.8108304487	8.7632964725			
H	5.0276162856	10.5089159819	8.1808946478			

CH_3-c2

```
110
CH3_c2
Ca              -0.0551936897         5.7609397450         5.7241882682
Ca               2.8106322674         2.9895347863         5.7417057330
Ca               2.7761271537         8.4846188335         5.7186643341
Ca               8.2020776228         2.9697788284         5.7033056934
Ca               8.2383043152         8.4928546082         5.7219345416
Ca               2.6474700000         2.5105950000         1.9196440000    0    0    0
Ca               2.6474700000         8.0080540000         1.9196440000    0    0    0
Ca               8.0417380000         2.5105950000         1.9196440000    0    0    0
Ca               8.0417380000         8.0080540000         1.9196440000    0    0    0
Ca               5.2941478142         0.2262806115         5.7247672079
Ca               5.2434368084         5.6809846223         5.7532698266
Ca              10.6485141102         0.2419662189         5.7639864453
Ca               0.0496100000         5.2593300000         1.9196450000    0    0    0
Ca               0.0496100000        10.7567880000         1.9196450000    0    0    0
Ca               5.4438780000         5.2593300000         1.9196450000    0    0    0
Ca               5.4438780000        10.7567880000         1.9196450000    0    0    0
Ti               0.0027297268         2.7332726653         3.7746994813
Ti              -0.0022584001         8.2414856756         3.7739732425
Ti               5.3876187844         2.7271192380         3.7780876635
Ti               5.3912202356         8.2146063966         3.7792819056
Ti               0.0000000000         2.7487280000         0.0000000000    0    0    0
Ti               0.1699505798         2.8125459853         7.5730545141
Ti               0.0000000000         8.2461860000         0.0000000000    0    0    0
Ti               0.1461158948         8.3767542352         7.5747755571
Ti               5.3942680000         2.7487280000         0.0000000000    0    0    0
Ti               5.3626141236         2.7057185596         7.5572846559
Ti               5.3942680000         8.2461860000         0.0000000000    0    0    0
Ti               5.3318292359         8.2835621303         7.5544291701
Ti               2.6980500902        -0.0135858596         3.7778677961
Ti               2.7041283247         5.4788847827         3.7763512311
Ti               8.0930316234        -0.0197351012         3.7787347190
Ti               8.1035416772         5.4784871216         3.7787379964
Ti               2.6971090000         0.0000000000         0.0000000000    0    0    0
Ti               2.5234445788         0.0778687717         7.5692639141
Ti               2.6971090000         5.4974580000         0.0000000000    0    0    0
Ti               2.5364777523         5.5626605411         7.6105445334
Ti               8.0913760000         0.0000000000         0.0000000000    0    0    0
Ti               8.0266602140         0.1386123173         7.5899896774
Ti               8.0913760000         5.4974580000         0.0000000000    0    0    0
Ti               8.1798194370         5.5203917002         7.5644659478
O                0.4760637852         2.6670034228         5.7997785399
O                0.4558204448         8.1851717646         5.7941060684
O                5.8626340665         2.6215202138         5.8141039103
O                5.9156139933         8.0464898051         5.8638988369
O                3.1238100000         0.1116490000         1.9196440000    0    0    0
O                3.1238100000         5.6091080000         1.9196440000    0    0    0
O                8.5180770000         0.1116490000         1.9196440000    0    0    0
O                8.5180770000         5.6091080000         1.9196440000    0    0    0
O                1.1409945044         1.1725508286         3.5371552025
O                1.1403405914         6.6645160842         3.5635376194
O                6.5337256308         1.1593282811         3.5503313690
O                6.5386077850         6.6557952838         3.5484018633
O                1.1266440000         1.1517870000         0.3184580000    0    0    0
O                1.1437826718         1.1842303526         8.0183248596
O                1.1266440000         6.6492460000         0.3184580000    0    0    0
```

O	1.1679731146	6.6798826414	7.9918666613			
O	6.5209110000	1.1517870000	0.3184580000	0	0	0
O	6.5786628718	1.1084452573	8.0837991251			
O	6.5209110000	6.6492460000	0.3184580000	0	0	0
O	6.6414170038	6.5178632425	8.4145000058			
O	4.2433654995	4.3027333037	4.2053289563			
O	4.2458694858	9.7945771786	4.2294792441			
O	9.6383923434	4.3217351753	4.2332246451			
O	9.6407366416	9.8164007806	4.2085846179			
O	1.5547102000	3.9227725099	3.5340464267			
O	1.5624992743	9.4120753518	3.5251425408			
O	6.9608971278	3.9098446646	3.5507830046			
O	6.9531958216	9.4049367491	3.5526699007			
O	1.5704400000	3.9005160000	0.3184570000	0	0	0
O	1.5812240793	3.9213343120	7.9924759724			
O	1.5704400000	9.3979740000	0.3184570000	0	0	0
O	1.6135678161	9.4177191594	7.9854737721			
O	6.9647070000	3.9005160000	0.3184570000	0	0	0
O	7.0435813199	3.7454513232	8.2310090854			
O	6.9647070000	9.3979740000	0.3184570000	0	0	0
O	7.0071966560	9.2583347330	8.2413141055			
O	3.8477771153	1.5649126665	4.1923615092			
O	3.8559915805	7.0626929514	4.2002699756			
O	9.2423944719	1.5591041105	4.1964773090			
O	9.2423278533	7.0553210359	4.2011238528			
O	3.9216705978	1.4537795858	7.3955413897			
O	3.9864984349	6.9557294989	7.4849127497			
O	9.2707005711	1.5083894320	7.3876572226			
O	9.2556119756	6.9891534799	7.3788034671			
O	4.9675650000	2.8603790000	1.9196440000	0	0	0
O	4.9675650000	8.3578380000	1.9196440000	0	0	0
O	4.2954645731	4.2032402425	7.4455967758			
O	4.2777925071	9.7216008199	7.3476862134			
O	9.5851480974	4.2414598675	7.4541015372			
O	9.5964387674	9.7059885776	7.4830469863			
O	2.2846529018	5.4193463802	5.8145253895			
O	7.5810927066	5.3607275620	5.8728589755			
O	3.8237770000	1.5969400000	-0.3184580000	0	0	0
O	3.8237770000	7.0943990000	-0.3184580000	0	0	0
O	9.2180450000	1.5969400000	-0.3184580000	0	0	0
O	9.2180450000	7.0943990000	-0.3184580000	0	0	0
O	7.6152047377	-0.1407312180	5.8337202498			
O	-0.4267020000	2.8603790000	1.9196440000	0	0	0
O	-0.4267020000	8.3578380000	1.9196440000	0	0	0
O	4.2675730000	4.3456700000	-0.3184590000	0	0	0
O	4.2675730000	9.8431280000	-0.3184590000	0	0	0
O	9.6618410000	4.3456700000	-0.3184590000	0	0	0
O	9.6618410000	9.8431280000	-0.3184590000	0	0	0
O	2.2063935591	-0.0796290762	5.7943606945			
C	6.1233383878	6.1914933378	9.7092220449			
H	5.0254678979	6.2847432996	9.7072563844			
H	6.3659808319	5.1532804160	9.9774004738			
H	6.5393034388	6.8727042005	10.4701842957			
H	7.6782477456	8.6025083521	8.5234405634			
H	7.6550859355	3.0392916795	8.5263408204			

CH$_3$-c3

110						
CH3_c3						
Ca	-0.0835294823	5.7522050933	5.7375151722			
Ca	2.8411017861	2.9855433386	5.7636139656			
Ca	2.7642564985	8.5082714643	5.7224984809			
Ca	8.1786153417	2.9566593646	5.7235899934			
Ca	8.2431219288	8.4771667348	5.7877423083			
Ca	2.6474700000	2.5105950000	1.9196440000	0	0	0
Ca	2.6474700000	8.0080540000	1.9196440000	0	0	0
Ca	8.0417380000	2.5105950000	1.9196440000	0	0	0
Ca	8.0417380000	8.0080540000	1.9196440000	0	0	0
Ca	5.3150750899	0.3316101518	5.6887887274			
Ca	5.3111456523	5.6267610127	5.6542576109			
Ca	10.6665024868	0.1994055552	5.7584965874			
Ca	0.0496100000	5.2593300000	1.9196450000	0	0	0
Ca	0.0496100000	10.7567880000	1.9196450000	0	0	0
Ca	5.4438780000	5.2593300000	1.9196450000	0	0	0
Ca	5.4438780000	10.7567880000	1.9196450000	0	0	0
Ti	-0.0036998519	2.7217829117	3.7813721629			
Ti	-0.0035679609	8.2291522794	3.7773133017			
Ti	5.3942430172	2.7409920369	3.7669030830			
Ti	5.3809596114	8.2226306318	3.7792156016			
Ti	0.0000000000	2.7487280000	0.0000000000	0	0	0
Ti	0.0813747356	2.7453123998	7.5526900972			
Ti	0.0000000000	8.2461860000	0.0000000000	0	0	0
Ti	0.1701551554	8.3661445635	7.5708832554			
Ti	5.3942680000	2.7487280000	0.0000000000	0	0	0
Ti	5.4923233166	2.8647877234	7.6001126828			
Ti	5.3942680000	8.2461860000	0.0000000000	0	0	0
Ti	5.6780362877	8.2853319529	7.7234199726			
Ti	2.7036295805	-0.0043264489	3.7829766781			
Ti	2.6880342638	5.4823164195	3.7741671452			
Ti	8.1038186103	-0.0289890338	3.7826396693			
Ti	8.1142632035	5.4820270362	3.7863914083			
Ti	2.6971090000	0.0000000000	0.0000000000	0	0	0
Ti	2.5228820273	0.1635867880	7.5964787240			
Ti	2.6971090000	5.4974580000	0.0000000000	0	0	0
Ti	2.4464931227	5.4299945798	7.6522217907			
Ti	8.0913760000	0.0000000000	0.0000000000	0	0	0
Ti	7.9938003822	0.0991313995	7.5620592058			
Ti	8.0913760000	5.4974580000	0.0000000000	0	0	0
Ti	8.1673294296	5.4548041653	7.5520764957			
O	0.4835127443	2.6236960065	5.8023769475			
O	0.4759968857	8.1306264203	5.7842250695			
O	5.8356521812	2.7137215607	5.8041782039			
O	5.8107025875	8.0381311261	5.9545368046			
O	3.1238100000	0.1116490000	1.9196440000	0	0	0
O	3.1238100000	5.6091080000	1.9196440000	0	0	0
O	8.5180770000	0.1116490000	1.9196440000	0	0	0
O	8.5180770000	5.6091080000	1.9196440000	0	0	0
O	1.1498124594	1.1633158076	3.5229022885			
O	1.1363138450	6.6590837323	3.5274009385			
O	6.5316092873	1.1633982374	3.5554148031			
O	6.5171209228	6.6567100159	3.5648275291			
O	1.1266440000	1.1517870000	0.3184580000	0	0	0
O	1.0623013481	1.1338933149	8.0352115042			
O	1.1266440000	6.6492460000	0.3184580000	0	0	0

O	1.1190734090	6.6372987611	8.0162996250			
O	6.5209110000	1.1517870000	0.3184580000	0	0	0
O	6.4518888048	1.0412860208	7.9626478909			
O	6.5209110000	6.6492460000	0.3184580000	0	0	0
O	6.5258512063	6.5295230421	8.3434778316			
O	4.2326394851	4.3119038826	4.1600839095			
O	4.2599974727	9.8054166255	4.2495896389			
O	9.6394100218	4.3119293655	4.1996878512			
O	9.6470150052	9.8073642798	4.1807951031			
O	1.5549127591	3.9215277959	3.5664524672			
O	1.5784645282	9.4035212192	3.5601092690			
O	6.9503455852	3.9189185740	3.5431152636			
O	6.9430122625	9.3915278041	3.5750331038			
O	1.5704400000	3.9005160000	0.3184570000	0	0	0
O	1.5027427232	3.8782756177	8.0487795829			
O	1.5704400000	9.3979740000	0.3184570000	0	0	0
O	1.6691051983	9.3717639353	7.9345679133			
O	6.9647070000	3.9005160000	0.3184570000	0	0	0
O	6.9021901861	3.8930777324	8.0875330737			
O	6.9647070000	9.3979740000	0.3184570000	0	0	0
O	7.0749050156	9.3088168474	8.0766712724			
O	3.8506302528	1.5712109295	4.2048503770			
O	3.8402890995	7.0682393363	4.2435329781			
O	9.2458464848	1.5525514669	4.2109637567			
O	9.2379104006	7.0610364909	4.2156084255			
O	3.8272347567	1.5124058699	7.4177092768			
O	3.8725396378	7.1405981865	7.9447159640			
O	9.2456373494	1.4857850725	7.3844932078			
O	9.2238862796	6.9401415272	7.3960791761			
O	4.9675650000	2.8603790000	1.9196440000	0	0	0
O	4.9675650000	8.3578380000	1.9196440000	0	0	0
O	4.2115249607	4.3106547295	7.4509286300			
O	4.4588245342	9.9277711239	7.7892191796			
O	9.5689903505	4.1991735326	7.3644183588			
O	9.6519957439	9.7074214999	7.3941127570			
O	2.2759888261	5.4373531909	5.8470163060			
O	7.6182587593	5.3688290793	5.8215408452			
O	3.8237770000	1.5969400000	-0.3184580000	0	0	0
O	3.8237770000	7.0943990000	-0.3184580000	0	0	0
O	9.2180450000	1.5969400000	-0.3184580000	0	0	0
O	9.2180450000	7.0943990000	-0.3184580000	0	0	0
O	7.6288975079	-0.1372899377	5.7961340907			
O	-0.4267020000	2.8603790000	1.9196440000	0	0	0
O	-0.4267020000	8.3578380000	1.9196440000	0	0	0
O	4.2675730000	4.3456700000	-0.3184590000	0	0	0
O	4.2675730000	9.8431280000	-0.3184590000	0	0	0
O	9.6618410000	4.3456700000	-0.3184590000	0	0	0
O	9.6618410000	9.8431280000	-0.3184590000	0	0	0
O	2.2583894176	-0.0935350520	5.8111342281			
C	3.6709322581	7.4180996077	9.3430998638			
H	2.7378930987	7.9731803976	9.4921064755			
H	3.6742110995	6.4884426045	9.9445587391			
H	5.8920004900	5.8164186181	8.5742625804			
H	4.4928773274	8.0368312798	9.7705419881			
H	5.0743279368	10.6461326168	8.1108299142			

*CH_3-c4

110
CH3_c4

Ca	-0.1128189417	5.7532716552	5.7787899980			
Ca	2.8440408464	2.9923993493	5.7993467636			
Ca	2.8881573192	8.5069707084	5.8494941487			
Ca	8.1902361938	2.9787466767	5.7755105057			
Ca	8.1411038092	8.4494284318	5.8246296616			
Ca	2.6474700000	2.5105950000	1.9196440000	0	0	0
Ca	2.6474700000	8.0080540000	1.9196440000	0	0	0
Ca	8.0417380000	2.5105950000	1.9196440000	0	0	0
Ca	8.0417380000	8.0080540000	1.9196440000	0	0	0
Ca	5.2955171427	0.2689669502	5.7301240043			
Ca	5.2761443496	5.7506318436	5.8321483972			
Ca	10.6643314639	0.2231448385	5.8000050760			
Ca	0.0496100000	5.2593300000	1.9196450000	0	0	0
Ca	0.0496100000	10.7567880000	1.9196450000	0	0	0
Ca	5.4438780000	5.2593300000	1.9196450000	0	0	0
Ca	5.4438780000	10.7567880000	1.9196450000	0	0	0
Ti	0.0003296916	2.7333143721	3.7946380964			
Ti	-0.0074049913	8.2245622189	3.8012998938			
Ti	5.3815637618	2.7371417279	3.7962716199			
Ti	5.4224981025	8.2256047060	3.9328864880			
Ti	0.0000000000	2.7487280000	0.0000000000	0	0	0
Ti	0.1127061998	2.8466030227	7.5468865751			
Ti	0.0000000000	8.2461860000	0.0000000000	0	0	0
Ti	0.1897311846	8.3776571895	7.5444956380			
Ti	5.3942680000	2.7487280000	0.0000000000	0	0	0
Ti	5.5013730399	2.8642195886	7.5638799267			
Ti	5.3942680000	8.2461860000	0.0000000000	0	0	0
Ti	5.3691887953	7.9876015362	8.0580015258			
Ti	2.6893731680	-0.0306263497	3.7908272573			
Ti	2.7039307998	5.4950695680	3.7937940223			
Ti	8.0952030325	-0.0398933644	3.7947482126			
Ti	8.0782315157	5.4787200099	3.7848675968			
Ti	2.6971090000	0.0000000000	0.0000000000	0	0	0
Ti	2.5202994930	0.1770214938	7.5881414920			
Ti	2.6971090000	5.4974580000	0.0000000000	0	0	0
Ti	2.5079689544	5.5292019726	7.5934861195			
Ti	8.0913760000	0.0000000000	0.0000000000	0	0	0
Ti	7.9934179429	0.1348090954	7.5560596896			
Ti	8.0913760000	5.4974580000	0.0000000000	0	0	0
Ti	8.0690180809	5.5278323616	7.5358046360			
O	0.4909878840	2.6535047162	5.7762356224			
O	0.5340866593	8.1486680469	5.7567879957			
O	5.8557191172	2.6486882387	5.7643290050			
O	5.8189470163	8.1483258416	5.7403808002			
O	3.1238100000	0.1116490000	1.9196440000	0	0	0
O	3.1238100000	5.6091080000	1.9196440000	0	0	0
O	8.5180770000	0.1116490000	1.9196440000	0	0	0
O	8.5180770000	5.6091080000	1.9196440000	0	0	0
O	1.1377376471	1.1830570383	3.5111535699			
O	1.1330189692	6.6688568509	3.5055816114			
O	6.5383107319	1.1751077710	3.5351072696			
O	6.5480020095	6.6581732217	3.5106220312			
O	1.1266440000	1.1517870000	0.3184580000	0	0	0
O	1.0814219670	1.1691423201	8.0133794037			
O	1.1266440000	6.6492460000	0.3184580000	0	0	0

O	1.1649373200	6.6597307435	7.9991785917			
O	6.5209110000	1.1517870000	0.3184580000	0	0	0
O	6.4723901802	1.0381162639	7.9678800867			
O	6.5209110000	6.6492460000	0.3184580000	0	0	0
O	6.5851514654	6.6106162315	8.0462542067			
O	4.2562622795	4.3360687349	4.1996833590			
O	4.2301287838	9.8371358141	4.2021403291			
O	9.6466953845	4.3225342259	4.1957613158			
O	9.6480397046	9.8214211359	4.1974668013			
O	1.5579794604	3.9265702113	3.5156709127			
O	1.5503497494	9.4401208452	3.4997020309			
O	6.9609462214	3.9242794977	3.5061055822			
O	6.9658733049	9.4285467066	3.5106337834			
O	1.5704400000	3.9005160000	0.3184570000	0	0	0
O	1.5401371579	3.9086067144	8.0070172774			
O	1.5704400000	9.3979740000	0.3184570000	0	0	0
O	1.6640360892	9.3939326734	7.9256161599			
O	6.9647070000	3.9005160000	0.3184570000	0	0	0
O	6.9547411659	3.8581021362	7.9722725569			
O	6.9647070000	9.3979740000	0.3184570000	0	0	0
O	7.1196207048	9.2912173588	8.1717510596			
O	3.8454936351	1.5893672306	4.1767123377			
O	3.8162678634	7.0662209119	4.2046523354			
O	9.2487190348	1.5702894145	4.2045163987			
O	9.2448879175	7.0784504502	4.2062148695			
O	3.8518569516	1.4815032037	7.4003629244			
O	3.9022758285	7.0115173028	7.4358710328			
O	9.2496191741	1.4751329655	7.3628672064			
O	9.2710913231	6.9445303278	7.3889815142			
O	4.9675650000	2.8603790000	1.9196440000	0	0	0
O	4.9675650000	8.3578380000	1.9196440000	0	0	0
O	4.2162951455	4.2279089712	7.3928112904			
O	4.4301332670	9.8693019537	7.6728151551			
O	9.5897753919	4.2128540619	7.3693084357			
O	9.6494096190	9.6887964133	7.4106905210			
O	2.2208268774	5.3957858107	5.7962887864			
O	7.6232850048	5.3940247690	5.7713541017			
O	3.8237770000	1.5969400000	-0.3184580000	0	0	0
O	3.8237770000	7.0943990000	-0.3184580000	0	0	0
O	9.2180450000	1.5969400000	-0.3184580000	0	0	0
O	9.2180450000	7.0943990000	-0.3184580000	0	0	0
O	7.6145289750	-0.1456515638	5.7850154959			
O	-0.4267020000	2.8603790000	1.9196440000	0	0	0
O	-0.4267020000	8.3578380000	1.9196440000	0	0	0
O	4.2675730000	4.3456700000	-0.3184590000	0	0	0
O	4.2675730000	9.8431280000	-0.3184590000	0	0	0
O	9.6618410000	4.3456700000	-0.3184590000	0	0	0
O	9.6618410000	9.8431280000	-0.3184590000	0	0	0
O	2.2510098200	-0.0806642467	5.7842525861			
C	4.9650899003	8.0525199883	10.1233883573			
H	4.0483197492	8.6299730076	10.3110258450			
H	4.8341568698	7.0262925250	10.4983980702			
H	5.8094129448	8.5189914781	10.6529615701			
H	5.0412277812	10.5219474594	8.0990260079			
H	7.8207149046	8.7025577726	8.5168634947			

CH_4

110
CH4

Ca	-0.0945055462	5.7201916584	5.8632568653			
Ca	2.8064120244	2.9963016004	5.8728913016			
Ca	2.7980209381	8.4630560274	5.8323830216			
Ca	8.1948435975	2.9555176739	5.8607561025			
Ca	8.2220373464	8.4726824149	5.8616994763			
Ca	2.6474700000	2.5105950000	1.9196440000	0	0	0
Ca	2.6474700000	8.0080540000	1.9196440000	0	0	0
Ca	8.0417380000	2.5105950000	1.9196440000	0	0	0
Ca	8.0417380000	8.0080540000	1.9196440000	0	0	0
Ca	5.2820547083	0.2886743356	5.7780845857			
Ca	5.2693534775	5.7042653683	5.8486938877			
Ca	10.6573907285	0.2244690206	5.8669643137			
Ca	0.0496100000	5.2593300000	1.9196450000	0	0	0
Ca	0.0496100000	10.7567880000	1.9196450000	0	0	0
Ca	5.4438780000	5.2593300000	1.9196450000	0	0	0
Ca	5.4438780000	10.7567880000	1.9196450000	0	0	0
Ti	-0.0210121233	2.6690482478	3.7950891841			
Ti	-0.0170146771	8.1628513753	3.7878691340			
Ti	5.3769275421	2.6836550159	3.7877943420			
Ti	5.3828390901	8.1655444929	3.7951333120			
Ti	0.0000000000	2.7487280000	0.0000000000	0	0	0
Ti	0.1210053363	2.8722040489	7.5668321633			
Ti	0.0000000000	8.2461860000	0.0000000000	0	0	0
Ti	0.1431134284	8.3679991345	7.5556900356			
Ti	5.3942680000	2.7487280000	0.0000000000	0	0	0
Ti	5.5020693404	2.8663277685	7.5691230590			
Ti	5.3942680000	8.2461860000	0.0000000000	0	0	0
Ti	5.6404593251	8.1872375040	7.6413807396			
Ti	2.7060709986	-0.0724253721	3.7933883414			
Ti	2.7086608991	5.4231184701	3.7889433861			
Ti	8.1070666077	-0.0834550245	3.7924694048			
Ti	8.1144308328	5.4167100167	3.7924563843			
Ti	2.6971090000	0.0000000000	0.0000000000	0	0	0
Ti	2.5162016030	0.1592239951	7.5956902002			
Ti	2.6971090000	5.4974580000	0.0000000000	0	0	0
Ti	2.5929297001	5.6453105284	7.5625226086			
Ti	8.0913760000	0.0000000000	0.0000000000	0	0	0
Ti	7.9484225664	0.1035324519	7.5618847005			
Ti	8.0913760000	5.4974580000	0.0000000000	0	0	0
Ti	8.0398139907	5.5565656040	7.5456249644			
O	0.4510960589	2.6594278765	5.7677389508			
O	0.4751214378	8.1467708441	5.7526062102			
O	5.8533998230	2.6660286516	5.7614572744			
O	5.8459444692	8.1657170695	5.8117370133			
O	3.1238100000	0.1116490000	1.9196440000	0	0	0
O	3.1238100000	5.6091080000	1.9196440000	0	0	0
O	8.5180770000	0.1116490000	1.9196440000	0	0	0
O	8.5180770000	5.6091080000	1.9196440000	0	0	0
O	1.1352304945	1.1977364932	3.5167246455			
O	1.1377793232	6.7031151012	3.4894751215			
O	6.5193838576	1.1988672149	3.5160301173			
O	6.5263278295	6.7002047939	3.5163081342			
O	1.1266440000	1.1517870000	0.3184580000	0	0	0
O	1.0914673061	1.1778726329	8.0037729143			
O	1.1266440000	6.6492460000	0.3184580000	0	0	0

O	1.1610949218	6.6723141473	7.9822763658			
O	6.5209110000	1.1517870000	0.3184580000	0	0	0
O	6.4500044743	1.0758955143	7.9725550393			
O	6.5209110000	6.6492460000	0.3184580000	0	0	0
O	6.5437440238	6.6043016739	8.0363178222			
O	4.2536762339	4.3802718972	4.1780589012			
O	4.2496763886	9.8736615605	4.2076461713			
O	9.6476997323	4.3764763090	4.1915083132			
O	9.6436066337	9.8699296922	4.1899689451			
O	1.5503875683	3.9568598413	3.5101685385			
O	1.5570883902	9.4512026521	3.5067516491			
O	6.9504032752	3.9533484637	3.5008113882			
O	6.9425740258	9.4476627537	3.5020617479			
O	1.5704400000	3.9005160000	0.3184570000	0	0	0
O	1.5314210812	3.9316647344	7.9360926455			
O	1.5704400000	9.3979740000	0.3184570000	0	0	0
O	1.6092785077	9.3983589716	7.9204903871			
O	6.9647070000	3.9005160000	0.3184570000	0	0	0
O	6.9442094524	3.8700846271	7.9661090998			
O	6.9647070000	9.3979740000	0.3184570000	0	0	0
O	6.9876190449	9.3498899384	7.9937360050			
O	3.8297679518	1.6333460528	4.1868905260			
O	3.8309294545	7.1322207690	4.1803153444			
O	9.2208319797	1.6249166865	4.1807105677			
O	9.2271635684	7.1235517496	4.1821302796			
O	3.8477290961	1.4721038988	7.3827289626			
O	3.8917382748	7.0248120796	7.3613256330			
O	9.2321192578	1.4472192528	7.3941449677			
O	9.2782731851	6.9368743084	7.3836483419			
O	4.9675650000	2.8603790000	1.9196440000	0	0	0
O	4.9675650000	8.3578380000	1.9196440000	0	0	0
O	4.2028306363	4.2291238170	7.4001683241			
O	4.3429146933	9.8667301722	7.8369563673			
O	9.5676167895	4.2148791477	7.3877051242			
O	9.6189388345	9.6907212935	7.3856952900			
O	2.2434867408	5.4480447927	5.7603631531			
O	7.6180327780	5.4117313732	5.7674610456			
O	3.8237770000	1.5969400000	-0.3184580000	0	0	0
O	3.8237770000	7.0943990000	-0.3184580000	0	0	0
O	9.2180450000	1.5969400000	-0.3184580000	0	0	0
O	9.2180450000	7.0943990000	-0.3184580000	0	0	0
O	7.6042323384	-0.1002304046	5.7596290359			
O	-0.4267020000	2.8603790000	1.9196440000	0	0	0
O	-0.4267020000	8.3578380000	1.9196440000	0	0	0
O	4.2675730000	4.3456700000	-0.3184590000	0	0	0
O	4.2675730000	9.8431280000	-0.3184590000	0	0	0
O	9.6618410000	4.3456700000	-0.3184590000	0	0	0
O	9.6618410000	9.8431280000	-0.3184590000	0	0	0
O	2.2573238016	-0.1010595380	5.7872990640			
C	3.8425345745	7.5307467642	10.6884398080			
H	3.0872872264	7.9393291040	10.0069565823			
H	3.3589892028	7.0803870198	11.5629487762			
H	4.4342405447	6.7637018000	10.1709043524			
H	4.5072975696	8.3366406223	11.0228141954			
H	4.9847232620	10.5266322910	8.2025062005			

References

[1] Timrov, I.; Marzari, N.; Cococcioni, M. HP–A code for the calculation of Hubbard parameters using density-functional perturbation theory. *Computer Physics Communications* **2022**, *279*, 108455.
